# Insomnia and poor sleep quality in refugee and asylum-seeking populations: A systematic review and meta-analysis

**DOI:** 10.1371/journal.pone.0352964

**Published:** 2026-07-02

**Authors:** Lucca Passow Carpinelli, Luis Eduardo Gauer, Victor Henrique Dominiak Soares, Simone Blythe Williams, Caroline Baldini Szepeilewicz, Julia Valle Pezzini, Antônio Oesir Gonçalves Neto, Sidarta Tollendal Gomes Ribeiro

**Affiliations:** 1 Federal University of Paraná (UFPR), Curitiba, Paraná, Brazil; 2 State University of Campinas (UNICAMP), Campinas, São Paulo, Brazil; 3 Brain Institute of UFRN (ICe-UFRN), Natal, Rio Grande do Norte, Brazil; 4 Center for Strategic Studies of Oswaldo Cruz Foundation (CEE-Fiocruz), Rio de Janeiro, Rio de Janeiro, Brazil; University of Nebraska Medical Center College of Medicine, UNITED STATES OF AMERICA

## Abstract

Refugees and asylum seekers face significant mental health challenges, yet sleep disturbances remain underrecognized despite their critical impact on well-being. This systematic review and meta-analysis assessed sleep quality and insomnia severity across 66 studies (n = 42,956). Pooled analyses of the Insomnia Severity Index (ISI) and Pittsburgh Sleep Quality Index (PSQI) based on studies identified in Cochrane, Embase, and PubMed from database inception to December 2024 revealed clinically significant sleep disturbances. The pooled mean ISI score (13.76, 95% CI 10.39–17.13) falls within the upper end of the subthreshold range, bordering on moderate clinical insomnia, while PSQI scores (8.59, 95% CI 2.11–15.07) exceeded clinical thresholds for poor sleep. The pooled prevalence of sleep adversities was 43.2% in adults and 36.4% in children. Secondary findings highlighted prolonged sleep latency and frequent nightmares. Although subgroup analyses suggested trends across populations and assessment methods, statistical significance was limited by sample heterogeneity. Standardized sleep assessments must be integrated into refugee health protocols, with targeted interventions addressing insomnia risk factors.

## Introduction

Forced displacement remains a pressing global issue with profound social, economic, and political implications. Refugees, as defined by the 1951 Refugee Convention, are individuals unable to return to their country of origin due to a well-founded fear of persecution, while asylum seekers are those seeking international protection, either awaiting or intending to apply for asylum [1]. Humanitarian aid, access to employment, and education remain critical pathways to improving health outcomes, emphasizing the importance of evidence-based, coordinated approaches that align with the needs of displaced populations [2].

By the end of 2025, approximately 117.3 million individuals were forcibly displaced worldwide, corresponding to roughly 1 in 70 people globally, with 71% residing in low- and middle-income countries, highlighting the disproportionate burden faced by these regions [3]. At the same time, approximately 8.4 million asylum seekers were awaiting decisions on their applications, underscoring the urgent need for effective international protection systems [4].

The refugee experience is highly heterogeneous and shaped by multiple factors, including pre-migration trauma, conditions during displacement, and post-migration stressors such as socioeconomic hardship, discrimination, and social isolation. Among these factors, exposure to violence and trauma during migration has been associated with poorer sleep quality and increased PTSD, depression, and anxiety symptoms [5]. These challenges often occur alongside limited access to healthcare and other essential services, contributing to persistent health disparities even in high-income host countries [6].

Sleep disturbances represent an important yet often underrecognized component of refugee health. Insomnia, which frequently develops following traumatic experiences, may exacerbate health problems and contribute to cardiovascular disease, pain, depression, and fatigue [7–10]. Sleep is now recognized as an active and essential biological process supporting cognitive and emotional functioning, and increasing evidence suggests that sleep disturbances may function as transdiagnostic markers of mental health conditions [11]. Addressing sleep disturbances may therefore represent an important pathway to improving mental health outcomes in displaced populations.

Assessing sleep and mental health problems in refugee populations presents important methodological challenges [12]. Self-report tools, frequently used due to their cost-effectiveness in large or resource-limited settings, may overestimate prevalence because of high sensitivity cut-offs, limited cultural adaptation, and response biases [13]. Although clinical diagnostic interviews conducted by trained clinicians may improve the assessment of psychiatric disorders in epidemiological studies, accurately evaluating mental health remains challenging in refugee populations, particularly when diagnostic instruments developed in Western contexts are applied across culturally diverse groups [14].

Despite the growing recognition of sleep disturbances in displaced populations, the available evidence remains fragmented. A previous systematic review reported a high prevalence of sleep disturbances among migrants and refugees and highlighted the association between sleep problems and trauma-related experiences in these populations [15]. More recent narrative evidence has similarly emphasized the high prevalence of insomnia and nightmares among refugees and their association with conditions such as PTSD, depression, and anxiety [16]. However, the available literature remains heterogeneous in study design, populations, and assessment methods, and lacks a comprehensive quantitative synthesis across refugee and asylum seeker populations.

To address these gaps, this systematic review and meta-analysis investigates sleep quality, insomnia, and related sleep disturbances among refugees and asylum seekers. The primary outcomes include sleep quality measured using the Pittsburgh Sleep Quality Index (PSQI) [17] and insomnia severity assessed with the Insomnia Severity Index (ISI) [18]. Secondary outcomes include sleep adversities and related sleep conditions such as nightmares. This study provides, to our knowledge, the first comprehensive quantitative synthesis examining sleep quality and insomnia outcomes among refugee and asylum-seeking populations, aiming to provide a comprehensive understanding of sleep health in this vulnerable population.

## Materials and methods

### Study design and protocol registration

This systematic review and meta-analysis was conducted according to the Preferred Reporting Items for Systematic Reviews and Meta-analyses [19,20] guidelines and the Meta-analyses Of Observational Studies in Epidemiology (MOOSE) checklist [21]. The protocol was prospectively registered in PROSPERO (CRD42024605014) prior to data extraction and statistical analysis.

### Amendments to the registered protocol

While our analysis largely adhered to the PROSPERO protocol, we implemented several methodological refinements: (1) We expanded our planned subgroup analysis beyond age categories to include assessment methods, healthcare access, and geographic factors to better explore heterogeneity sources; (2) We incorporated meta-regression analyses using R’s {metafor} package within an information-theoretic framework, which was not explicitly specified in the original protocol; (3) We employed an adapted Newcastle-Ottawa Scale for cohort and cross-sectional studies, as it proved more suitable for our included study designs; and (4) Although interventional studies were considered eligible if extractable baseline data were available, following peer-review feedback we conducted all pooled prevalence and mean analyses excluding baseline data from interventional studies to minimize potential design-related bias, retaining these studies in the descriptive synthesis only.

### Eligibility criteria

#### Inclusion criteria.

Studies were eligible for inclusion if they met the following criteria: (1) peer-reviewed observational studies (cross-sectional, cohort, or case-control designs) and interventional studies, although only observational studies contributed to pooled prevalence and mean estimates; (2) participants comprised refugees and asylum seekers, as defined by the United Nations High Commissioner for Refugees, with no restrictions on age, country of origin, or resettlement; (3) outcomes included: (a) insomnia severity assessed via the Insomnia Severity Index (ISI); (b) sleep quality assessed via the Pittsburgh Sleep Quality Index (PSQI); (c) prevalence of sleep adversities, defined as a broad construct aggregating heterogeneous sleep disturbances, including insomnia symptoms and other structured reports of sleep problems; and (d) specific sleep outcomes analyzed separately, including nightmare prevalence, sleep duration, sleep latency, and other standardized quantitative measures of sleep quality, including Likert-type scale assessments standardized for meta-analytic pooling; and (4) no language restrictions were applied.

#### Exclusion criteria.

We excluded the following types of studies: (1) reviews, editorials, dissertations, or conference presentations; (2) studies with duplicated samples or data; (3) studies that did not report outcomes of interest; (4) studies involving non-refugee or mixed populations; (5) studies with small sample sizes (N < 30), as very small samples may yield unstable prevalence estimates and disproportionately influence pooled analyses; (6) studies from which relevant outcome data could not be extracted and qualitative studies; (7) studies involving migrants with chronic diseases known to be associated with the onset of secondary mental health disorders, such as cancer, cardiovascular disease, and other chronic conditions, to reduce clinical confounding [22]; and (8) studies restricted to Internally Displaced Persons (IDPs), as they diverged from the target population of this study.

### Search strategy

We systematically searched Cochrane Library, Embase, and PubMed for relevant studies, with an initial search conducted on July 15, 2024 and an updated search on December 18, 2024. Two investigators (LC and AG) developed and executed the search strategy, which incorporated both controlled vocabulary terms (MeSH for PubMed and Emtree for Embase) and free-text terms. Search terms combined concepts related to displacement (“refugee,” “asylum seeker,” “migrant”) with terms for sleep disorders (“insomnia,” “sleep disturbances,” “nightmares”). To ensure comprehensive coverage, we additionally: consulted expert recommendations, manually screened reference lists of retrieved articles, and reviewed systematic reviews on related topics. The complete list of search strategies is given in the S1 Table.

Two independent reviewers (LC and AG) screened article titles and abstracts in a double-blind manner using the Rayyan software [23], applying the predefined eligibility criteria. Full texts of potentially eligible studies were then retrieved and independently assessed by two pairs of independent reviewers (LC and CS; JP and SW). Discrepancies at any stage of the process were resolved through discussion or consultation with a third reviewer (CS or LG). Final inclusion of each article required unanimous agreement among the review team members. In cases where multiple articles utilized the same sample and met the inclusion criteria, we selected the article that addressed the most outcomes, had the largest sample size, or was the most recent publication.

### Data extraction

Data extraction was conducted using a standardized form developed a priori, adapted from Excel-based templates employed by the authors in prior systematic reviews. Six independent reviewers (LC, CS, JP, SW, LG, VD) extracted the following variables: (1) study characteristics**,** including author(s), publication year, study design, follow-up duration, inclusion criteria keywords, exposure type, intervention details (if applicable), inclusion of PTSD patients, availability of health establishments, host country residence type, legal status, host country income, predominant trauma, reporter type, education level, unemployment rate, family separation, school attendance, unaccompanied minor and sample sizes; (2) population characteristics**,** such as age, sex/gender, origin/host macroregions and countries, and years since resettlement; (3) methodological characteristics**,** including sampling method and recruitment strategy; and (4) outcome measures**,** such as measurement instruments, procedural descriptions, cut-off scores, means, prevalences, and standard errors. To ensure data accuracy, all extracted information underwent independent verification by co-authors, and corresponding authors were contacted when necessary to clarify missing or ambiguous data. For synthesis purposes, studies were grouped according to their reported outcomes and study design. When multiple measures of the same outcome were reported, we prioritized the most comprehensive or clinically relevant timepoint, while data from all instruments were extracted and analyzed separately in subgroup analyses.

Within the literature, sleep conditions exist on a spectrum of diagnostic specificity. A sleep disorder (e.g., Insomnia Disorder) represents a formal diagnosis per standard manuals (DSM-5, ICSD-3), while a sleep disturbance is a broader symptom or complaint that may not meet full diagnostic criteria. To encompass the wide range of non-standardized sleep problems reported across the heterogeneous studies in our review, we employ the umbrella term ‘sleep adversities.’ This category includes specific disturbances like insomnia symptoms, unrestorative sleep, and night waking. Furthermore, when specified, ‘poor sleep quality’ refers specifically to data derived from the Pittsburgh Sleep Quality Index (PSQI) or equivalent instruments.

### Quality assessment

The methodological quality of the included studies was independently assessed by two reviewer pairs (LC and CS; JP and SW) using the Risk of Bias 2.0 (RoB-2) tool [24] for RCTs, and the Newcastle-Ottawa Scales (NOS) for cohort [25] and an adapted NOS version for cross-sectional studies [26]. Any disagreements between reviewers were resolved through discussion, with input from a third independent reviewer when necessary. The RoB-2 tool evaluates randomization process, deviations from intended interventions, missing outcome data, measurement of the outcome and selection of the reported result. The NOS tool evaluates selection, comparability and outcome domains. To standardize the risk of bias for further analyses, the following categories were applied: for cohort studies, good quality was considered low risk, fair quality as some concerns, and poor quality as high risk; for cross-sectional studies, very good quality was classified as low risk, satisfactory and good studies as some concerns, and unsatisfactory studies as high risk; for RCTs, the categories remained the same. The full versions of the scales used in this study can be found in the Tables in the S2 and S3 Tables.

### Data synthesis and statistical analyses

#### Meta-analyses.

To minimize design-related bias in prevalence and mean estimation, pooled analyses were restricted to observational studies. Interventional studies were included in the systematic review and summarized descriptively but were not entered into meta-analyses (even when baseline data were available). Random-effects models were estimated using inverse-variance weighting. Between-study heterogeneity (τ²) was estimated using the Maximum Likelihood method for prevalence models and Restricted Maximum Likelihood for mean models. In order to stabilize the variance, we performed both logit and arcsine transformation for prevalence data, in different models, considering the sensitivity analyses. We back-transformed the pooled estimates to percentages for ease of interpretation. Results are presented as forest plots displaying individual study estimates alongside pooled effects, with heterogeneity statistics (I² and τ²). All analyses were conducted in RStudio (version 2023.12.1.402).

#### Assessment of heterogeneity.

To assess and quantify heterogeneity, we employed both the Q statistic and the I² index, following guidelines from Borenstein and colleagues [27]. The Q statistic was used to test the null hypothesis of homogeneity across studies. A statistically significant Q value indicated the presence of heterogeneity, confirming the appropriateness of our random-effects model approach. Additionally, we calculated the I² statistic to estimate the extent of heterogeneity that is not attributable to sampling error. Higher I² values indicate greater heterogeneity, with values of 30%, 50%, and 75% suggesting low, moderate, and high heterogeneity, respectively. In case of substantial heterogeneity (I² > 75%), we explored potential sources through influence analyses, subgroup analyses and meta-regression [28].

#### Influence analysis.

To assess the robustness of our findings and identify potentially influential studies, we conducted a series of sensitivity analyses. First, we employed graphical methods, including influence plots and Baujat plots, to visually inspect the contribution of individual studies to the overall heterogeneity and pooled effect. Additionally, we performed leave-one-out meta-analyses, systematically excluding each study in turn to evaluate its impact on the pooled effect estimate. For studies identified as potentially influential through these methods, we conducted a detailed examination of their characteristics and methodological quality. We also performed subgroup analyses excluding these studies to assess their impact on the overall conclusions.

#### Subgroup analyses and meta-regressions.

Moderators were evaluated using subgroup or meta-regression analyses if there were ≥10 studies available for analysis [29]. We conducted exploratory subgroup analyses based on assessment methods and availability of health establishments in host regions. To explore potential sources of heterogeneity, we undertook an information-theoretic approach for model selection without strong a priori hypotheses [30]. We conducted multi-model inference using the {mice} package [31] for imputation and the {metafor} package [32] for meta-regression, with model selection via the {MuMIn} package [33]. For prevalence outcomes, meta-regression models were fitted on the logit-transformed proportion scale (metafor measure = “PLO”), and results were back-transformed for interpretation. Given the exploratory nature of our analysis, we further developed an interactive interface to systematically investigate different model combinations, evaluating their performance based on multiple criteria (I², R², k, and AIC). Examined moderators included instrument type, reason of displacement, health establishment availability, origin region, host region, percentage of women, years since settlement, and age on the pooled sleep adversity prevalence estimates.

### Publication bias

Publication bias was assessed through visual inspection of funnel plots and statistical tests (Egger’s test or equivalent) when at least 10 studies [34] were available for analysis. In order to assess the potential impact of publication bias, we employed the Rücker’s limit meta-analysis method [35], since other diagnostic statistics (such as Trim-and-Fill) are unstable under very high heterogeneity [36].

## Results

### Study selection and characteristics

**Fig 1:**
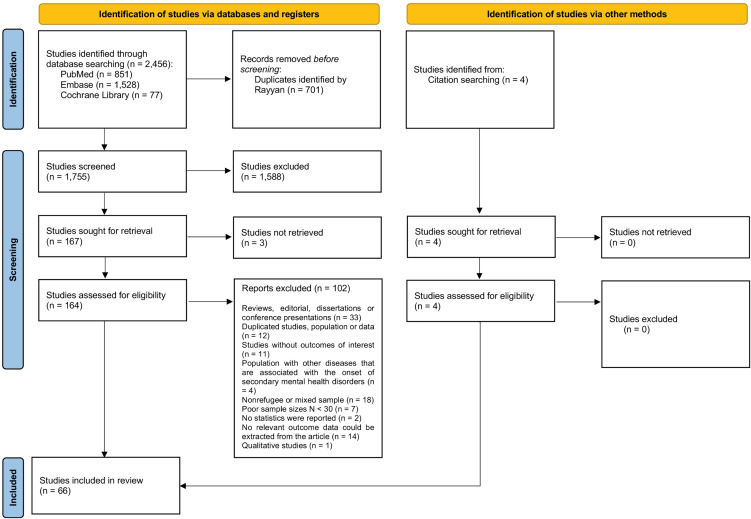
PRISMA flow diagram.

Our systematic search identified 2,456 records from electronic databases supplemented by 4 additional records from manual citation searching. After removing duplicates, we screened 1,759 records and assessed 171 full-text articles for eligibility (Fig 1), though 3 studies could not be retrieved despite contacting authors. We excluded 102 studies for various reasons: 33 were ineligible publication types (reviews, editorials, dissertations or conference presentations); 12 had duplicated populations or data; 11 lacked relevant outcomes; 18 involved non-refugee or mixed populations; 4 included populations with comorbid conditions affecting mental health; 7 had small sample sizes (N < 30); 2 lacked sufficient statistical reporting; 14 had unavailable outcome data; and 1 used qualitative methodology. Ultimately, 66 studies [37–102] met our inclusion criteria, comprising 42,956 participants across multiple countries (Table 1). Geographically, refugees most commonly originated from Western Asia (16.7%, k = 11), followed by South-eastern Asia (10.6%, k = 7), and Sub-Saharan Africa (9.1%, k = 6). The primary host regions were Northern America (24.2%, k = 16), Western Europe (19.7%, k = 13), and Northern Europe (15.2%, k = 10). Nineteen studies (28.8%) included data on children and adolescents, with sample sizes ranging from 32 to 14,303 participants (mean = 650.84; median = 148). Woman representation varied substantially, with four studies (6.1%) reporting exclusively female participants, while population mean age ranged from 5.93 to 62.40 years (mean = 29.87; median = 31.90). Time since settlement was unreported in 53.0% (k = 35) of studies. Among those reporting this variable (k = 31), medium-term (18.2%, k = 12) and long-term settlement (18.2%, k = 12) were equally represented, while short-term and immediate stays were less frequent (10.6%, k = 7). Healthcare access was unavailable in 37.9% (k = 25) of studies, available in 36.4% (k = 24), mixed in 9.1% (k = 6), and unreported in 16.7% (k = 11). Beyond these variables, only 9% (k = 6) of studies reported follow-up data, 5% (k = 3) specified PTSD-related inclusion or exclusion criteria, and only about half of the studies provided information on education (52%, k = 34), while reporting on unemployment (33%, k = 22) and family separation (38%, k = 25) was even less frequent. The predominant nature of exposure was conflict or war (65.2%, k = 43), followed by mixed or unspecified trauma (30.3%, k = 20) and persecution or human rights violations (4.5%, k = 3). A detailed overview of reporting frequencies and literature gaps for all moderators is provided in S4Table.

**Table 1:** Characteristics of included studies.

Study	Study design	Origin sub-regions or regions^1^	Nature of exposure	Host sub-regions or regions^1^	Healthcare availability	Adult sample (n)	Pediatrics sample (n)	Females (%)	Age in years M (SD)	Duration of stay^2^	Sleep assessment method(s)	Risk of bias
Spanhel K, 2022 [37]	Randomized controlled trial	Western Asia, Southern Asia, Sub-Saharan Africa, Northern Africa	Mixed or unspecified trauma	Western Europe	Mixed access	66	0	27.3	28.5 (6.8)	Medium term	ISI; PSQI; FOSI-SF	High
Sankari S, 2023 [38]	Exposure cross-sectional	Western Asia	Conflict or war	Northern America	Without access	53	0	50.9	NR	Short term	PSQI	Some concerns
Richter K, 2018 [39]	Exposure cross-sectional	Southern Asia, Eastern Europe, Western Asia	Mixed or unspecified trauma	Western Europe	Mixed access	283	0	44.2	31.9 (10.6)	NR	PSQI; Custom Questionnaire	Some concerns
Özdemir PG, 2021 [40]	Exposure cross-sectional	Western Asia	Conflict or war	Western Asia	Available access	72	0	45.8	33.01 (9.82)	NR	PSQI	Some concerns
Lee J, 2021 [41]	Exposure cross-sectional	Eastern Asia	Mixed or unspecified trauma	Eastern Asia	NR	32	0	65.6	33.78 (14.33)	Long term	PSQI; Polysomnography	Some concerns
Sandahl H, 2020 [42]	Randomized controlled trial	Southern Asia, Western Asia	Conflict or war	Northern Europe	Mixed access	219	0	49.8	44.4 (10.4)	Long term	PSQI	Some concerns
Schumm H, 2023 [43]	Exposure cross-sectional	Southern Asia, Western Asia	Conflict or war	Western Europe	Mixed access	67	0	31.3	31.12 (11.25)	Medium term	PSQI	Some concerns
Bruck D, 2021 [44]	Exposure cross-sectional	Sub-Saharan Africa	Conflict or war	Australia and New Zealand	NR	117	0	47	35.04 (9.76)	Long term	ISI	Some concerns
Lies J, 2021 [45]	Exposure cross-sectional	Western Asia	Conflict or war	Australia and New Zealand	NR	86	0	51.2	45.41 (16.39)	Short term	ISI; Actigraphy	Low
Al-Smadi AM, 2019 [46]	Exposure cross-sectional	Western Asia	Conflict or war	Western Asia	Mixed access	373	0	62.5	42.73 (14.26)	NR	ISI	Some concerns
Meurling J, 2023 [47]	Exposure cross-sectional	Western Asia, Southern Asia, Sub-Saharan Africa, Northern Africa, NR	Conflict or war	Northern Europe	NR	757	0	37.4	32 (11)	Medium term	ISI	Some concerns
Park J, 2019 [48]	Exposure cross-sectional	Eastern Asia	Mixed or unspecified trauma	Eastern Asia	NR	74	0	63.5	18.7 (2.5)	Long term	ISI	Some concerns
Gammoh OS, 2024a [49]	Exposure cross-sectional	Western Asia	Conflict or war	Western Asia	Available access	291	0	100	NR	Long term	ISI	Some concerns
Lies J, 2019 [50]	Exposure cross-sectional	Western Asia, South-eastern Asia, Southern Asia	Mixed or unspecified trauma	Australia and New Zealand	Available access	1892	811	14	29.67 (16.8)	Short term	Custom Questionnaire	Some concerns
Carlsson JM, 2006 [51]	Prospective cohort	Western Asia, Southern Asia	Conflict or war	Northern Europe	Available access	139	0	9.4	44.7 (8.48)	Long term	HTQ	High
Aldukhail S, 2023 [52]	Mixed observational	Western Asia, Southern Asia	Conflict or war	Northern America	Available access	46	22	11.8	31.71 (16.57)	NR	Custom Questionnaire	High
Rizzi D, 2022 [53]	Exposure cross-sectional	Eastern Europe	Conflict or war	Eastern Europe	Without access	623	0	79.8	36.4 (15.53)	Immediate	Custom Questionnaire	Some concerns
Parvez A, 2023 [54]	Retrospective cohort	Sub-Saharan Africa, Western Asia, Southern Asia	Mixed or unspecified trauma	Northern America	Available access	779	0	46.7	33.2 (12.4)	Long term	Interview	High
Trohl U, 2021 [55]	Retrospective cohort	Southern Asia, Western Asia, Eastern Europe, Sub-Saharan Africa, Southern Europe	Mixed or unspecified trauma	Western Europe	Available access	437	0	39.1	30.7 (NR)	NR	Interview; Custom Questionnaire	High
Tay AK, 2015 [56]	Exposure cross-sectional	South-eastern Asia	Conflict or war	Melanesia	NR	230	0	40.4	37 (9.8)	Long term	Custom Questionnaire	Some concerns
Tamblyn JM, 2011 [57]	Retrospective cohort	Africa	Persecution or human rights violations	Northern America	Available access	58	0	29.3	34.7 (11.5)	NR	Custom Questionnaire	Some concerns
Loutan L, 1999 [58]	Exposure cross-sectional	Southern Europe, Sub-Saharan Africa, Southern Asia	Mixed or unspecified trauma	Western Europe	Available access	573	0	36.3	27.33 (8.14)	Immediate	HTQ	Some concerns
Lee YJ, 2016 [59]	Exposure cross-sectional	Eastern Asia	Mixed or unspecified trauma	Eastern Asia	NR	177	0	72.9	38.22 (12.24)	Medium term	Custom Questionnaire	Some concerns
Honkala E, 1992 [60]	Exposure cross-sectional	Sub-Saharan Africa	Mixed or unspecified trauma	Sub-Saharan Africa	Without access	154	37	45.5	23.8 (5.8)	NR	Custom Questionnaire	Some concerns
Westermeyer JJ, 2010 [61]	Exposure cross-sectional	Sub-Saharan Africa	Conflict or war	Northern America	NR	622	0	48.1	37.1 (15.3)	Medium term	HADStress	Some concerns
Gulden A, 2010 [62]	Exposure cross-sectional	Sub-Saharan Africa	Conflict or war	Northern America	NR	512	0	44.9	32.83 (12.54)	Medium term	HADStress	Some concerns
Gowin M, 2017 [63]	Exposure cross-sectional	Latin America and the Caribbean	Persecution or human rights violations	Northern America	NR	45	0	NR	32 (8.7)	NR	Interview; Custom Questionnaire	High
Schlechter P, 2021 [64]	Exposure cross-sectional	Southern Europe	Conflict or war	Western Europe, Southern Europe, Northern Europe	NR	854	0	51.3	41.6 (10.8)	NR	IES-R	Some concerns
Mootoo C, 2019 [65]	Exposure cross-sectional	Northern Africa	Conflict or war	Sub-Saharan Africa	Without access	863	0	65	33.8 (14.4)	NR	Hozun	Some concerns
Schnyder U, 2015 [66]	Exposure cross-sectional	Western Asia, Southern Asia, Southern Europe	Conflict or war	Western Europe	Available access	134	0	21.6	42 (9.9)	Long term	PDS	Some concerns
Weaver TL, 2008 [67]	Exposure cross-sectional	Southern Europe	Conflict or war	Northern America	Available access	35	0	57.1	22.8 (11.7)	Medium term	PSS	Some concerns
Mölsä M, 2014 [68]	Exposure cross-sectional	Sub-Saharan Africa	Conflict or war	Northern Europe	Without access	128	0	58.6	62.4 (24.2)	Long term	Custom Questionnaire	Some concerns
Vinson GA, 2012 [69]	Exposure cross-sectional	Sub-Saharan Africa	Conflict or war	Sub-Saharan Africa	Without access	3802	0	56.3	35.42 (14.89)	NR	PDS	Some concerns
Zaheer K, 2022 [70]	Exposure cross-sectional	Western Asia, Southern Asia, Sub-Saharan Africa	Mixed or unspecified trauma	Southern Europe	Without access	146	0	27.4	45.8 (17.4)	NR	OHQoL	Some concerns
Lindheimer N, 2020 [71]	Exposure cross-sectional	Western Asia, Northern Africa	Conflict or war	Western Europe	Available access	95	0	43.2	33.8 (9.69)	NR	PHQ-9	Some concerns
Giesebrecht J, 2022 [72]	Exposure cross-sectional	Southern Asia, Western Asia, Sub-Saharan Africa, Northern Africa	Conflict or war	Western Europe	Without access	144	0	33.3	31.9 (7.8)	Short term	PHQ-15	Some concerns
Abuali M, 2024 [73]	Exposure cross-sectional	Southern Asia	Conflict or war	Northern America	Available access	0	121	44.6	7.77 (4.22)	NR	Interview	Some concerns
Montgomery E, 2001 [74]	Exposure cross-sectional	Western Asia, Southern Asia	Conflict or war	Northern Europe	NR	0	311	48.6	7.5 (NR)	Immediate	Custom Questionnaire	Some concerns
Pfeiffer E, 2019 [75]	Exposure cross-sectional	Southern Asia, Western Asia, Sub-Saharan Africa, Northern Africa, Southern Europe	Mixed or unspecified trauma	Western Europe	Without access	0	419	9.3	16.34 (1.82)	Short term	CATS	Some concerns
Genton PC, 2019 [76]	Exposure cross-sectional	Sub-Saharan Africa, Southern Asia, Western Asia, Eastern Asia	Mixed or unspecified trauma	Western Europe	Available access	0	109	12.8	16.4 (1.2)	Immediate	Interview	Some concerns
Ceri V, 2016 [77]	Exposure cross-sectional	Western Asia	Conflict or war	Western Asia	Available access	0	42	57.1	12.1 (4.5)	Immediate	Interview	High
Eiset AH, 2020 [78]	Exposure cross-sectional	Western Asia, Eastern Europe, NR, Southern Asia, Sub-Saharan Africa	Mixed or unspecified trauma	Northern Europe	Available access	0	7210	43	7.7 (4,4)	NR	Interview	Some concerns
Schumacher L, 2021 [79]	Prospective cohort	Southern Asia, Western Asia	Conflict or war	Western Europe	Available access	0	366	24.3	15.8 (1.93)	Short term	CRIES-13	Some concerns
Hjern A, 2019 [80]	Exposure cross-sectional	Southern Asia, Sub-Saharan Africa, Western Asia	Conflict or war	Northern Europe	Available access	0	609	25.9	11.6 (NR)	Immediate	Interview	Some concerns
Hjern A, 1991 [81]	Prospective cohort	Latin America and the Caribbean	Persecution or human rights violations	Northern Europe	Without access	0	50	48	5.93 (NR)	Immediate	Interview	Some concerns
Nasıroğlu S, 2018 [82]	Exposure cross-sectional	Western Asia	Conflict or war	Western Asia	Without access	0	136	46.3	11 (3.11)	NR	Interview	Some concerns
Husni M, 2001 [83]	Exposure cross-sectional	Western Asia	Conflict or war	Northern Europe, Northern America	NR	54	0	33.3	35.8 (10.9)	Medium term	Custom Questionnaire	Some concerns
Hinton D. E, 2009 [84]	Exposure cross-sectional	South-eastern Asia	Conflict or war	Northern America	Available access	100	0	60	47.2 (6.2)	NR	SCID	Some concerns
Cernovsky Z, 1988 [85]	Exposure cross-sectional	Eastern Europe	Mixed or unspecified trauma	Western Europe	NR	100	0	18	24.8 (5.3)	NR	Interview	Some concerns
Lee S, 2021 [86]	Exposure cross-sectional	Eastern Asia	Mixed or unspecified trauma	Eastern Asia	Without access	38	0	60.5	29.5 (13.11)	NR	NDQ; Interview	Some concerns
Berkson SY, 2014 [87]	Prospective cohort	South-eastern Asia	Conflict or war	Northern America	Mixed access	126	0	64.3	57.4 (6.15)	NR	HPQ	High
Bronstein I, 2013 [88]	Exposure cross-sectional	Southern Asia	Conflict or war	Northern Europe	NR	0	326	0	16.34 (1.03)	Short term	SHS; RATS	Some concerns
Simich L, 2006 [89]	Exposure cross-sectional	Northern Africa	Conflict or war	Northern America	NR	220	0	43.2	33.8 (9.6)	Medium term	GHQ-12	Some concerns
Müller LRF, 2021 [90]	Mixed observational	Southern Asia, Western Asia, Sub-Saharan Africa	Mixed or unspecified trauma	Western Europe	Without access	0	40	15	17.5 (1.88)	Medium term	CATS; MDMQ	Some concerns
Mangrio E, 2020 [91]	Exposure cross-sectional	Western Asia, Southern Asia	Mixed or unspecified trauma	Northern Europe	Without access	681	0	30	30.5 (NR)	Immediate	GHQ-12	Some concerns
Ku SY, 2006 [92]	Exposure cross-sectional	Eastern Asia	Mixed or unspecified trauma	Eastern Asia	Without access	411	0	100	31.3 (6.2)	Immediate	Custom Questionnaire	Some concerns
Knappe F, 2023 [93]	Randomized controlled trial	Southern Asia, Western Asia	Mixed or unspecified trauma	Southern Europe	Available access	150	0	50	29.1 (9.3)	Medium term	ISI	Some concerns
Itani T, 2017 [94]	Exposure cross-sectional	Eastern Europe	Conflict or war	Western Asia	Without access	0	14303	50.8	NR	NR	Custom Questionnaire	Low
Hinton DE, 2015 [95]	Exposure cross-sectional	South-eastern Asia	Conflict or war	Northern America	Available access	200	0	60.5	NR	NR	Custom Questionnaire	Some concerns
Hinton DE, 2005 [96]	Exposure cross-sectional	South-eastern Asia	Conflict or war	Northern America	Available access	100	0	68	49.1 (5.3)	Long term	CAPS	Some concerns
Gammoh OS, 2024b [97]	Exposure cross-sectional	Western Asia	Conflict or war	Western Asia	Available access	291	0	100	NR	Long term	ISI	Low
Boiko DI, 2024 [98]	Exposure cross-sectional	Eastern Europe	Conflict or war	Western Europe, Eastern Europe, Northern Europe	NR	92	0	83.7	33.58 (9.74)	Short term	ISI	Some concerns
Thabet AA, 1999 [99]	Exposure cross-sectional	Western Asia	Conflict or war	Western Asia	NR	0	239	46	8.9 (NR)	NR	CPTSD-RI	Some concerns
Kinzie JD, 1986 [100]	Exposure cross-sectional	South-eastern Asia	Conflict or war	Northern America	Without access	0	40	37.5	17 (NR)	Medium term	Custom Questionnaire	High
Realmuto GM, 1992 [101]	Exposure cross-sectional	South-eastern Asia	Conflict or war	Northern America	NR	0	47	21.3	17.5 (2.7)	NR	CPTSD-RI	High
Gammoh OS, 2024c [102]	Exposure cross-sectional	Western Asia	Conflict or war	Western Asia	Available access	177	0	100	35.31 (11.27)	NR	ISI	Some concerns

NR = not reported. ^1^Countries classified using the standard country or area codes proposed by the United Nations Statistics Division. ^2^Duration of stay categorized using the average years since settlement in the host country or clearly stated in studies’ participants characteristics: “Immediate” (0–6 months), “Short term” (6 months–2 years), “Medium term” (2–5 years) and “Long term” (>5 years).

### Quality assessment

The risk of bias assessment demonstrated considerable variation in methodological quality across the included studies. Among randomized trials evaluated using the RoB-2 tool, one study was rated as high risk of bias while another raised some concerns. For cohort studies assessed with the Newcastle-Ottawa Scale (NOS), three were classified as poor quality (high risk of bias) and two as fair quality (some concerns). Cross-sectional studies, evaluated using an adapted NOS scale, showed the following distribution: 26 satisfactory, 24 good, 3 very good, and 6 unsatisfactory. Overall, 80.3% (k = 53) of studies presented some concerns regarding risk of bias, while 15.2% (k = 10) were high risk and only 4.5% (k = 3) were low risk. Complete risk of bias assessments for each study are presented in Table 1.

### Primary outcomes

**Fig 2:**
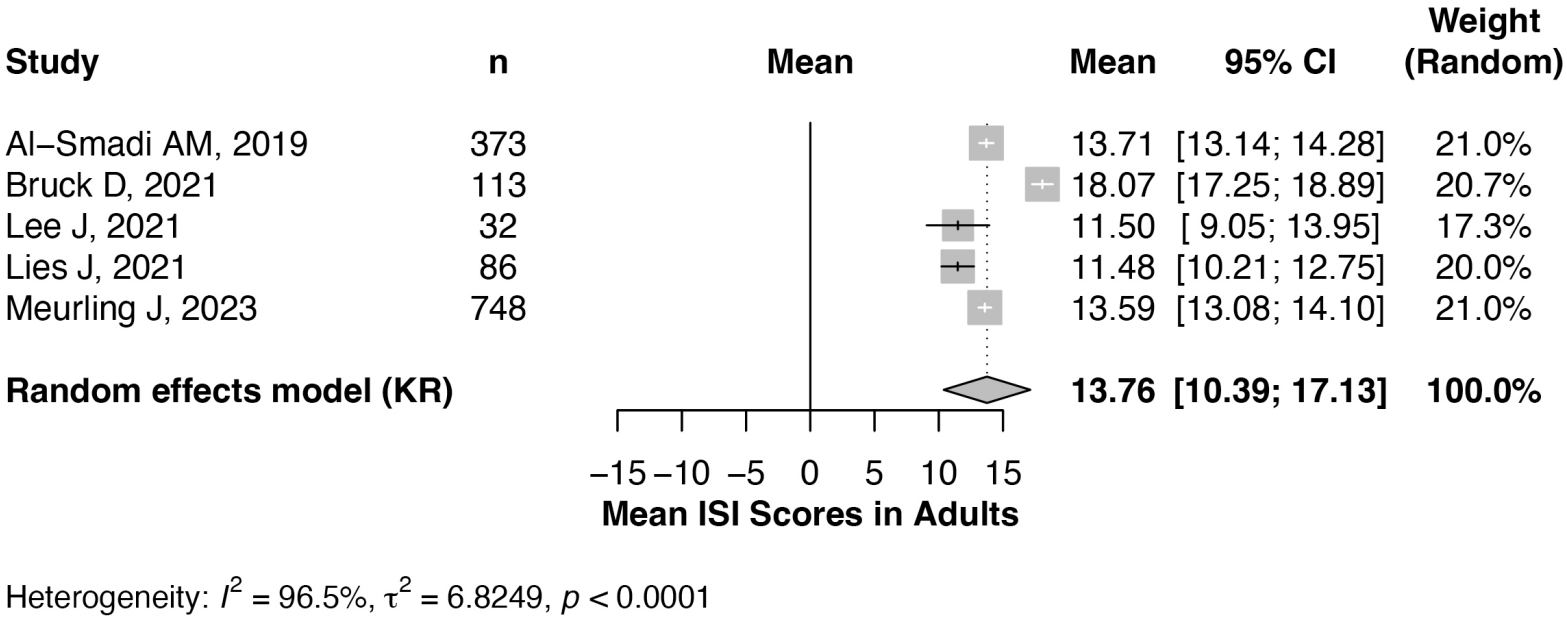
Forest plot of mean ISI scores.

Insomnia severity, assessed using the Insomnia Severity Index (ISI) across five studies (n = 1,352) [41,44–47], showed a pooled mean score of 13.76 (95% CI: 10.39–17.13), where higher scores indicate worse symptoms of insomnia. The pooled mean ISI score falls within the range of sub-clinical to moderate clinical insomnia, and all five studies reported mean ISI values above 11, suggesting clinically relevant sleep disturbance across samples. However, substantial heterogeneity was observed (I² = 96.5%, τ² = 6.8249, p < 0.0001), indicating considerable variability in ISI scores across populations (Fig 2).

**Fig 3:**
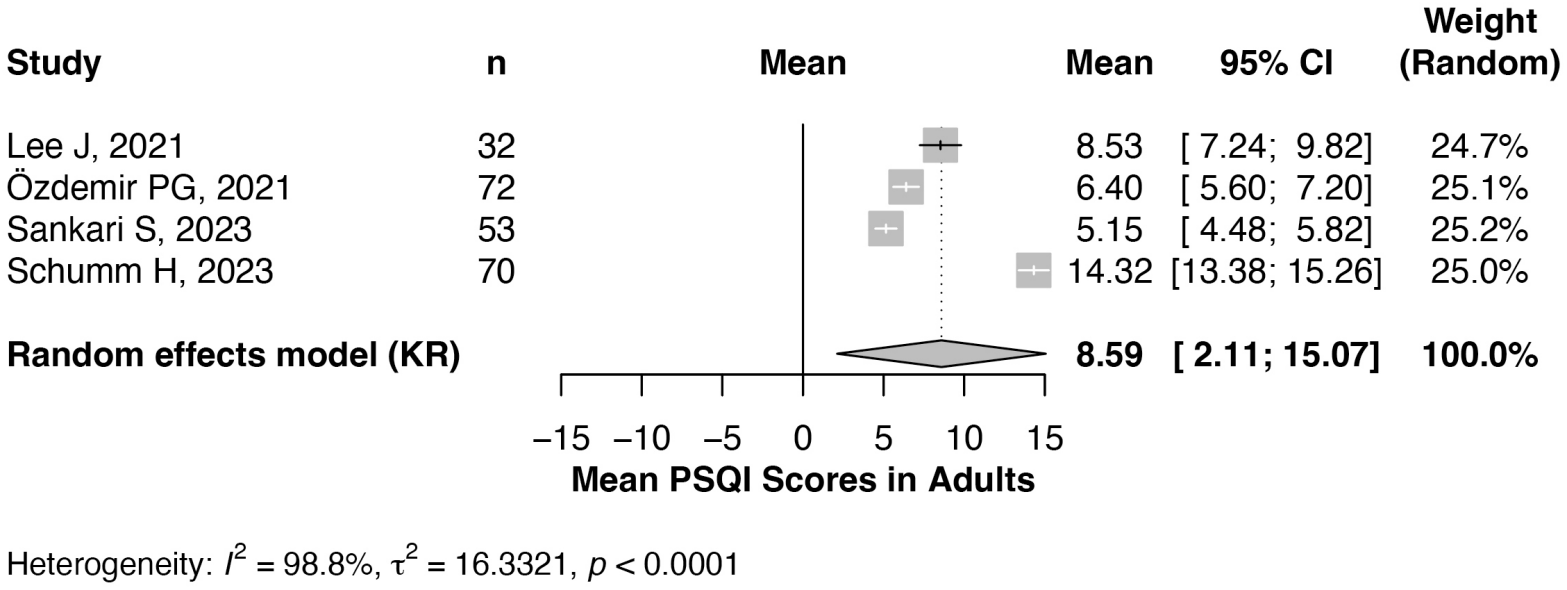
Forest plot of mean PSQI scores.

Similarly, sleep quality evaluated via the Pittsburgh Sleep Quality Index (PSQI) in four studies (n = 227) [38,40,41,43] demonstrated poor overall sleep quality (pooled mean = 8.59, 95% CI: 2.11–15.07). The pooled mean PSQI score exceeds the clinical cutoff of 5; however, the wide confidence interval and very high heterogeneity (I² = 98.8%, τ² = 16.3321, p < 0.0001) indicate substantial uncertainty and variability across studies (Fig 3).

### Secondary outcomes

**Fig 4:**
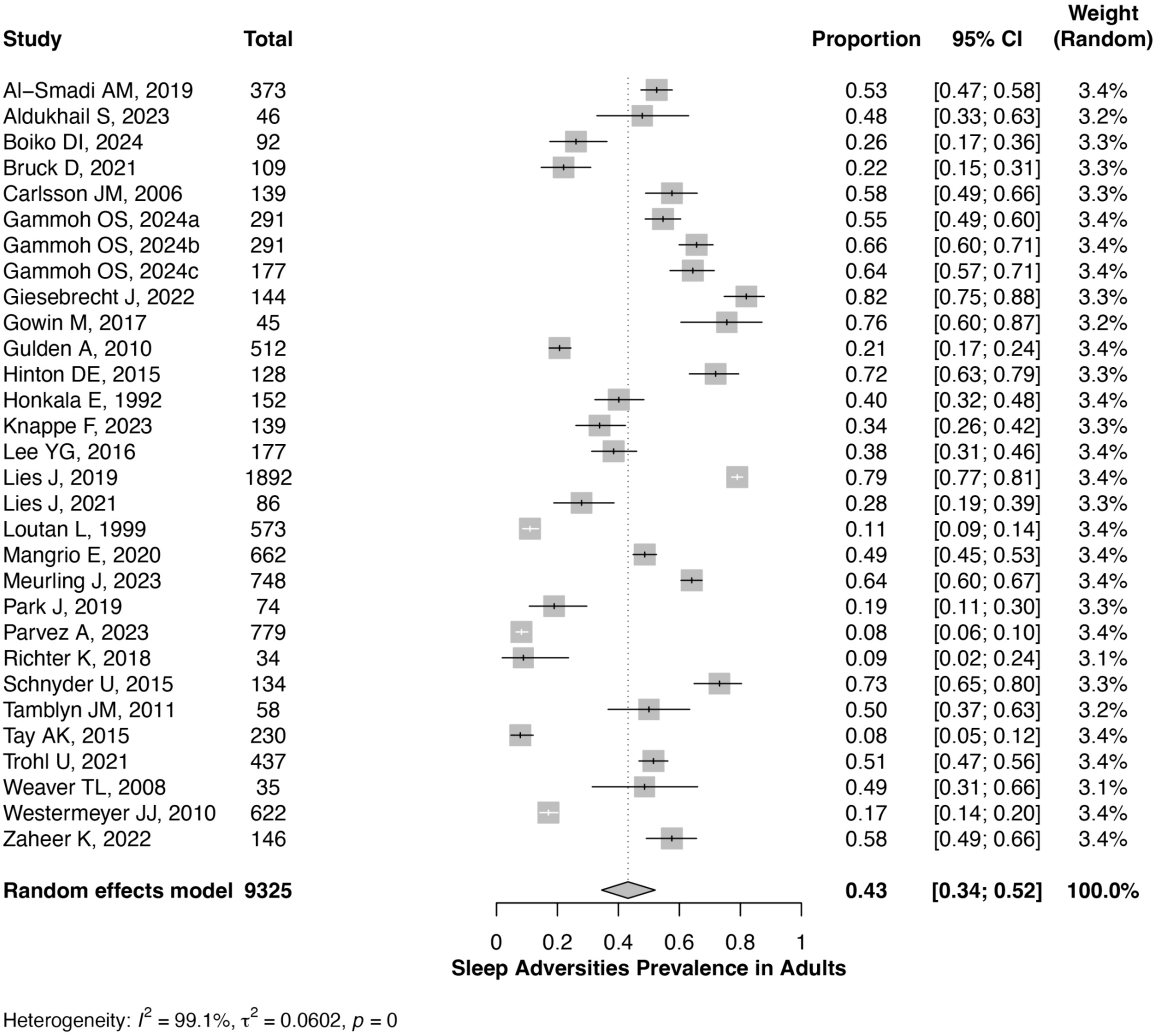
Forest plot of sleep adversities prevalence in adults.

**Fig 5:**
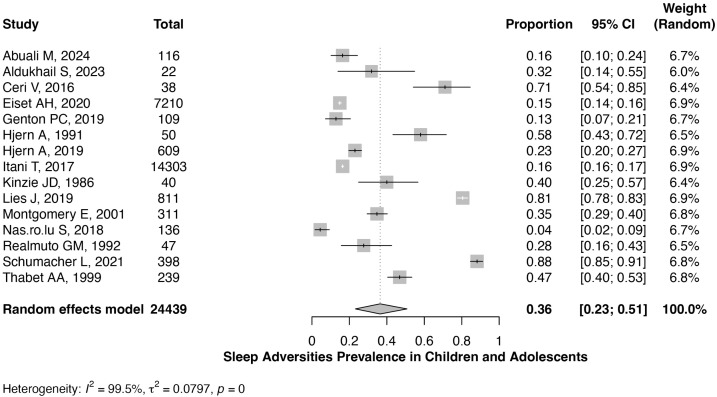
Forest plot of sleep adversities prevalence in children and adolescents.

We evaluated the prevalence of sleep adversities, an umbrella term for sleep problems including insomnia symptoms and nightmares (see S5Table for a full list of characteristics), in adults by analyzing data provided by 30 studies (n = 9,325), revealing an overall pooled prevalence of 43.2% (95% CI: 34.5–52.1%) through random-effects meta-analysis (Fig 4). This finding demonstrated very high heterogeneity (I² = 99.1%, τ² = 0.0602, p < 0.0001), indicating substantial between-study variability. Among children and adolescents (15 studies, n = 24,439), the pooled prevalence was 36.4% (95% CI: 23.2–50.8%), with similarly high heterogeneity (I² = 99.5%, τ² = 0.0797, p < 0.0001) (Fig 5).

Nightmare prevalence was specifically examined in six adult studies (n = 956) [51,56,83–85,87], showing a pooled estimate of 43.5% (95% CI: 21.6–66.8%) with significant between-study variation (I² = 97.9%, τ² = 0.0849, p < 0.0001). For children and adolescents (4 studies, n = 661) [73,74,88,100], the prevalence was 36.0% (95% CI: 11.9–64.8%), also with high heterogeneity (I² = 98.3%, τ² = 0.0863, p < 0.0001).

Sleep duration analysis revealed adults (3 studies, n = 231) [41,44,45] averaged 6**.**84 hours (95% CI: 5.84–7.85), while children and adolescents (2 studies, n = 229) showed longer duration at 8.45 hours (95% CI: 8.21–8.68). Sleep quality assessments, standardized to a 4-point Likert scale, indicated poorer quality in adults (8 studies, n = 6,353; pooled score = 2.30, 95% CI: 1.63–2.97) [53,64,65,68–72] compared to youth (2 studies, n = 459; pooled score = 2.10, 95% CI: −1.22–5.42) [75,90], although estimates in youth were imprecise and characterized by wide confidence intervals.

Finally, sleep latency measurements demonstrated adults (2 studies, n = 118) [41,45] had shorter onset times (20.56 minutes, 95% CI: 17.24–23.87) than children and adolescents (2 studies, n = 229; 58.13 minutes, 95% CI: −148.55–264.80) [88,90], with particularly wide confidence intervals in pediatric samples reflecting substantial statistical uncertainty.

### Influence analysis

Influence analysis identified Bruck D, 2021 [44] as the primary source of heterogeneity in the ISI scale meta-analysis (I² = 96.5% in primary analysis). After excluding this study (n = 1,239 remaining), the recalculated pooled ISI score decreased by 6.8% to 12.83 (95% CI: 10.38–15.28) from the original 13.76. While heterogeneity remained substantial (I² = 76.4%, τ² = 1.1112, p = 0.0053), this represents a 20.1% reduction in between-study variance (from I² = 96.5%). For all other outcomes (PSQI, prevalence measures, sleep duration/latency), influence analyses revealed no single study disproportionately contributing to heterogeneity, despite consistently high I² values. Complete sensitivity analyses are provided in the File in S1 File.

### Subgroup analysis

**Fig 6:**
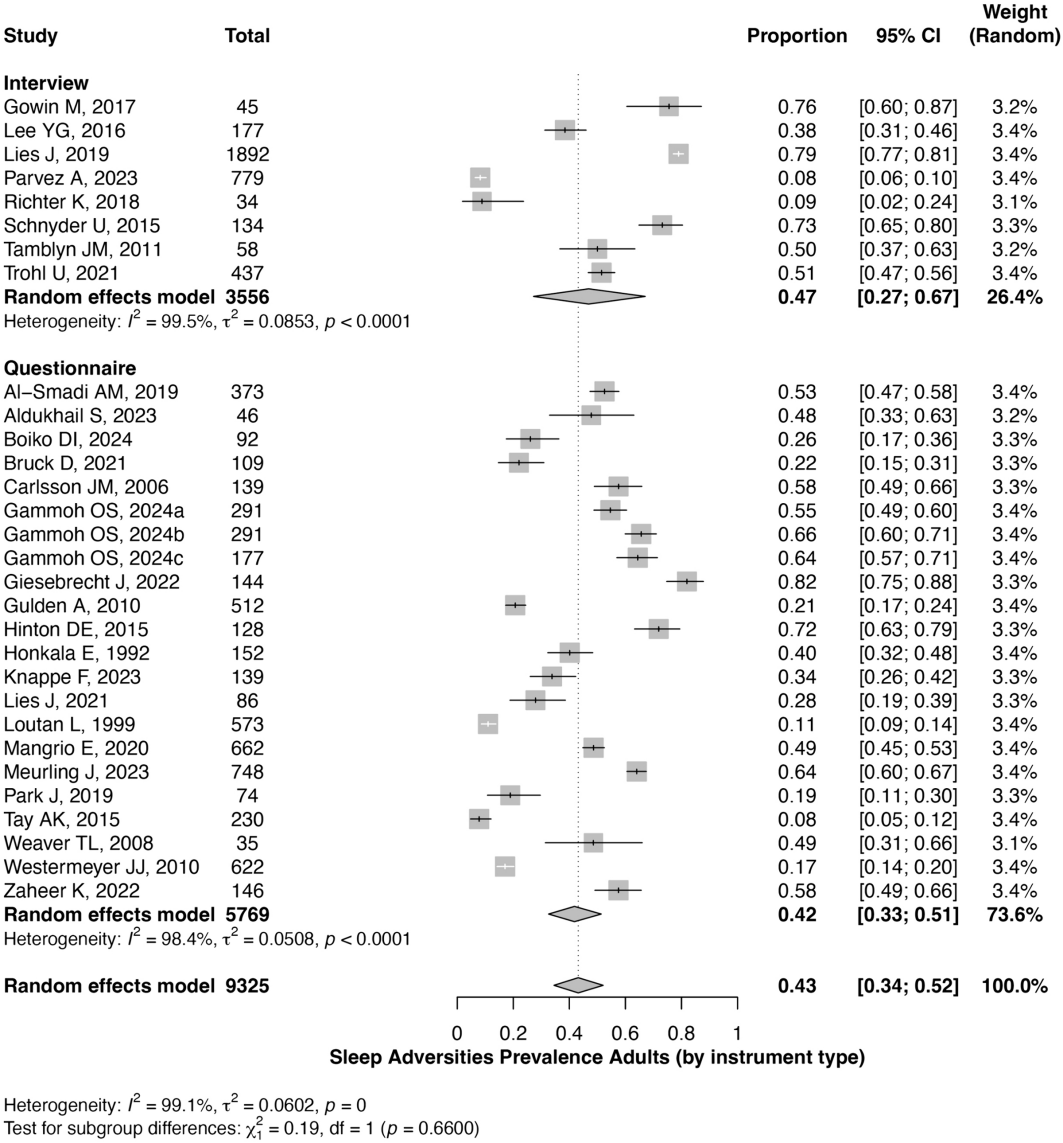
Forest plot of sleep adversities prevalence in adults by instrument type.

Our exploration of potential sources of heterogeneity identified predominantly non-significant subgroup differences across populations. Among adults, interview-based assessments yielded higher sleep adversities prevalence (46.9%, 95% CI: 27.2–67.1%, I² = 99.5%) than questionnaires (41.8%, 95% CI: 32.5–51.3%, I² = 98.4%) (Fig 6), with a similar 8–13% elevation among those with healthcare access (48.9% [95% CI: 37.2–60.7%], I² = 99.3% vs. without access 38.7% [95% CI: 26.1–52.2%], I² = 98.5%), although these differences were not statistically significant. Mixed-access populations showed the lowest prevalence (29.1%, 95% CI: 0.05–63.4%, I² = 96.9%), with wide confidence intervals indicating imprecision.

Study quality showed minimal impact on overall sleep adversities prevalence, with low quality studies reporting 46.3% (95% CI: 25.1–68.3%, I² = 99.1%), medium quality studies 42.2% (95% CI: 32.2–52.4%, I² = 99%), and high quality studies 47.0% (95% CI: 21.9–73.0%, I² = 97.5%). However, for nightmare prevalence specifically, medium quality studies detected substantially higher rates (66.0%, 95% CI: 44.4–84.6%, I² = 93.4%) than low quality studies (22.4%, 95% CI: 8.2–40.9%, I² = 96.7%), representing the only statistically significant subgroup difference observed in prevalence analyses. For continuous measures, the ISI revealed significant quality-related variation, with high quality studies reporting lower severity (11.48, 95% CI: 10.21–12.75) compared to medium quality (14.33, 95% CI: 9.93–18.72, I² = 96.9%).

**Fig 7:**
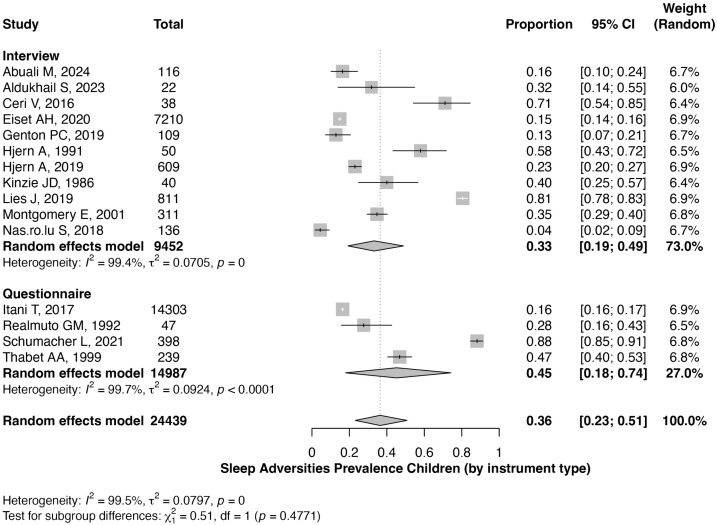
Forest plot of sleep adversities prevalence in children and adolescents by instrument type.

The stratification analysis on outcomes related to children and adolescents similarly demonstrated numerically higher prevalence estimates with questionnaires (45.2%, 95% CI: 17.9–74.2%, I² = 99.7%) compared to interviews (33.2%, 95% CI: 19.2–48.9%, I² = 99.4%) (Fig 7), although subgroup differences were not statistically significant. A comparable non-significant pattern was observed for healthcare access. Risk of bias analyses indicated lower prevalence in medium-quality (36.4%, 95% CI: 18.9–55.9%, I² = 99.6%) compared to low-quality studies (42.7%, 95% CI: 26.2–60.0%). For nightmare prevalence in children, medium-quality studies reported 30.3% (95% CI: 4.9–65.5%, I² = 98.9%) compared to 55.0% in low-quality studies (95% CI: 39.6–69.9%), without statistical significance.

Although some subgroup differences exceeded 5% in absolute prevalence, most comparisons did not reach statistical significance. Heterogeneity remained high throughout all analyses (I² ≥ 93.4%), indicating that measurement tool, healthcare access, and study quality explain only a limited proportion of the observed variability, with additional unmeasured moderators likely contributing to the substantial between-study differences.

### Meta-regressions

Our meta-regression analyses did not identify any statistically robust moderators capable of explaining the observed heterogeneity in prevalence estimates. Across both univariate and multivariable models, substantial residual heterogeneity persisted, and explained variance estimates were either minimal or appeared unstable in models with reduced sample size. Sensitivity analyses further indicated limited model stability, as parameter estimates were sensitive to the removal of individual studies. These findings remained consistent despite testing multiple theoretically relevant covariates, including assessment methodology, healthcare access, and geographic region. In light of these limitations and to avoid overinterpretation, we do not present detailed meta-regression coefficients in the main results. Complete modeling outputs, including all tested covariates and parameter estimates, are provided in S6 Table.

### Publication bias assessment

Visual inspection of funnel plots for sleep adversities prevalence in both age subgroups suggested potential publication bias and heterogeneity. However, Egger’s test revealed no statistically significant asymmetry for either adults or children/adolescents. While these results suggest no substantial evidence of small-study effects, the presence of very high heterogeneity warrants cautious interpretation.

Rücker’s limit meta-analysis provided additional assessment of potential small-study effects. In adults, the adjusted prevalence estimate (44.2%, 95% CI: 31.7–57.0%) closely matched the unadjusted estimate (43.2%, 95% CI: 34.5–52.1%). The adjustment left heterogeneity virtually unchanged (I² = 99.1%, τ² = 0.0602), indicating that small-study effects had minimal impact on the pooled estimate, while substantial between-study variability remained unexplained. For children and adolescents, the adjusted prevalence (34.3%, 95% CI: 17.3–53.8%) showed only a modest reduction from the unadjusted estimate (36.4%, 95% CI: 23.2–50.8%). Post-adjustment, the dataset maintained very high heterogeneity (I² = 99.5%, τ² = 0.0797). The substantial overlap between adjusted and unadjusted confidence intervals suggests that any potential small-study effects contributed only minimally to the overall pooled prevalence estimates.

### Studies that compared refugee and non-refugee populations

The studies demonstrated significant differences in sleep patterns between refugee and non-refugee populations across diverse contexts. Lee J et al. (2021) [41] found North Korean refugees exhibited distinct sleep architecture alterations, including shorter wake after sleep onset (WASO) and reduced N1 sleep compared to South Korean controls, and also identified a compensatory relationship between sleep continuity and attention in the refugee group. Bruck D et al. (2021) [44] reported substantially higher rates of subjective sleep complaints among South Sudanese refugees in Australia, with nearly two-thirds reporting inadequate sleep, more than double the Australian comparison group, and clinical insomnia rates six times higher among refugee men. Similarly, Lee YJ et al. (2016) [59] observed a greater prevalence of insomnia among North Korean refugees compared to South Koreans, with stronger associations to depression and PTSD symptoms. Mölsä M et al. (2014) [68] documented increased sleep difficulties among Somali refugees relative to Finnish natives, while Boiko DI et al. (2024) [98] found Ukrainian refugees exhibited approximately double the insomnia prevalence of non-refugees Ukrainians (26.1% vs 13.5%). However, both groups demonstrated war-related sleep disturbances, suggesting that although displacement exacerbates sleep problems, conflict exposure itself negatively affects sleep quality across affected populations.

### Narrative synthesis of studies not included in meta-analysis

Three studies reporting clinically relevant sleep measures were excluded from meta-analysis due to methodological heterogeneity but provided valuable supplementary findings. Among North Korean refugees, Lee S et al. (2021) [86] reported elevated nightmare distress (Mean NDQ score = 26.89, SD = 11.14), slightly exceeding the clinical cutoff [26]. Hinton et al. (2005) [96] documented striking sleep paralysis prevalence among Cambodian refugees: 42% overall and 67% in PTSD patients versus 22.4% in non-PTSD individuals. Similarly, Simich et al. (2006) [89] found 53.5% of Sudanese refugees in Canada endorsed “lost sleep over worry” as a primary distress symptom. Collectively, these findings reinforce the cross-cultural prevalence of sleep disturbances while highlighting condition-specific manifestations across refugee subgroups.

## Discussion

This systematic review and meta-analysis suggests that insomnia symptoms and impaired sleep quality are frequently reported among refugee and asylum-seeking populations. Across the included studies, mean scores on commonly used sleep instruments fell within ranges typically associated with clinically relevant sleep disturbance. At the same time, substantial variability was observed across studies, and between-study heterogeneity remained high. These findings therefore indicate a recurring pattern of sleep disruption in the populations studied, although the magnitude of the estimates should be interpreted cautiously given the methodological diversity and statistical heterogeneity observed.

When considered alongside evidence from general population samples, sleep disturbances reported in refugee populations appear to be more frequently reported than in community cohorts. A recent global meta-analysis estimated the prevalence of insomnia disorder at 13.9% (95% CI 10.9–17.6%) based on DSM criteria [103]. Large population-based studies from Europe and North America similarly report insomnia symptoms in approximately 7–23% of adults [104]. Estimates from Latin America also suggest lower prevalence of sleep disturbances in community samples compared with those observed in the present analysis [105]. Community validation studies have likewise reported mean Insomnia Severity Index scores within the subthreshold range in healthy populations [106], with established cut-off values distinguishing subclinical, moderate, and severe insomnia [107].

Sleep quality indicators from population-based cohorts also tend to be lower than those observed in the refugee samples included in this review. For example, a meta-analysis of studies conducted in China reported mean Pittsburgh Sleep Quality Index scores consistent with generally good sleep quality [108], while large European community samples have shown mean values near the clinical threshold for poor sleep quality [109]. In contrast, many studies included in the present review reported poorer subjective sleep quality and higher insomnia symptom scores. Although differences in study design, measurement tools, and cultural context complicate direct comparisons, the available evidence suggests that sleep disturbances may be more frequently reported among displaced populations than among general community samples.

Comparisons with other vulnerable populations exposed to conflict or displacement provide additional context. Studies conducted among internally displaced persons have documented considerable sleep disturbances, although prevalence estimates vary across settings [110,111]. In some contexts, symptom levels appear comparable to those reported among refugee samples, whereas in others they appear somewhat lower. By contrast, studies of populations experiencing socioeconomic hardship without exposure to armed conflict or forced displacement tend to report lower prevalence of sleep disturbances [112]. These patterns suggest that chronic insecurity, trauma exposure, and displacement-related stressors may contribute to sleep disruption, although causal relationships cannot be determined from the available data.

The broader literature on forced migration highlights the complex contexts in which refugee populations experience displacement and resettlement. Many studies included in this review focused on populations originating from regions heavily affected by armed conflict, particularly Western and Southern Asia and parts of Africa, reflecting global displacement patterns [113]. However, results were often not disaggregated by country of origin or cultural background, and several studies combined participants from multiple regions. This limits the ability to examine potential contextual or cultural differences in sleep outcomes across refugee groups. In addition, most available studies were conducted in high-income host countries, despite the majority of refugees residing in low- and middle-income settings. This imbalance may obscure important structural determinants of sleep health, including housing conditions, environmental safety, and access to healthcare services.

Sleep disturbances were also frequently reported among refugee children and adolescents. Although pooled estimates suggested somewhat lower prevalence compared with adults, substantial variability was observed across studies. Nightmares and other sleep-related symptoms were commonly reported among youth, consistent with clinical observations that trauma exposure and insecurity may manifest through sleep disruption and heightened physiological arousal [16]. However, interpretation of these findings remains limited by methodological variability across studies. Many investigations combined children and adolescents within the same analytic groups, used cross-sectional designs, or relied on heterogeneous measurement instruments, which may contribute to the wide range of estimates observed across studies.

Methodological variability across studies also represents an important consideration. Investigators used a wide range of sleep-related measures, reflecting evolving diagnostic frameworks and diverse research contexts. While validated instruments such as the ISI and PSQI were frequently employed, objective sleep assessments were rarely reported, with only one included study using polysomnography [41]. The reliance on self-report measures may therefore limit the ability to evaluate physiological aspects of sleep architecture that could be relevant in trauma-exposed populations [114].

Another important feature of the present analysis is the high level of heterogeneity observed across outcomes. Even after exploring potential moderators such as assessment methods, healthcare access, and study quality, substantial variability remained unexplained. Similar patterns have been reported in other prevalence meta-analyses and in studies examining refugee mental health outcomes [5,13,115,116]. The diversity of refugee experiences, including differences in pre-migration trauma exposure, post-migration stressors, cultural interpretations of sleep problems, and host-country environments, likely contributes to this variability. As observed in other large-scale sleep meta-analyses, residual heterogeneity often reflects differences in sampling strategies, source populations, and contextual factors that cannot be fully captured in aggregated analyses [104].

### Limitations of the study

Several limitations should therefore be considered when interpreting these findings. First, substantial between-study heterogeneity persisted despite sensitivity analyses and exploration of potential moderators. Second, key contextual variables, including time since resettlement, healthcare access, trauma characteristics, and social determinants, were inconsistently reported across studies, limiting deeper investigation of factors associated with sleep outcomes. Third, the predominance of cross-sectional study designs precludes conclusions regarding causal relationships or the temporal evolution of sleep disturbances following displacement. Fourth, the eligibility criteria required studies to meet predefined methodological and reporting standards, which led to the exclusion of some studies and may limit the generalizability of the findings to all refugee populations or settings. Finally, although publication bias was assessed using multiple approaches, residual small-study effects cannot be entirely excluded.

Despite these limitations, the findings highlight the importance of considering sleep health within refugee populations. Future research would benefit from longitudinal designs that follow individuals across different phases of displacement and resettlement. Greater use of standardized and culturally validated instruments, alongside objective sleep measures such as actigraphy or polysomnography when feasible, may improve comparability across studies. Consistent reporting of contextual variables, including trauma exposure, living conditions, and duration of displacement, would further facilitate a more comprehensive understanding of factors associated with sleep disturbances in displaced populations.

From a clinical and public health perspective, incorporating sleep assessment into refugee health care may help identify individuals experiencing persistent sleep disturbances. Preliminary evidence from group-based programs such as Sleep Training adapted for Refugees (STARS) suggests that sleep-focused interventions may be feasible and acceptable among displaced populations, although further research is needed to evaluate their effectiveness across diverse contexts [117,118].

Among children and adolescents, interventions may need to be integrated with family- and school-based approaches addressing trauma exposure and emotional regulation. Parent-focused education on sleep hygiene and emotional support may help mitigate the secondary effects of stress on child well-being [16]. At the same time, broader structural determinants, including housing stability, environmental safety, and access to education and healthcare, remain important influences on sleep health. Addressing these conditions may therefore be essential for improving sleep outcomes among displaced populations.

## Conclusions

In conclusion, the available evidence suggests that sleep disturbances are frequently reported among refugees and asylum seekers across diverse contexts. Although estimates vary substantially across studies and methodological limitations remain, the overall pattern indicates that sleep health may represent an important component of well-being in forcibly displaced populations. Further research incorporating longitudinal designs, standardized measurement, and contextual variables will be essential to clarify the mechanisms and trajectories of sleep disturbance and to inform effective interventions.

## Supporting information

S1 File Additional analysis. (DOCX)

S1 Table Search strategies. (DOCX)

S2 Table Newcastle-Ottawa Scale (NOS) for cohort studies. (DOCX)

S3 Table Newcastle-Ottawa Scales (NOS) adapted for cross-sectional studies. (DOCX)

S4 Table Moderators literature gap. (DOCX)

S5 Table Terminology and definitions. (DOCX)

S6 Table Meta-regression models. (DOCX)

S7 Table PRISMA Checklist. (DOCX)

## References

[1] United Nations General Assembly. Convention relating to the status of refugees. Geneva: UN; 1951. https://www.unhcr.org/about-unhcr/overview/1951-refugee-convention

[2] Saadi A, Al-Rousan T, AlHeresh R. Refugee mental health-an urgent call for research and action. JAMA Netw Open. 2021;4(3):e212543. doi: 10.1001/jamanetworkopen.2021.2543 33724386 PMC8344400

[3] United Nations High Commissioner for Refugees. Figures at a glance. Geneva: UNHCR; 2026. https://www.unhcr.org/about-unhcr/overview/figures-glance

[4] United Nations High Commissioner for Refugees. Who we protect: asylum seekers. UNHCR; 2025. https://www.unhcr.org/about-unhcr/who-we-protect/asylum-seekers

[5] Mesa-Vieira C, Haas AD, Buitrago-Garcia D, Roa-Diaz ZM, Minder B, Gamba M, et al. Mental health of migrants with pre-migration exposure to armed conflict: a systematic review and meta-analysis. Lancet Public Health. 2022;7(5):e469–81. doi: 10.1016/S2468-2667(22)00061-5 35487232

[6] Daynes L. The health impacts of the refugee crisis: a medical charity perspective. Clin Med (Lond). 2016;16(5):437–40. doi: 10.7861/clinmedicine.16-5-437 27697805 PMC6297302

[7] Al-Rousan T, AlHeresh R, Saadi A, El-Sabrout H, Young M, Benmarhnia T, et al. Epidemiology of cardiovascular disease and its risk factors among refugees and asylum seekers: systematic review and meta-analysis. Int J Cardiol Cardiovasc Risk Prev. 2022;12:200126. doi: 10.1016/j.ijcrp.2022.200126 35199106 PMC8851152

[8] Carnethon MR, Johnson DA. Sleep and resistant hypertension. Curr Hypertens Rep. 2019;21(5):34. doi: 10.1007/s11906-019-0941-z 30953264 PMC7265173

[9] Mohr D, Vedantham K, Neylan T, Metzler TJ, Best S, Marmar CR. The mediating effects of sleep in the relationship between traumatic stress and health symptoms in urban police officers. Psychosom Med. 2003;65(3):485–9. doi: 10.1097/01.psy.0000041404.96597.38 12764223

[10] Clum GA, Nishith P, Resick PA. Trauma-related sleep disturbance and self-reported physical health symptoms in treatment-seeking female rape victims. J Nerv Ment Dis. 2001;189(9):618–22. doi: 10.1097/00005053-200109000-00008 11580006 PMC2970918

[11] Baglioni C, Nanovska S, Regen W, Spiegelhalder K, Feige B, Nissen C, et al. Sleep and mental disorders: A meta-analysis of polysomnographic research. Psychol Bull. 2016;142(9):969–90. doi: 10.1037/bul0000053 27416139 PMC5110386

[12] Hollifield M, Warner TD, Lian N, Krakow B, Jenkins JH, Kesler J, et al. Measuring trauma and health status in refugees: a critical review. JAMA. 2002;288(5):611–21. doi: 10.1001/jama.288.5.611 12150673

[13] Patanè M, Ghane S, Karyotaki E, Cuijpers P, Schoonmade L, Tarsitani L, et al. Prevalence of mental disorders in refugees and asylum seekers: a systematic review and meta-analysis. Glob Ment Health (Camb). 2022;9:250–63. doi: 10.1017/gmh.2022.29 36618716 PMC9806970

[14] Fazel M, Wheeler J, Danesh J. Prevalence of serious mental disorder in 7000 refugees resettled in western countries: a systematic review. Lancet. 2005;365(9467):1309–14. doi: 10.1016/S0140-6736(05)61027-6 15823380

[15] Richter K, Baumgärtner L, Niklewski G, Peter L, Köck M, Kellner S, et al. Sleep disorders in migrants and refugees: a systematic review with implications for personalized medical approach. EPMA J. 2020;11(2):251–60. doi: 10.1007/s13167-020-00205-2 32549917 PMC7272531

[16] Baskaran A, Marogi E, Bitar R, Attarian H, Saadi A. Improving sleep health among refugees: a systematic review. Neurol Clin Pract. 2023;13(2):e200139. doi: 10.1212/CPJ.0000000000200139 36936393 PMC10022726

[17] Buysse DJ, Reynolds 3rd CF, Monk TH, Berman SR, Kupfer DJ. The Pittsburgh Sleep Quality Index: a new instrument for psychiatric practice and research. Psychiatry Res. 1989;28(2):193–213. doi: 10.1016/0165-1781(89)90047-4 2748771

[18] Bastien CH, Vallières A, Morin CM. Validation of the Insomnia Severity Index as an outcome measure for insomnia research. Sleep Med. 2001;2(4):297–307. doi: 10.1016/s1389-9457(00)00065-4 11438246

[19] Liberati A, Altman DG, Tetzlaff J, Mulrow C, Gøtzsche PC, Ioannidis JPA, et al. The PRISMA statement for reporting systematic reviews and meta-analyses of studies that evaluate health care interventions: explanation and elaboration. PLoS Med. 2009;6(7):e1000100. doi: 10.1371/journal.pmed.1000100 19621070 PMC2707010

[20] Page MJ, McKenzie JE, Bossuyt PM, Boutron I, Hoffmann TC, Mulrow CD, et al. The PRISMA 2020 statement: an updated guideline for reporting systematic reviews. BMJ. 2021;372:n71. doi: 10.1136/bmj.n71 33782057 PMC8005924

[21] Stroup DF, Berlin JA, Morton SC, Olkin I, Williamson GD, Rennie D. Meta-analysis of observational studies in epidemiology: a proposal for reporting. JAMA. 2000;283(15):2008–12. doi: 10.1001/jama.283.15.2008 10789670

[22] Linden M, Muschalla B. Standardized diagnostic interviews, criteria, and algorithms for mental disorders: garbage in, garbage out. Eur Arch Psychiatry Clin Neurosci. 2012;262(6):535–44. doi: 10.1007/s00406-012-0293-z 22274737

[23] Kellermeyer L, Harnke B, Knight S. Covidence and Rayyan. J Med Libr Assoc. 2018;106(4):580–3. doi: 10.5195/jmla.2018.513

[24] Sterne JAC, Savović J, Page MJ, Elbers RG, Blencowe NS, Boutron I, et al. RoB 2: a revised tool for assessing risk of bias in randomised trials. BMJ. 2019;366:l4898. doi: 10.1136/bmj.l4898 31462531

[25] Wells GA, Shea B, O’Connell D, Peterson J, Welch V, Losos M. The Newcastle-Ottawa Scale (NOS) for assessing the quality of nonrandomized studies in meta-analyses. Ottawa: Ottawa Hospital Research Institute; 2009. http://www.ohri.ca/programs/clinical_epidemiology/oxford.htm

[26] Herzog R, Álvarez-Pasquin MJ, Díaz C, Del Barrio JL, Estrada JM, Gil Á. Are healthcare workers’ intentions to vaccinate related to their knowledge, beliefs and attitudes? A systematic review. BMC Public Health. 2013;13:154. doi: 10.1186/1471-2458-13-154 23421987 PMC3602084

[27] Borenstein M, Higgins JPT, Hedges LV, Rothstein HR. Basics of meta-analysis: I2 is not an absolute measure of heterogeneity. Res Synth Methods. 2017;8(1):5–18. doi: 10.1002/jrsm.1230 28058794

[28] Higgins JPT, Thomas J, Chandler J, Cumpston M, Li T, Page MJ. Cochrane Handbook for Systematic Reviews of Interventions. London: Cochrane; 2024.

[29] Harrer M, Cuijpers P, Furukawa TA, Ebert DD. Doing meta-analysis with R: a hands-on guide. Boca Raton (FL): Chapman & Hall/CRC Press; 2021.

[30] Cinar O, Umbanhowar J, Hoeksema JD, Viechtbauer W. Using information-theoretic approaches for model selection in meta-analysis. Res Synth Methods. 2021;12(4):537–56. doi: 10.1002/jrsm.1489 33932323 PMC8359854

[31] van Buuren S, Groothuis-Oudshoorn K. Mice: Multivariate imputation by chained equations in R. J Stat Softw. 2011;45(3):1–67. doi: 10.18637/jss.v045.i03

[32] Viechtbauer W. Conducting meta-analyses in R with the metafor package. J Stat Softw. 2010;36(3):1–48. doi: 10.18637/jss.v036.i03

[33] Bartoń K. MuMIn: multi-model inference. Vienna: R Foundation; 2020.

[34] Sterne JAC, Sutton AJ, Ioannidis JPA, Terrin N, Jones DR, Lau J, et al. Recommendations for examining and interpreting funnel plot asymmetry in meta-analyses of randomised controlled trials. BMJ. 2011;343:d4002. doi: 10.1136/bmj.d4002 21784880

[35] Rücker G, Schwarzer G, Carpenter JR, Binder H, Schumacher M. Treatment-effect estimates adjusted for small-study effects via a limit meta-analysis. Biostatistics. 2011;12(1):122–42. doi: 10.1093/biostatistics/kxq046 20656692

[36] Peters JL, Sutton AJ, Jones DR, Abrams KR, Rushton L. Performance of the trim and fill method in the presence of publication bias and between-study heterogeneity. Stat Med. 2007;26(25):4544–62. doi: 10.1002/sim.2889 17476644

[37] Spanhel K, Hovestadt E, Lehr D, Spiegelhalder K, Baumeister H, Bengel J, et al. Engaging refugees with a culturally adapted digital intervention to improve sleep: a randomized controlled pilot trial. Front Psychiatry. 2022;13:832196. doi: 10.3389/fpsyt.2022.832196 35280163 PMC8905517

[38] Sankari S, Wrobel N, Leonard M, Grasser L, Sankari A, Javanbakht A. Relationship between posttraumatic stress disorder and sleep disturbances in Syrian Refugees in the United States. Avicenna J Med. 2023;13(2):82–8. doi: 10.1055/s-0043-1768646 37435556 PMC10332942

[39] Richter K, Peter L, Lehfeld H, Zäske H, Brar-Reissinger S, Niklewski G. Prevalence of psychiatric diagnoses in asylum seekers with follow-up. BMC Psychiatry. 2018;18(1):206. doi: 10.1186/s12888-018-1783-y 29925338 PMC6011353

[40] Ozdemir PG, Kirli U, Asoglu M. Investigation of the associations between posttraumatic growth, sleep quality and depression symptoms in Syrian refugees. East J Med. 2021;26(2):265–72. doi: 10.5505/ejm.2021.48108

[41] Lee J, Jeon S, Kim S, Seo Y, Park J, Lee YJ, et al. Polysomnographic Sleep and Attentional Deficits in Traumatized North Korean Refugees. Nat Sci Sleep. 2021;13:635–45. doi: 10.2147/NSS.S308968 34079408 PMC8163968

[42] Sandahl H, Jennum P, Baandrup L, Lykke Mortensen E, Carlsson J. Imagery rehearsal therapy and/or mianserin in treatment of refugees diagnosed with PTSD: Results from a randomized controlled trial. J Sleep Res. 2021;30(4):e13276. doi: 10.1111/jsr.13276 33529449 PMC8365672

[43] Schumm H, Steil R, Lechner-Meichsner F, Morina N, Weise C, Mewes R, et al. Associations between sleep problems and posttraumatic stress symptoms, social functioning, and quality of life in refugees with posttraumatic stress disorder. J Trauma Stress. 2023;36(6):1176–83. doi: 10.1002/jts.22983 37883129

[44] Bruck D, Atem Deng S, Kot B, Grossman M. Sleep difficulties among South Sudanese former refugees settled in Australia. Transcult Psychiatry. 2021;58(2):172–86. doi: 10.1177/1363461520903122 32216546

[45] Lies J, Jobson L, Mascaro L, Whyman T, Drummond SPA. Postmigration stress and sleep disturbances mediate the relationship between trauma exposure and posttraumatic stress symptoms among Syrian and Iraqi refugees. J Clin Sleep Med. 2021;17(3):479–89. doi: 10.5664/jcsm.8972 33141012 PMC7927347

[46] Al-Smadi AM, Tawalbeh LI, Gammoh OS, Ashour A, Tayfur M, Attarian H. The prevalence and the predictors of insomnia among refugees. J Health Psychol. 2019;24(8):1125–33. doi: 10.1177/1359105316687631 28810381

[47] Meurling J, Rondung E, Leiler A, Wasteson E, Andersson G, Richards D, et al. An online tiered screening procedure to identify mental health problems among refugees. BMC Psychiatry. 2023;23(1):7. doi: 10.1186/s12888-022-04481-2 36597066 PMC9811744

[48] Park J, Elbert T, Kim SJ, Park J. The contribution of posttraumatic stress disorder and depression to insomnia in North Korean Refugee Youth. Front Psychiatry. 2019;10:211. doi: 10.3389/fpsyt.2019.00211 31024363 PMC6463899

[49] Gammoh O, Aljabali AAA, Tambuwala MM. The crosstalk between subjective fibromyalgia, mental health symptoms and the use of over-the-counter analgesics in female Syrian refugees: a cross-sectional web-based study. Rheumatol Int. 2024;44(4):715–23. doi: 10.1007/s00296-023-05521-0 38285107 PMC10914905

[50] Lies J, Mellor A, Jobson L, Drummond SPA. Prevalence of sleep disturbance and its relationships with mental health and psychosocial issues in refugees and asylum seekers attending psychological services in Australia. Sleep Health. 2019;5(4):335–43. doi: 10.1016/j.sleh.2019.06.002 31320291

[51] Carlsson JM, Olsen DR, Mortensen EL, Kastrup M. Mental health and health-related quality of life: a 10-year follow-up of tortured refugees. J Nerv Ment Dis. 2006;194(10):725–31. doi: 10.1097/01.nmd.0000243079.52138.b7 17041283

[52] Aldukhail S, Shukla A, Khadra MT, Al Hennawi Z, Jordan S, Cadet TJ, et al. Oral and emotional health experience of refugees’ in the state of Massachusetts - a mixed methods approach. PLoS One. 2023;18(3):e0281361. doi: 10.1371/journal.pone.0281361 36893206 PMC9997929

[53] Rizzi D, Ciuffo G, Sandoli G, Mangiagalli M, de Angelis P, Scavuzzo G, et al. Running away from the war in Ukraine: The Impact on Mental Health of Internally Displaced Persons (IDPs) and Refugees in Transit in Poland. Int J Environ Res Public Health. 2022;19(24):16439. doi: 10.3390/ijerph192416439 36554321 PMC9778520

[54] Parvez A, Percac-Lima S, Saadi A. The Presence and Profile of Neurological Conditions and Associated Psychiatric Comorbidities in U.S. Resettled Refugees: A Retrospective Single Center Study. J Immigr Minor Health. 2023;25(2):365–73. doi: 10.1007/s10903-022-01409-6 36251204

[55] Trohl U, Wagner K, Kalfa V, Negash S, Wienke A, Führer A. Sick and Tired-Sociodemographic and Psychosocial Characteristics of Asylum Seekers Awaiting an Appointment for Psychotherapy. Int J Environ Res Public Health. 2021;18(22):11850. doi: 10.3390/ijerph182211850 34831606 PMC8619663

[56] Tay AK, Rees S, Chan J, Kareth M, Silove D. Examining the broader psychosocial effects of mass conflict on PTSD symptoms and functional impairment amongst West Papuan refugees resettled in Papua New Guinea (PNG). Soc Sci Med. 2015;132:70–8. doi: 10.1016/j.socscimed.2015.03.020 25795990

[57] Tamblyn JM, Calderon AJ, Combs S, O’Brien MM. Patients from abroad becoming patients in everyday practice: torture survivors in primary care. J Immigr Minor Health. 2011;13(4):798–801. doi: 10.1007/s10903-010-9429-2 21188531

[58] Loutan L, Bollini P, Pampallona S, Bierens de Haan D, Gariazzo F. Impact of trauma and torture on asylum-seekers. Eur J Public Health. 1999;9(2):93–6. doi: 10.1093/eurpub/9.2.93

[59] Lee Y-JG, Jun JY, Lee YJ, Park J, Kim S, Lee SH, et al. Insomnia in North Korean Refugees: Association with Depression and Post-Traumatic Stress Symptoms. Psychiatry Investig. 2016;13(1):67–73. doi: 10.4306/pi.2016.13.1.67 26766948 PMC4701687

[60] Honkala E, Maidi D, Kolmakow S. Dental caries and stress among South African political refugees. Quintessence Int. 1992;23(8):579–83. 1410264

[61] Westermeyer JJ, Campbell R, Lien R, Spring M, Johnson DR, Butcher J, et al. HADStress: a somatic symptom screen for posttraumatic stress among Somali refugees. Psychiatr Serv. 2010;61(11):1132–7. doi: 10.1176/ps.2010.61.11.1132 21041353

[62] Gulden A, Westermeyer J, Lien R, Spring M, Johnson D, Butcher J, et al. HADStress screen for posttraumatic stress: replication in ethiopian refugees. J Nerv Ment Dis. 2010;198(10):762–7. doi: 10.1097/NMD.0b013e3181f49c0a 20921868

[63] Gowin M, Taylor EL, Dunnington J, Alshuwaiyer G, Cheney MK. Needs of a silent minority: Mexican transgender asylum seekers. Health Promot Pract. 2017;18(3):332–40. doi: 10.1177/1524839917692750 28187690

[64] Schlechter P, Hellmann JH, Morina N. Unraveling specifics of mental health symptoms in war survivors who fled versus stayed in the area of conflict using network analysis. J Affect Disord. 2021;290:93–101. doi: 10.1016/j.jad.2021.04.072 33993086

[65] Mootoo C, Fountain C, Rasmussen A. Formative psychosocial evaluation using dynamic networks: trauma, stressors, and distress among Darfur refugees living in Chad. Confl Health. 2019;13:30. doi: 10.1186/s13031-019-0212-2 31391864 PMC6595582

[66] Schnyder U, Müller J, Morina N, Schick M, Bryant RA, Nickerson A. A comparison of DSM-5 and DSM-IV diagnostic criteria for posttraumatic stress disorder in traumatized refugees. J Trauma Stress. 2015;28(4):267–74. doi: 10.1002/jts.22023 26194738

[67] Weaver TL, Cajdrić A, Jackson ER. Smoking patterns within a primary care sample of resettled Bosnian refugees. J Immigr Minor Health. 2008;10(5):407–14. doi: 10.1007/s10903-007-9102-6 18071902

[68] Mölsä M, Punamäki R-L, Saarni SI, Tiilikainen M, Kuittinen S, Honkasalo M-L. Mental and somatic health and pre- and post-migration factors among older Somali refugees in Finland. Transcult Psychiatry. 2014;51(4):499–525. doi: 10.1177/1363461514526630 24648488

[69] Vinson GA, Chang Z. PTSD symptom structure among West African war trauma survivors living in African refugee camps: a factor-analytic investigation. J Trauma Stress. 2012;25(2):226–31. doi: 10.1002/jts.21681 22522740

[70] Zaheer K, Wanyonyi K, Williams DM. Oral health-related quality of life of refugees in settlements in Greece. Int Dent J. 2022;72(5):706–15. doi: 10.1016/j.identj.2022.04.004 35570016 PMC9485531

[71] Lindheimer N, Karnouk C, Hahn E, Churbaji D, Schilz L, Rayes D, et al. Exploring the representation of depressive symptoms and the influence of stigma in Arabic-Speaking refugee outpatients. Front Psychiatry. 2020;11:579057. doi: 10.3389/fpsyt.2020.579057 33281643 PMC7689084

[72] Giesebrecht J, Grupp F, Reich H, Weise C, Mewes R. Relations between criteria for somatic symptom disorder and quality of life in asylum seekers living in Germany. J Psychosom Res. 2022;160:110977. doi: 10.1016/j.jpsychores.2022.110977 35803108

[73] Abuali M, Navarro I, Baisch M, Bashkenova N, Chang-Escobar S, Paoletti A, et al. Health profile of Afghan pediatric refugees resettled to Philadelphia in 2021-2022. Clin Pediatr (Phila). 2024;63(2):222–5. doi: 10.1177/00099228231208611 37905725

[74] Montgomery E, Foldspang A. Traumatic experience and sleep disturbance in refugee children from the Middle East. Eur J Public Health. 2001;11(1):18–22. doi: 10.1093/eurpub/11.1.18 11276566

[75] Pfeiffer E, Sukale T, Müller LRF, Plener PL, Rosner R, Fegert JM, et al. The symptom representation of posttraumatic stress disorder in a sample of unaccompanied and accompanied refugee minors in Germany: a network analysis. Eur J Psychotraumatol. 2019;10(1):1675990. doi: 10.1080/20008198.2019.1675990 31681465 PMC6807914

[76] Genton PC, Wang J, Bodenmann P, Ambresin A-E. Clinical profile and care pathways among unaccompanied minor asylum seekers in Vaud, Switzerland. Int J Adolesc Med Health. 2019;34(3):10.1515/ijamh-2019–0140. doi: 10.1515/ijamh-2019-0140 32229662

[77] Ceri V, Özlü-Erkilic Z, Özer Ü, Yalcin M, Popow C, Akkaya-Kalayci T. Psychiatric symptoms and disorders among Yazidi children and adolescents immediately after forced migration following ISIS attacks. Neuropsychiatr. 2016;30(3):145–50. doi: 10.1007/s40211-016-0195-9 27628299 PMC5063909

[78] Eiset AH, Loua AS, Kruse A, Norredam M. The health status of newly arrived asylum-seeking minors in Denmark: a nationwide register-based study. Int J Public Health. 2020;65(9):1763–72. doi: 10.1007/s00038-020-01501-4 33084920

[79] Schumacher L, Burger J, Zoellner F, Zindler A, Epskamp S, Barthel D. Using clinical expertise and empirical data in constructing networks of trauma symptoms in refugee youth. Eur J Psychotraumatol. 2021;12(1):1920200. doi: 10.1080/20008198.2021.1920200 34178294 PMC8205066

[80] Hjern A, Kling S. Health care needs in school-age refugee children. Int J Environ Res Public Health. 2019;16(21):4255. doi: 10.3390/ijerph16214255 31683963 PMC6862330

[81] Hjern A, Angel B, Höjer B. Persecution and behavior: a report of refugee children from Chile. Child Abuse Negl. 1991;15(3):239–48. doi: 10.1016/0145-2134(91)90068-o 2043975

[82] Nasıroğlu S, Çeri V, Erkorkmaz Ü, Semerci B. Determinants of psychiatric disorders in children refugees in Turkey’s Yazidi refugee camp. Psychiatry and Clinical Psychopharmacology. 2018;28(3):291–9. doi: 10.1080/24750573.2017.1422958

[83] Husni M, Cernovsky ZZ, Koye N, Haggarty J. Nightmares of refugees from Kurdistan. J Nerv Ment Dis. 2001;189(8):557–8. doi: 10.1097/00005053-200108000-00010 11531209

[84] Hinton DE, Hinton AL, Pich V, Loeum JR, Pollack MH. Nightmares among Cambodian refugees: the breaching of concentric ontological security. Cult Med Psychiatry. 2009;33(2):219–65. doi: 10.1007/s11013-009-9131-9 19333741

[85] Cernovsky Z. Refugees’ repetitive nightmares. J Clin Psychol. 1988;44(5):702–7. doi: 10.1002/1097-4679(198809)44:5<702::aid-jclp2270440506>3.0.co;2-d 3192707

[86] Lee S, Lee J, Jeon S, Kim S, Seo Y, Park J, et al. Nightmares and alexithymia in traumatized North Korean refugees. Sleep Med. 2021;86:75–80. doi: 10.1016/j.sleep.2021.08.005 34464881

[87] Berkson SY, Tor S, Mollica R, Lavelle J, Cosenza C. An innovative model of culturally tailored health promotion groups for Cambodian survivors of torture. Torture. 2014;24(1):1–16. doi: 10.7146/torture.v24i1.109698 25047082

[88] Bronstein I, Montgomery P. Sleeping patterns of Afghan unaccompanied asylum-seeking adolescents: a large observational study. PLoS One. 2013;8(2):e56156. doi: 10.1371/journal.pone.0056156 23457517 PMC3573060

[89] Simich L, Hamilton H, Baya BK. Mental distress, economic hardship and expectations of life in Canada among Sudanese newcomers. Transcult Psychiatry. 2006;43(3):418–44. doi: 10.1177/1363461506066985 17090626

[90] Müller LRF, Gossmann K, Schmid RF, Rosner R, Unterhitzenberger J. A pilot study on ecological momentary assessment in asylum-seeking children and adolescents resettled to Germany: Investigating compliance, post-migration factors, and the relation between daily mood, sleep patterns, and mental health. PLoS One. 2021;16(2):e0246069. doi: 10.1371/journal.pone.0246069 33524043 PMC7850498

[91] Mangrio E, Zdravkovic S, Sjögren Forss K. The association between self-perceived health and sleep-quality and anxiety among newly arrived refugees in Sweden: a quantitative study. J Immigr Minor Health. 2020;22(1):82–6. doi: 10.1007/s10903-019-00871-z 30788680 PMC6952325

[92] Ku S-Y, Kang JW, Kim H, Kim YD, Jee BC, Suh CS, et al. Age at menarche and its influencing factors in North Korean female refugees. Hum Reprod. 2006;21(3):833–6. doi: 10.1093/humrep/dei271 16199433

[93] Knappe F, Filippou K, Hatzigeorgiadis A, Morres ID, Tzormpatzakis E, Havas E, et al. Psychological well-being, mental distress, metabolic syndrome, and associated factors among people living in a refugee camp in Greece: a cross-sectional study. Front Public Health. 2023;11:1179756. doi: 10.3389/fpubh.2023.1179756 37397726 PMC10311549

[94] Itani T, Jacobsen KH, Kraemer A. Suicidal ideation and planning among Palestinian middle school students living in Gaza Strip, West Bank, and United Nations Relief and Works Agency (UNRWA) camps. Int J Pediatr Adolesc Med. 2017;4(2):54–60. doi: 10.1016/j.ijpam.2017.03.003 30805502 PMC6372497

[95] Hinton DE, Reis R, de Jong J. The “Thinking a Lot” Idiom of Distress and PTSD: an examination of their relationship among traumatized Cambodian refugees using the “Thinking a Lot” questionnaire. Med Anthropol Q. 2015;29(3):357–80. doi: 10.1111/maq.12204 25772670

[96] Hinton DE, Pich V, Chhean D, Pollack MH. “The ghost pushes you down”: sleep paralysis-type panic attacks in a Khmer refugee population. Transcult Psychiatry. 2005;42(1):46–77. doi: 10.1177/1363461505050710 15881268

[97] Gammoh O, Durand H, Alqudah A, Qnais E, Ajlouni Y, Sakher SB, et al. Menstrual pain self-medication relates to poor mental health outcomes from Al-Zaatri refugees’ camp. Afr J Reprod Health. 2024;28(6):66–74. doi: 10.29063/ajrh2024/v28i6.7 38979874

[98] Boiko DI, Shyrai PO, Mats OV, Karpik ZI, Rahman MH, Khan AA, et al. Mental health and sleep disturbances among Ukrainian refugees in the context of Russian-Ukrainian war: A preliminary result from online-survey. Sleep Med. 2024;113:342–8. doi: 10.1016/j.sleep.2023.12.004 38104463

[99] Thabet AA, Vostanis P. Post-traumatic stress reactions in children of war. J Child Psychol Psychiatry. 1999;40(3):385–91. doi: 10.1017/s0021963098003709 10190340

[100] Kinzie JD, Sack WH, Angell RH, Manson S, Rath B. The psychiatric effects of massive trauma on Cambodian children: I. The Children. Journal of the American Academy of Child Psychiatry. 1986;25(3):370–6. doi: 10.1016/s0002-7138(09)60259-4

[101] Realmuto GM, Masten A, Carole LF, Hubbard J, Groteluschen A, Chhun B. Adolescent survivors of massive childhood trauma in cambodia: life events and current symptoms. Journal of Traumatic Stress. 1992;5(4):589–99. doi: 10.1002/jts.2490050408

[102] Gammoh O, Sayaheen B, Alsous M, Al-Smadi A, Al-Jaidi B, Aljabali AAA. The prevalence and correlates of depression, anxiety, and Insomnia among camp residing palestinian women migrants during the outbreak of the war on Gaza: a cross-sectional study from Jordan. Medicina (Kaunas). 2024;60(8):1228. doi: 10.3390/medicina60081228 39202508 PMC11356496

[103] van Straten A, Weinreich KJ, Fábián B, Reesen J, Grigori S, Luik AI, et al. The prevalence of insomnia disorder in the general population: a meta-analysis. J Sleep Res. 2025;34(5):e70089. doi: 10.1111/jsr.70089 40369835 PMC12426706

[104] Kocevska D, Lysen TS, Dotinga A, Koopman-Verhoeff ME, Luijk MPCM, Antypa N, et al. Sleep characteristics across the lifespan in 1.1 million people from the Netherlands, United Kingdom and United States: a systematic review and meta-analysis. Nat Hum Behav. 2021;5(1):113–22. doi: 10.1038/s41562-020-00965-x 33199855

[105] Etindele Sosso FA, Torres Silva F, Queiroz Rodrigues R, Carvalho MM, Zoukal S, Zarate GC. Prevalence of sleep disturbances in latin american populations and its association with their socioeconomic status-a systematic review and a meta-analysis. J Clin Med. 2023;12(24):7508. doi: 10.3390/jcm12247508 38137577 PMC10743597

[106] Suleiman KH, Yates BC. Translating the insomnia severity index into Arabic. J Nurs Scholarsh. 2011;43(1):49–53. doi: 10.1111/j.1547-5069.2010.01374.x 21342424

[107] Morin CM, Belleville G, Bélanger L, Ivers H. The insomnia severity index: psychometric indicators to detect insomnia cases and evaluate treatment response. Sleep. 2011;34(5):601–8. doi: 10.1093/sleep/34.5.601 21532953 PMC3079939

[108] Chen P, Lam MI, Si TL, Zhang L, Balbuena L, Su Z, et al. The prevalence of poor sleep quality in the general population in China: a meta-analysis of epidemiological studies. Eur Arch Psychiatry Clin Neurosci. 2024;274(7):1–14. doi: 10.1007/s00406-024-01764-5 38429554

[109] Hinz A, Glaesmer H, Brähler E, Löffler M, Engel C, Enzenbach C, et al. Sleep quality in the general population: psychometric properties of the Pittsburgh Sleep Quality Index, derived from a German community sample of 9284 people. Sleep Med. 2017;30:57–63. doi: 10.1016/j.sleep.2016.03.008 28215264

[110] Msaad S, Ketata N, Fidha S, Gargouri R, Talaa HA, Wadhane I, et al. Sleep habits and quality among war and conflict-affected Palestinian adults in the Gaza strip. Sleep Med. 2023;102:90–104. doi: 10.1016/j.sleep.2022.12.025 36634603

[111] Basishvili T, Eliozishvili M, Maisuradze L, Lortkipanidze N, Nachkebia N, Oniani T, et al. Insomnia in a displaced population is related to war-associated remembered stress. Stress Health. 2012;28(3):186–92. doi: 10.1002/smi.1421 22282401

[112] Yang X, You L, Jin D, Zou X, Yang H, Liu T. A community-based cross-sectional study of sleep quality among internal migrant workers in the service industry. Compr Psychiatry. 2020;97:152154. doi: 10.1016/j.comppsych.2019.152154 31884329

[113] United Nations High Commissioner for Refugees. Refugee statistics. Geneva: UNHCR; 2024. https://www.unhcr.org/refugee-statistics

[114] Morin CM, Drake CL, Harvey AG, Krystal AD, Manber R, Riemann D, et al. Insomnia disorder. Nat Rev Dis Primers. 2015;1:15026. doi: 10.1038/nrdp.2015.26 27189779

[115] Blackmore R, Boyle JA, Fazel M, Ranasinha S, Gray KM, Fitzgerald G, et al. The prevalence of mental illness in refugees and asylum seekers: a systematic review and meta-analysis. PLoS Med. 2020;17(9):e1003337. doi: 10.1371/journal.pmed.1003337 32956381 PMC7505461

[116] Higgins JPT. Commentary: Heterogeneity in meta-analysis should be expected and appropriately quantified. Int J Epidemiol. 2008;37(5):1158–60. doi: 10.1093/ije/dyn204 18832388

[117] Jou YC, Pace-Schott EF. Call to action: Addressing sleep disturbances, a hallmark symptom of PTSD, for refugees, asylum seekers, and internally displaced persons. Sleep Health. 2022;8(6):593–600. doi: 10.1016/j.sleh.2022.09.003 36511279 PMC9757843

[118] Dumser B, Müller CL, Ehring T, Werner GG, Koch T. Treating sleep disturbances in refugees and asylum seekers: results from a randomized controlled pilot trial evaluating the STARS group intervention. Eur J Psychotraumatol. 2025;16(1):2455248. doi: 10.1080/20008066.2025.2455248 39927405 PMC11812105

