# Supplementary material for: Insomnia and poor sleep quality in refugee and asylum-seeking populations: A systematic review and meta-analysis
Source: PLoS One. 2026 Jul 2;21(7):e0352964. doi: 10.1371/journal.pone.0352964 (PMC13327149; doi:10.1371/journal.pone.0352964)
Supplement: S1 File — (DOCX) [file pone.0352964.s001.docx]

**Supplementary Information**

Additional analysis and plots are presented by outcome.

[**1. Insomnia Severity Index (ISI) 4**](#_heading=)

[1.1. Forest plot omitting Bruck, D 2021 4](#_heading=h.bqcjfxwnqer0)

[1.2. Baujat plot 5](#_heading=h.padwcnodxana)

[1.3. Leave-one-out plot 6](#_heading=h.b93lxcxh6i9i)

[1.4. Influence plots 7](#_heading=h.3yglcp4mn7gw)

[1.5. Funnel Plot 8](#_heading=h.amkdhyo1ea1v)

[**2. Nightmare Prevalence in Adults 9**](#_heading=)

[2.1. Forest plot 9](#_heading=h.53y87gln8as8)

[2.2. Baujat plot 10](#_heading=h.q0n45lykg6xa)

[2.3. Leave-one-out plot 11](#_heading=h.imoabc60i3io)

[2.4. Influence plots 12](#_heading=h.nbz769rzfdt6)

[2.5. Funnel plot 13](#_heading=h.w0zou6acbhqi)

[**3. Nightmare Prevalence in Children 14**](#_heading=)

[3.1. Forest plot 14](#_heading=h.7ygvnl1q7mq3)

[3.2. Baujat plot 15](#_heading=h.br81gdwj41lz)

[3.3. Leave-one-out plot 16](#_heading=h.ummrnvvwbfmw)

[3.4. Influence plots 17](#_heading=h.dy81bdtv2lv)

[**4. Pittsburgh Sleep Quality Index (PSQI) 18**](#_heading=)

[4.1. Baujat plot 18](#_heading=h.wtdqmbcs5uh9)

[4.2. Leave-one-out plot 19](#_heading=h.s296i1f51fup)

[4.3. Influence plots 20](#_heading=h.mf8tq0ufcimu)

[**5. Sleep Adversities Prevalence in Adults 21**](#_heading=)

[5.1. Forest plot 21](#_heading=h.avavx5788us7)

[5.2. Baujat plot 22](#_heading=h.3l29ihpzmt9)

[5.3. Leave-one-out plot 23](#_heading=h.jk5xj19u2nb9)

[5.4. Influence plots 24](#_heading=h.c9dxztgagidm)

[5.5. Funnel plot 25](#_heading=h.90mmngf2c97n)

[5.6. Subgroup analysis forest plot (by instrument type) 26](#_heading=h.l3aonknue5u4)

[5.7. Subgroup analysis forest plot (by quality) 27](#_heading=h.nap3y4jpcbdr)

[5.8. Subgroup analysis forest plot (by health establishment) 28](#_heading=h.qf8shh12r9f7)

[**6. Sleep Adversities Prevalence in Children 29**](#_heading=h.tsvsie6m94nv)

[6.1. Forest plot 29](#_heading=h.krn1x3jwqzr)

[6.2. Baujat plot 30](#_heading=h.xjvvspcx452c)

[6.3. Leave-one-out plot 31](#_heading=h.t3b23xgyrl97)

[6.4. Influence plots 32](#_heading=h.7u6ycso3bzi7)

[6.5. Funnel plot 33](#_heading=h.y3bjti16tice)

[6.6. Subgroup analysis forest plot (by instrument type) 34](#_heading=h.euytpkx3k9pu)

[6.7. Subgroup analysis forest plot (by quality) 35](#_heading=h.g4jrqidm9daf)

[6.8. Subgroup analysis forest plot (by health establishment) 36](#_heading=h.py8o65zg9j7)

[**7. Sleep Duration Mean in Adults 37**](#_heading=)

[7.1. Forest plot 37](#_heading=h.fyg3ivtbv965)

[7.2. Baujat plot 38](#_heading=h.gnzoy7ph4d2a)

[7.3. Leave-one-out plot 39](#_heading=h.b8smnygx6drk)

[7.4. Influence plots 40](#_heading=h.pgrz0130njij)

[**8. Sleep Duration Mean in Children and Adolescents 41**](#_heading=)

[8.1. Forest plot 41](#_heading=h.gjwcroim8o3l)

[8.2. Baujat plot 42](#_heading=h.m08nh9evslsa)

[8.3. Leave-one-out plot 43](#_heading=h.apwcd4fzw5gv)

[8.4. Influence plots 44](#_heading=h.qluw3s458t3t)

[**9. Sleep Latency Adults 45**](#_heading=)

[9.1. Forest plot 45](#_heading=h.hi436a3ptv5h)

[9.2. Baujat plot 46](#_heading=h.ba18dd2fm98y)

[9.3. Leave-one-out plot 47](#_heading=h.wdg8q6up94vq)

[9.4. Influence plots 48](#_heading=h.lxnn6rog6nfj)

[**10. Sleep Latency Children and Adolescents 49**](#_heading=)

[10.1. Forest plot 49](#_heading=h.7cw2krspg8lz)

[10.2. Baujat plot 49](#_heading=h.aiw4e84pzhb7)

[10.3. Leave-one-out plot 51](#_heading=h.duze2n6pu7l3)

[**11. Sleep Quality 4-Points Likert Scale Mean in Adults 52**](#_heading=)

[11.1. Forest plot 52](#_heading=h.qzcvwfhyya83)

[11.2. Baujat plot 53](#_heading=h.ez6qymv6bxx0)

[11.3. Leave-one-out plot 54](#_heading=h.lrnqqkxog9hi)

[11.4. Influence plots 55](#_heading=h.yod855f6boag)

[**12. Sleep Quality 4-Points Likert Scale Mean in Children and Adolescents 57**](#_heading=)

[12.1. Forest plot 57](#_heading=h.jablxxrokc7u)

[12.2. Baujat plot 58](#_heading=h.hp07v9falzrc)

[12.3. Leave-one-out plot 59](#_heading=h.bq35gm133d6l)

[12.4. Influence plots 60](#_heading=h.d4ohtf3cowsp)

[**14. Supplementary tables 61**](#_heading=h.x3s34p8kuvp9)

[Table 1. Search strategies. 61](#_heading=h.48s3cbseqnqx)

[Table 2. Newcastle-Ottawa Scale (NOS) for cohort studies. 62](#_heading=h.r4gf5ykgrn7e)

[Table 3. Newcastle-Ottawa Scales (NOS) adapted for cross-sectional studies. 64](#_heading=h.j87fzim9yras)

[Table 4. Moderators literature gap. 66](#_heading=h.ds0sr21v3z4)

[Table 5. Terminology and definitions. 70](#_heading=h.5pp1761pop32)

[Table 6. Meta-regression models. 77](#_heading=h.679dv97n1on7)

[Table 7. PRISMA Checklist 79](#_heading=h.jdvdalg9297)

# 1. Insomnia Severity Index (ISI)

## 1.1. Forest plot omitting Bruck, D 2021


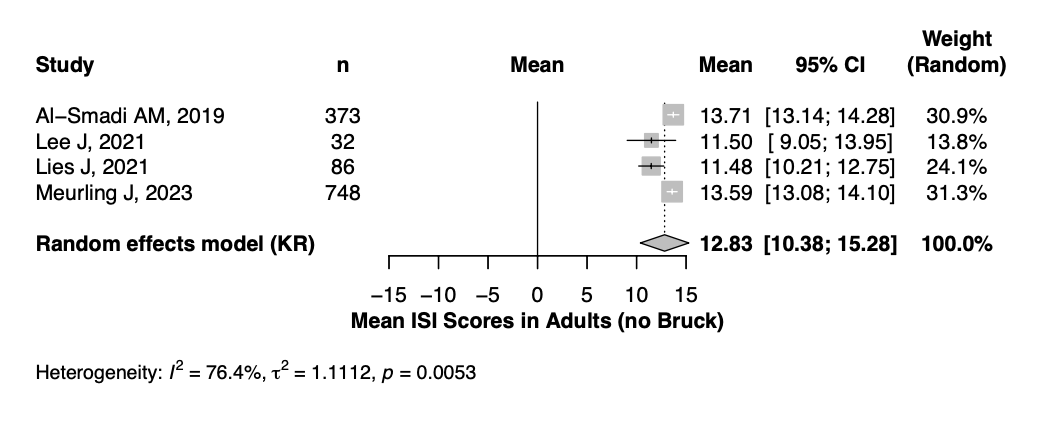


## 1.2. Baujat plot


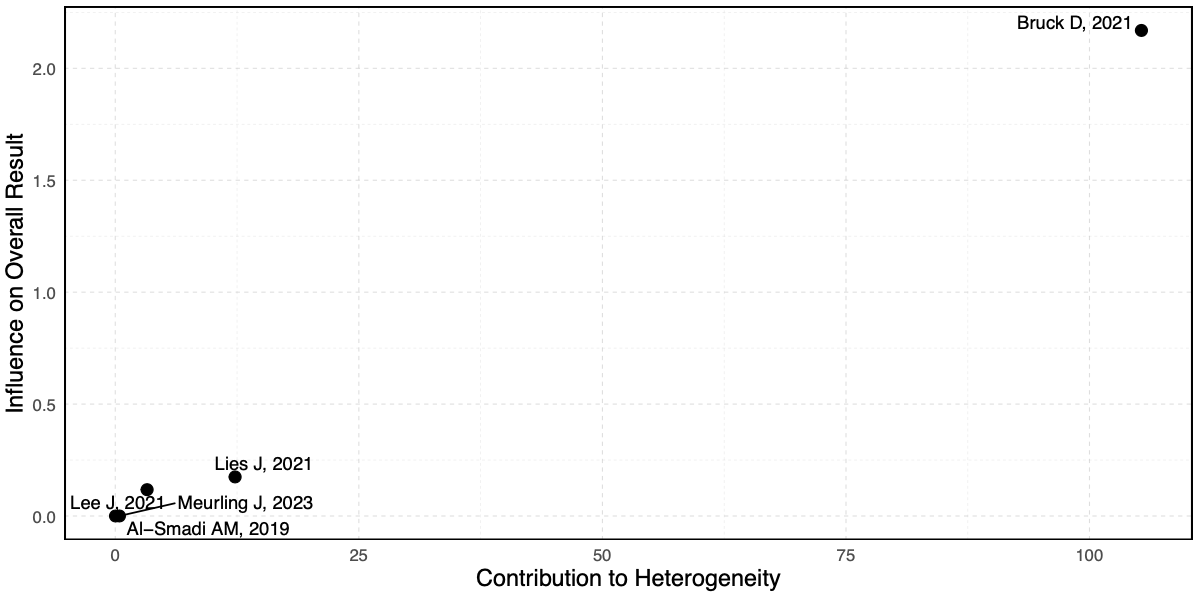


## 1.3. Leave-one-out plot


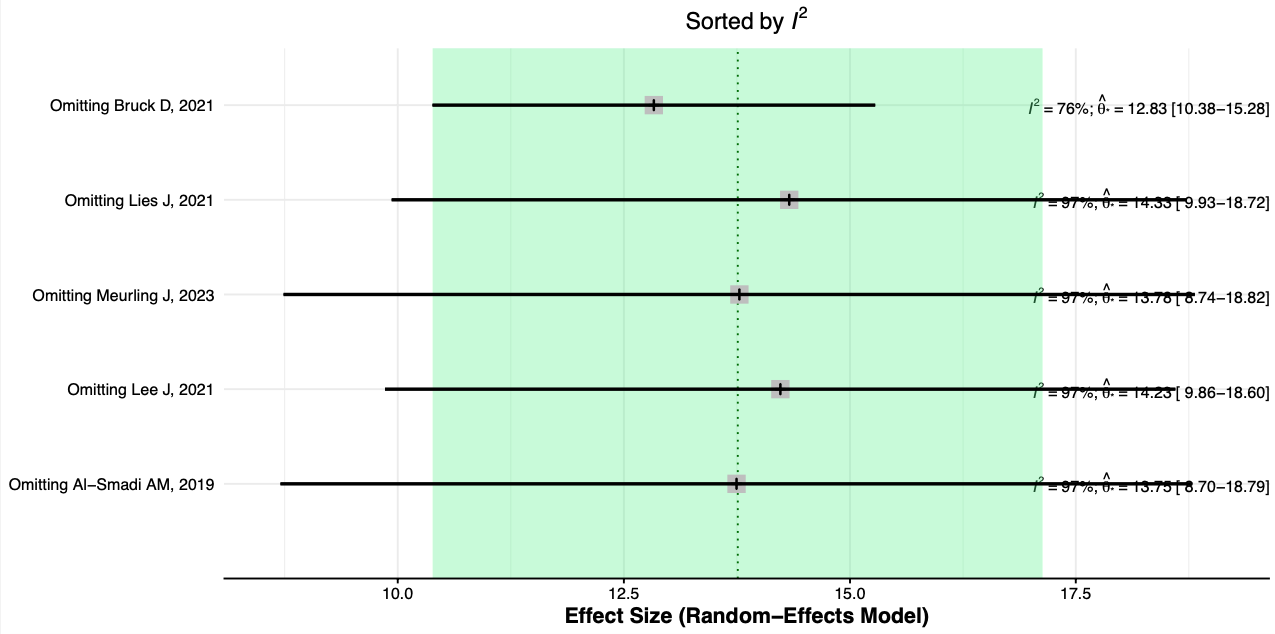


## 1.4. Influence plots


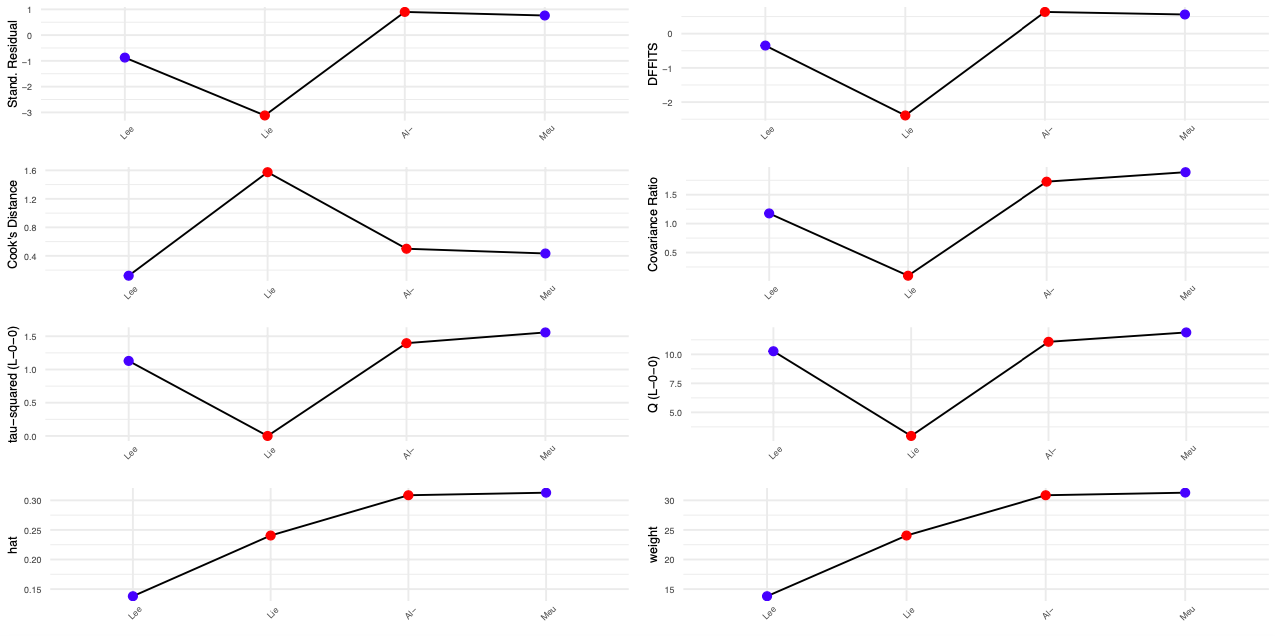


## 1.5. Funnel Plot


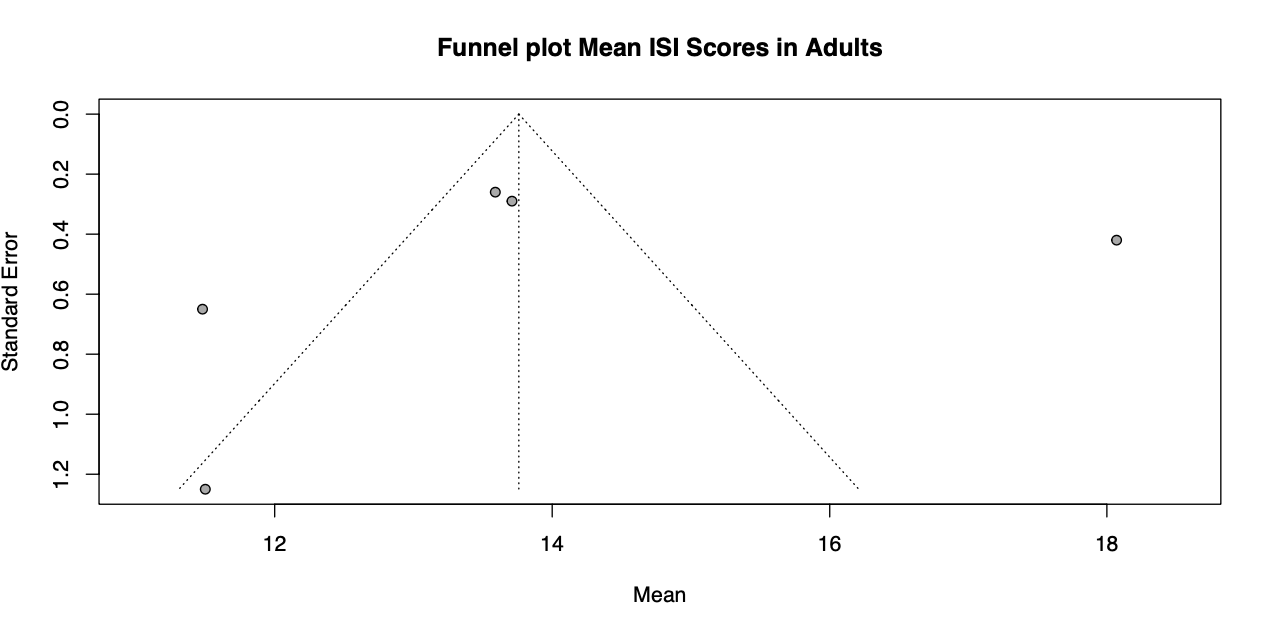


# 2. Nightmare Prevalence in Adults

## 2.1. Forest plot


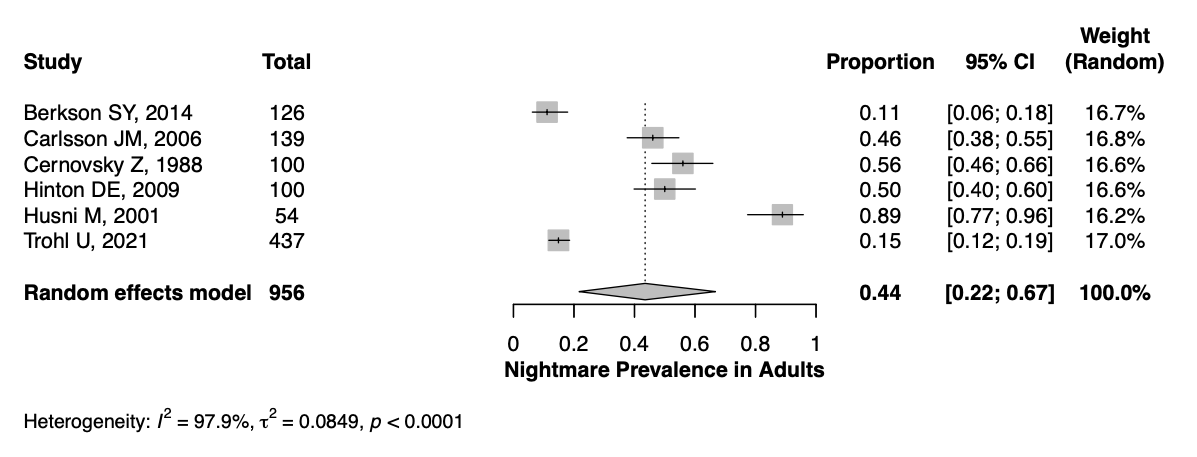


## 2.2. Baujat plot


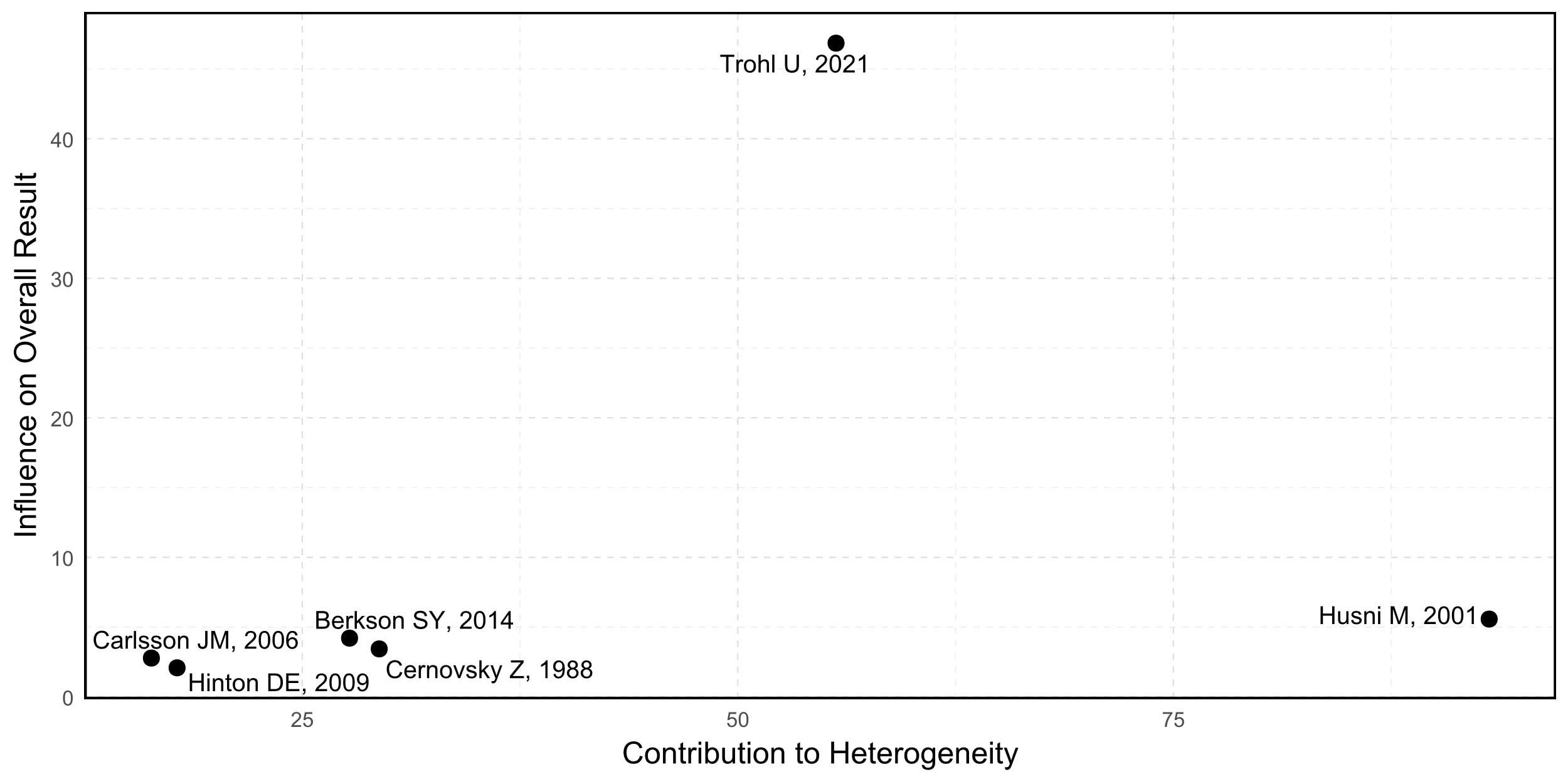


## 2.3. Leave-one-out plot


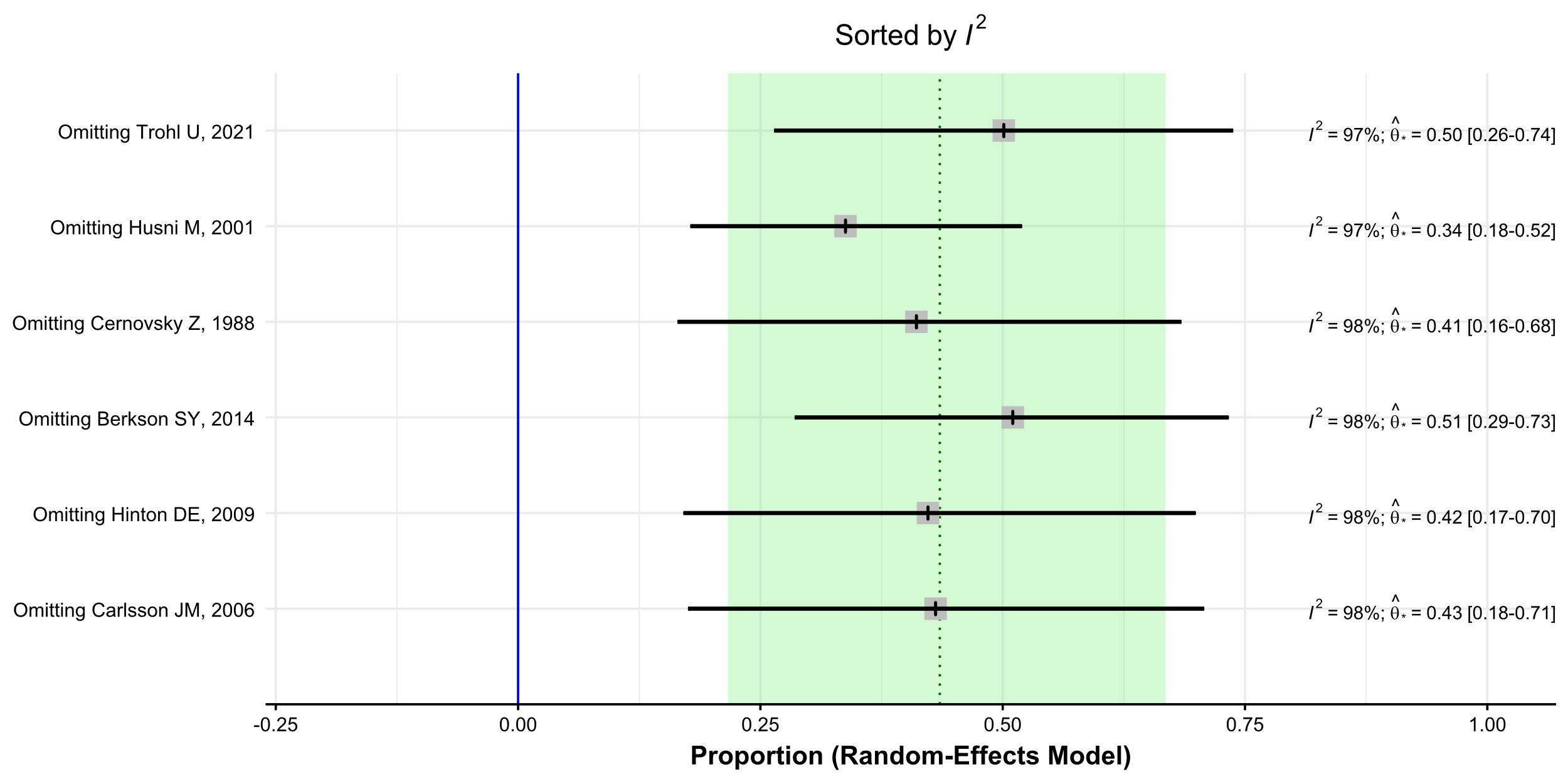


## 2.4. Influence plots


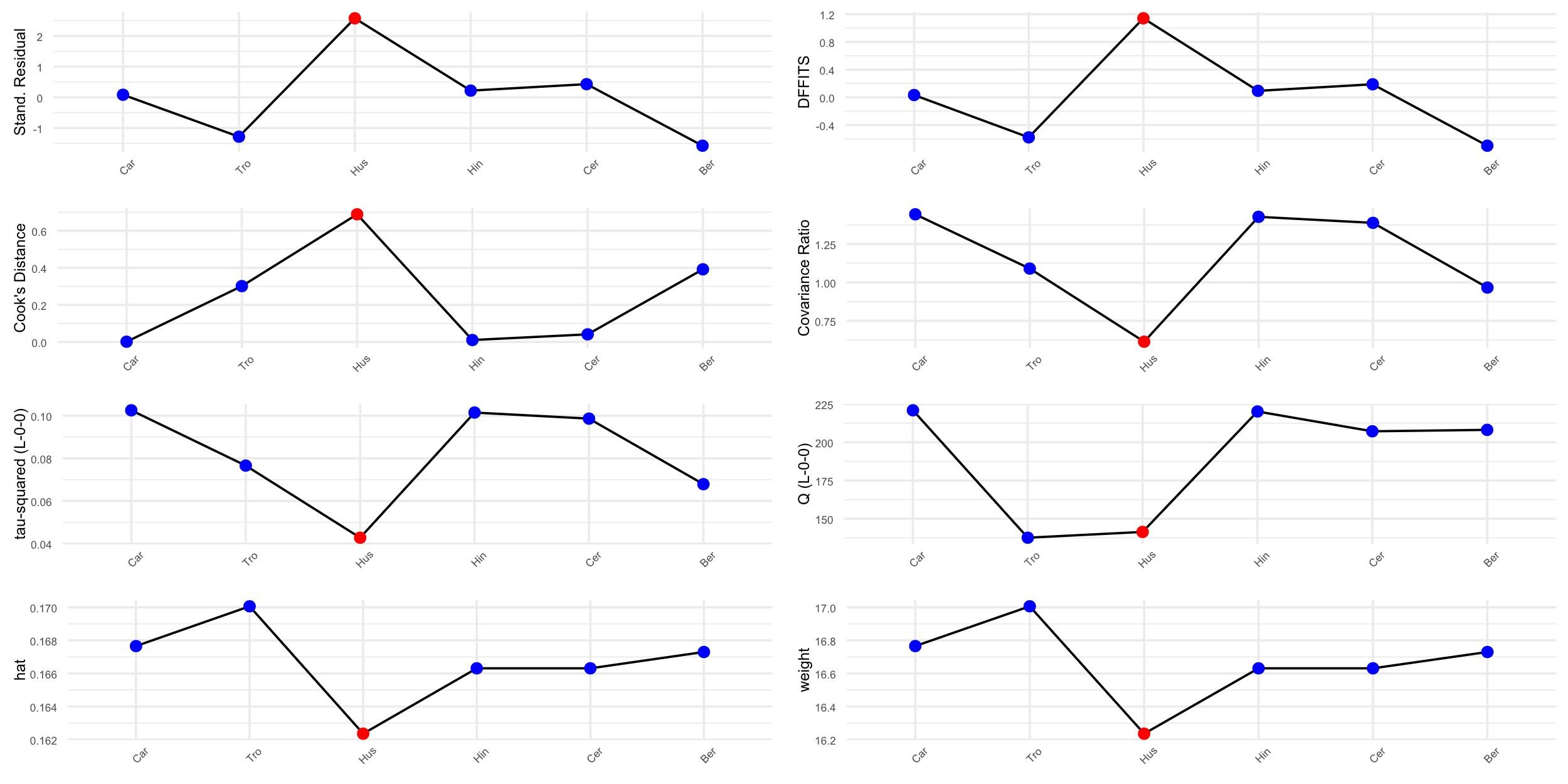


## 2.5. Funnel plot


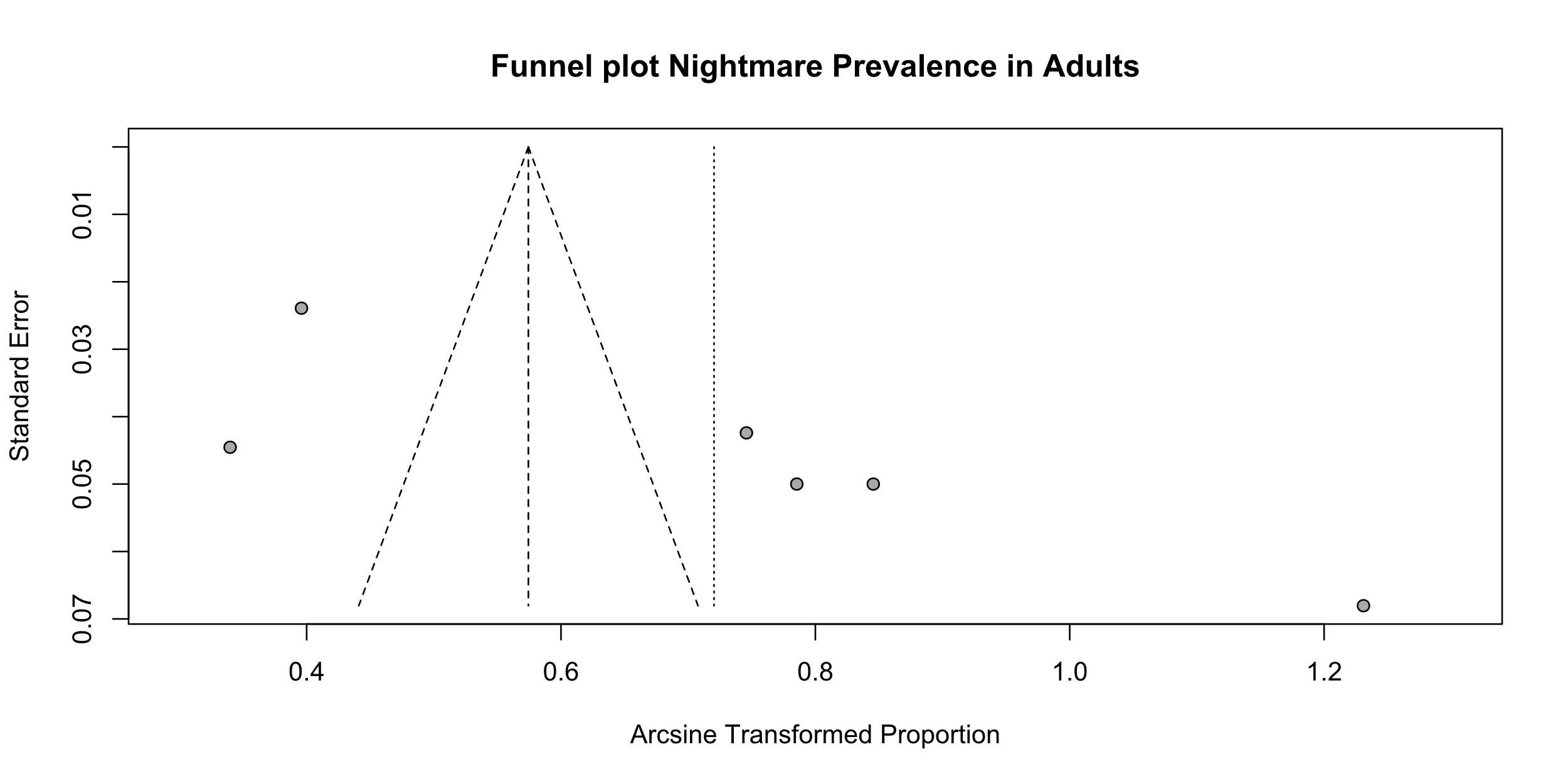


# 3. Nightmare Prevalence in Children

## 3.1. Forest plot


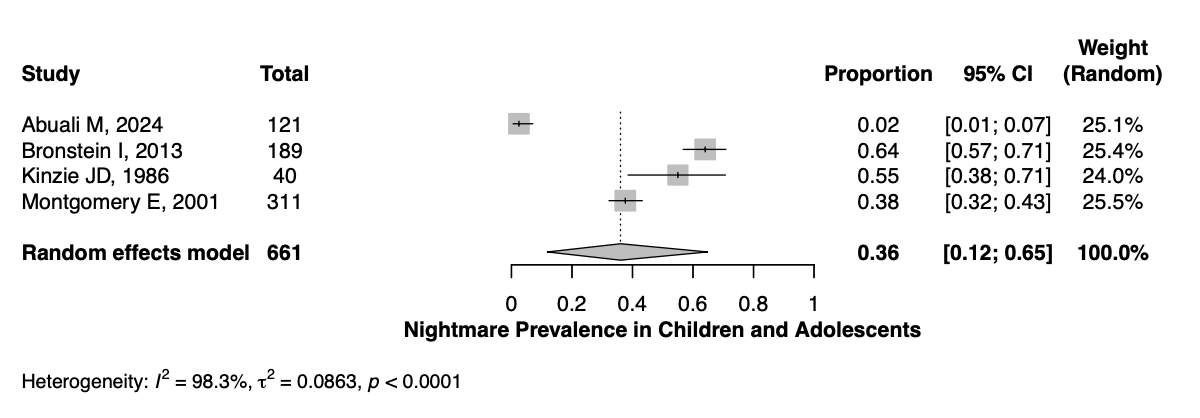


## 3.2. Baujat plot


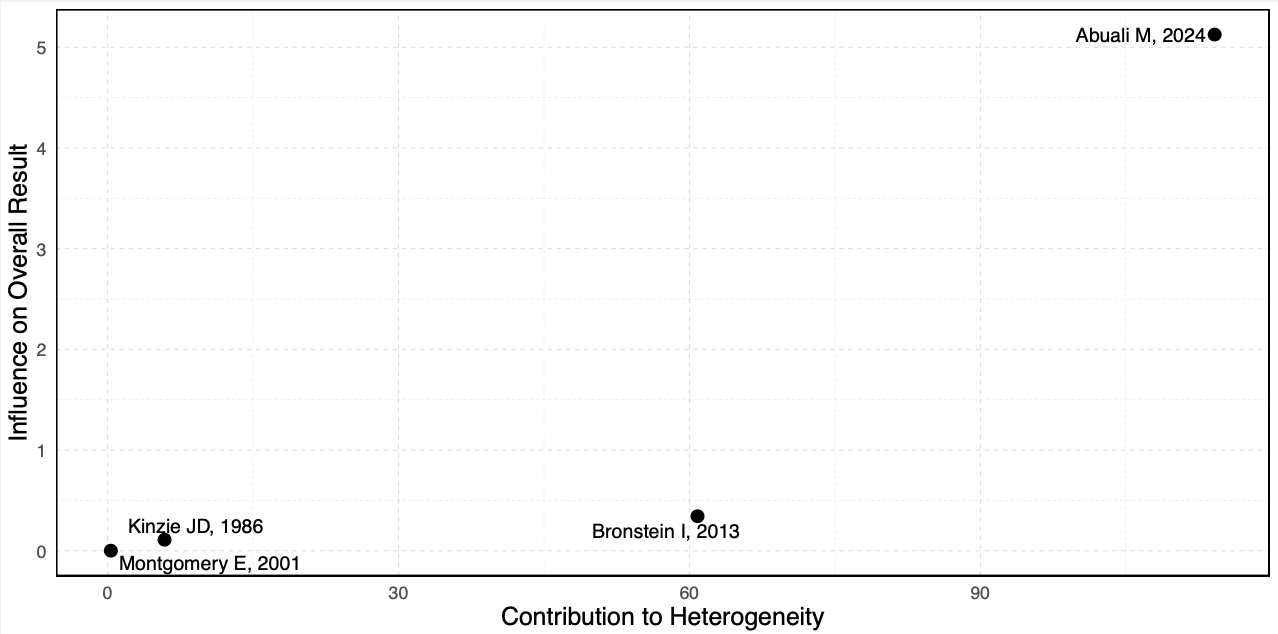


## 3.3. Leave-one-out plot


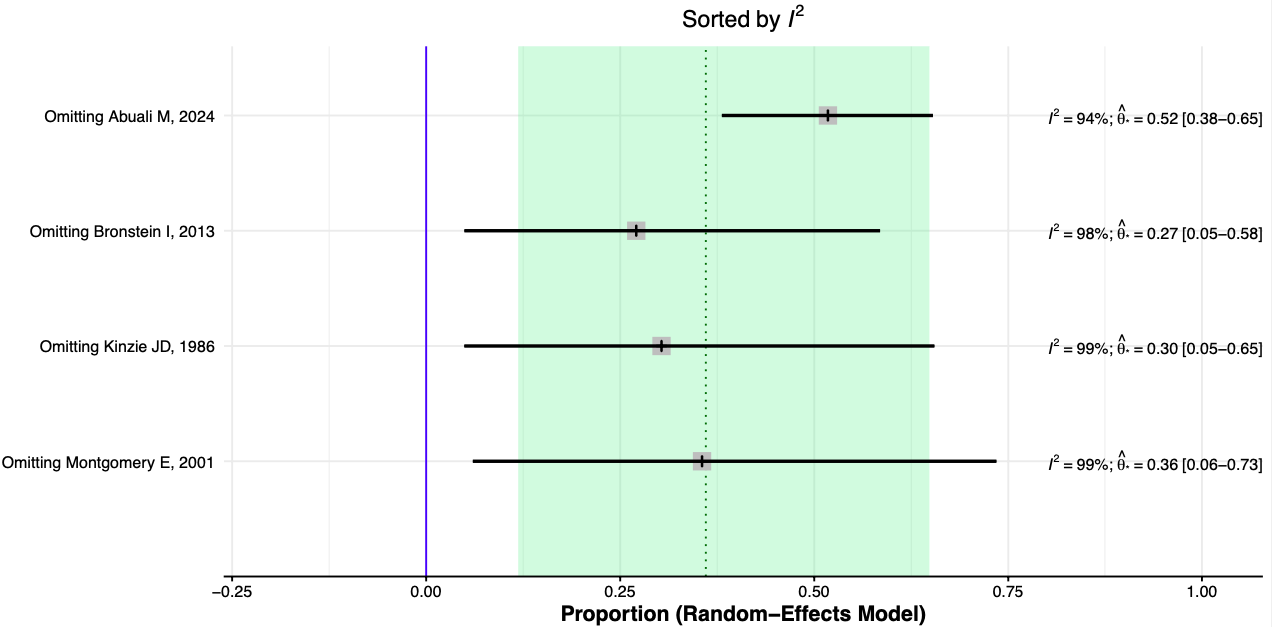


## 3.4. Influence plots


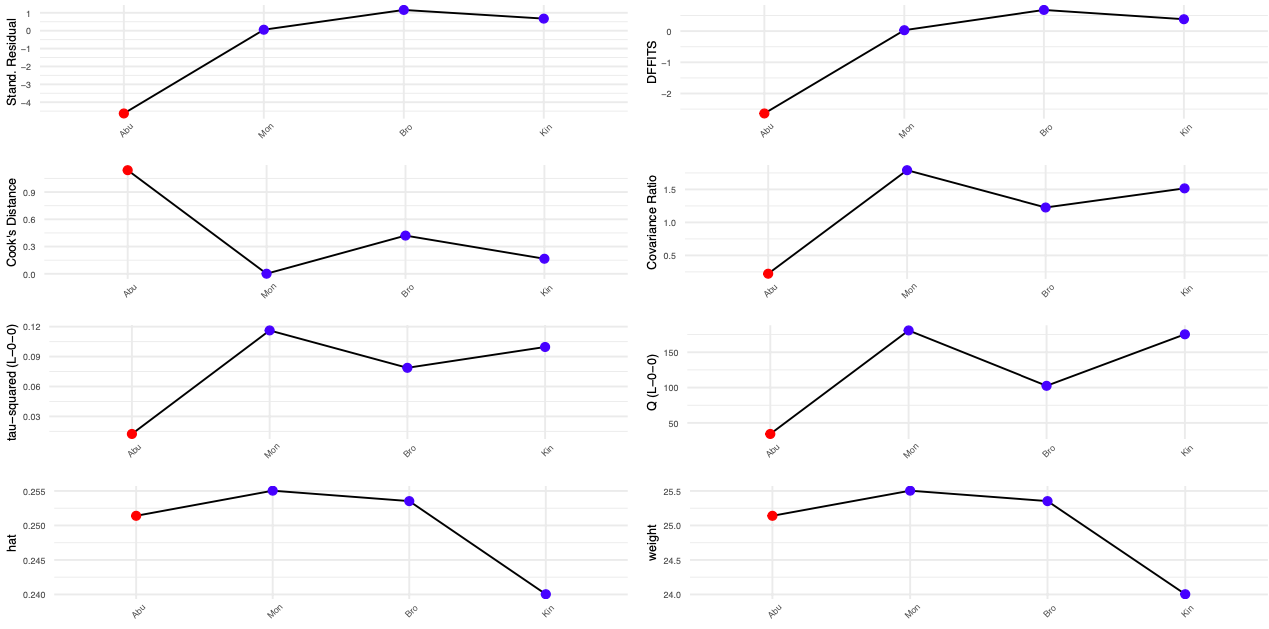


# 4. Pittsburgh Sleep Quality Index (PSQI)

## 4.1. Baujat plot


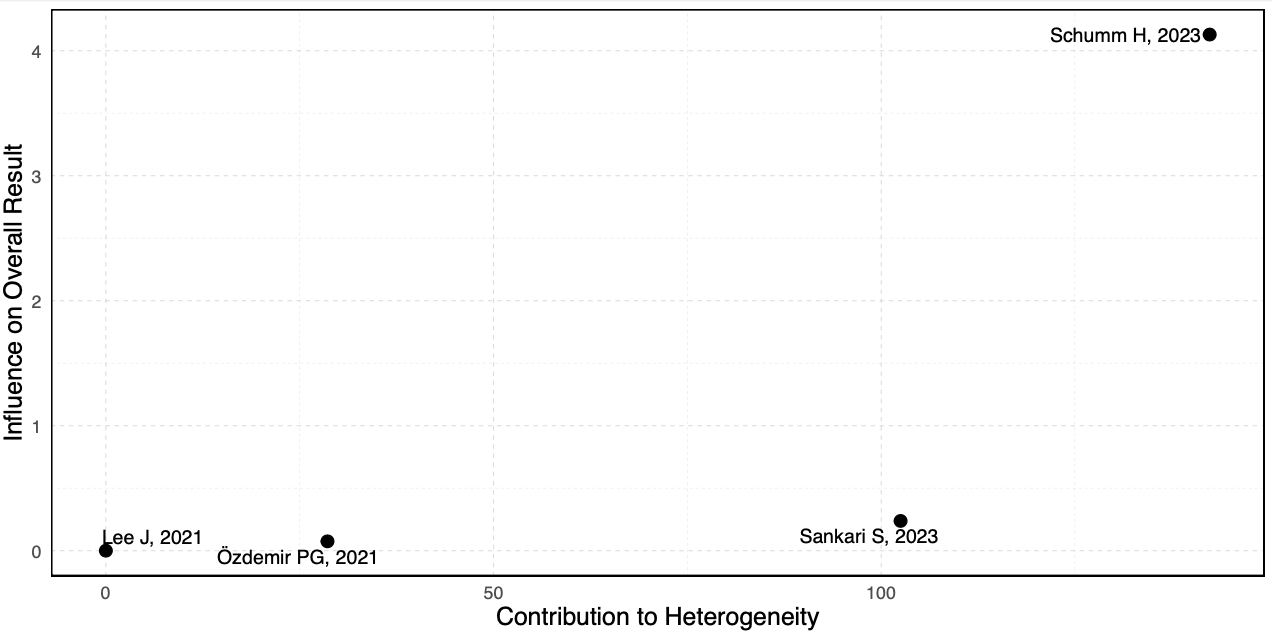


## 4.2. Leave-one-out plot


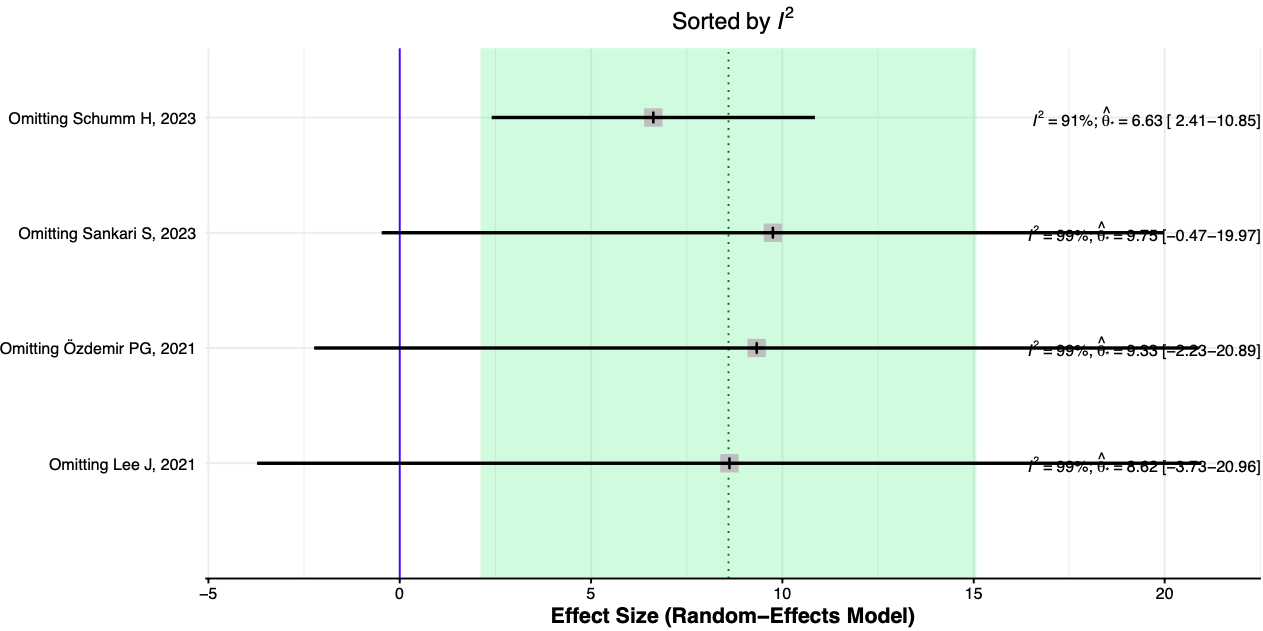


## 4.3. Influence plots


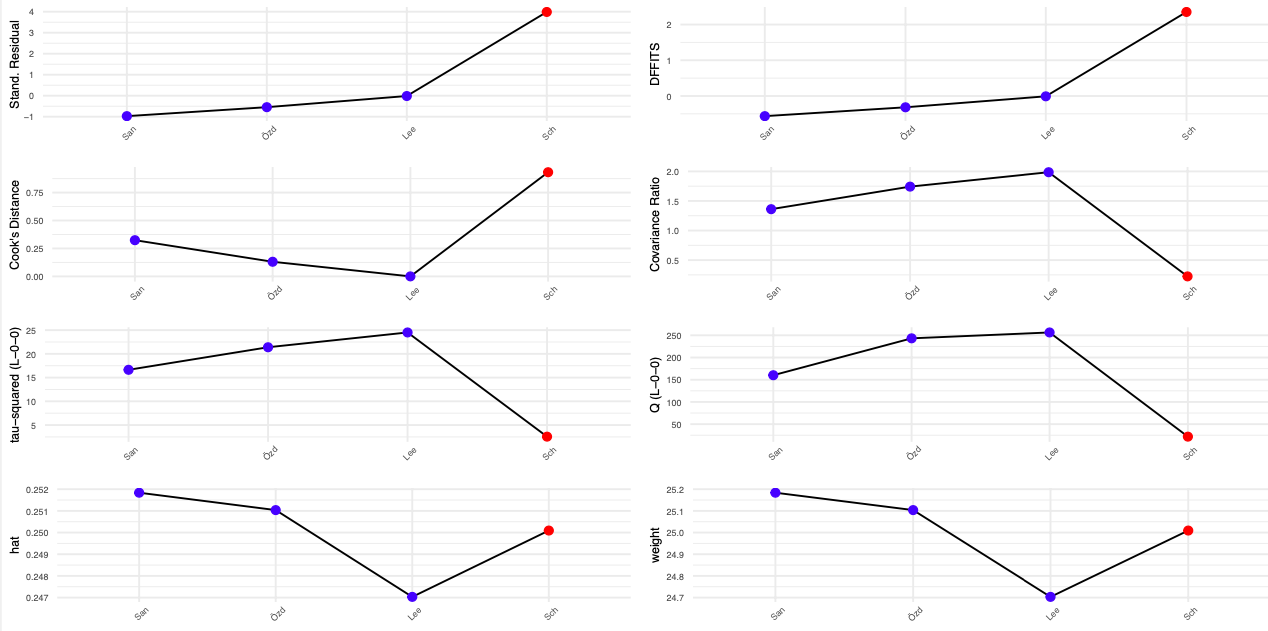


# 5. Sleep Adversities Prevalence in Adults

## 5.1. Forest plot


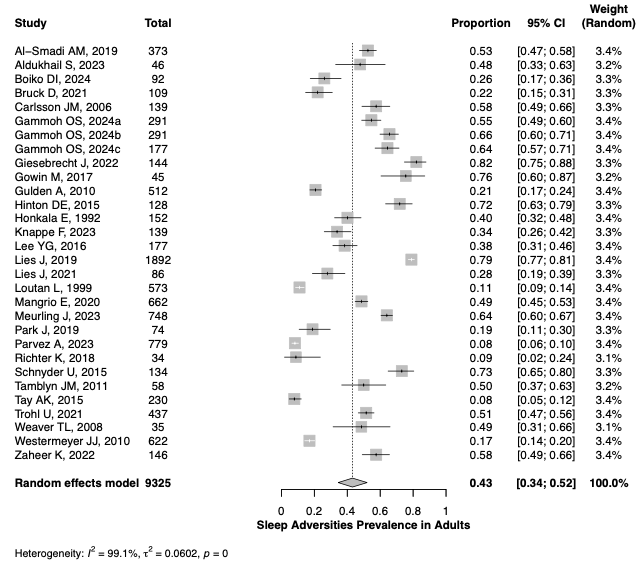


## 5.2. Baujat plot


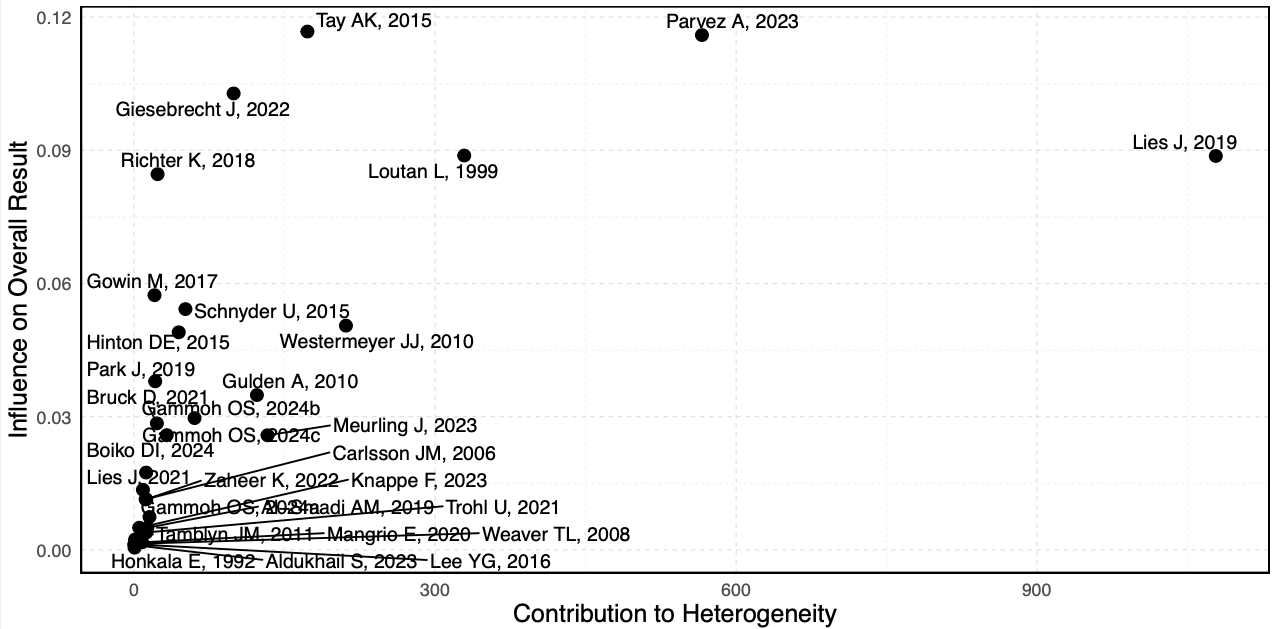


## 5.3. Leave-one-out plot


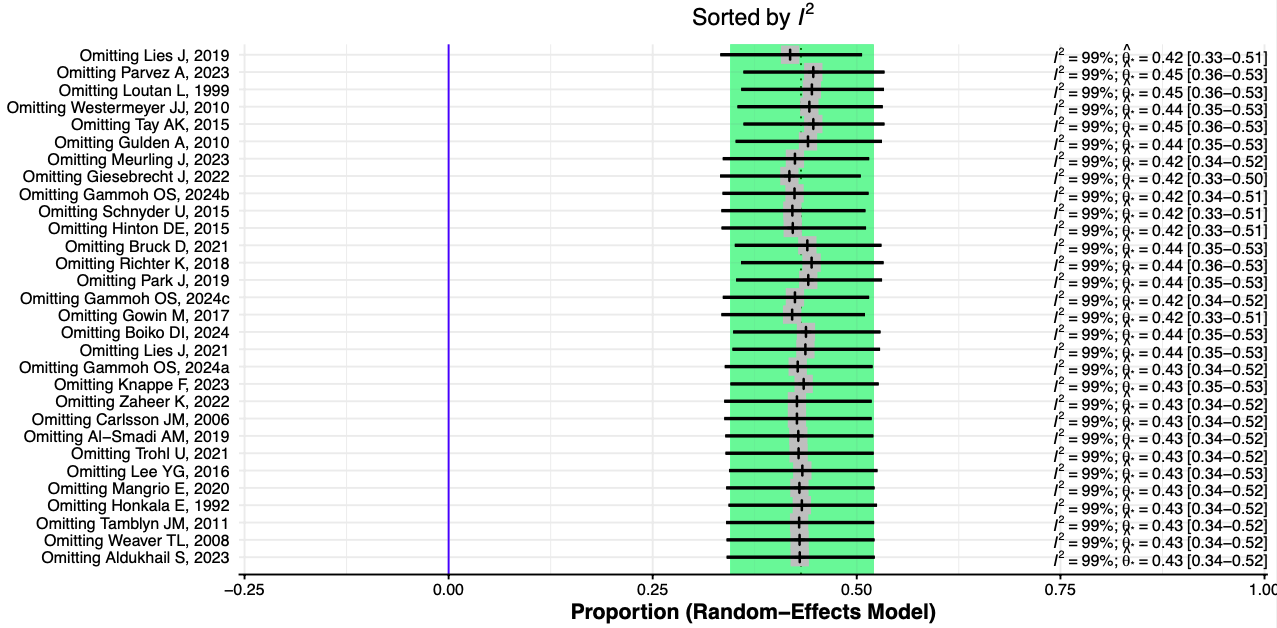


## 5.4. Influence plots


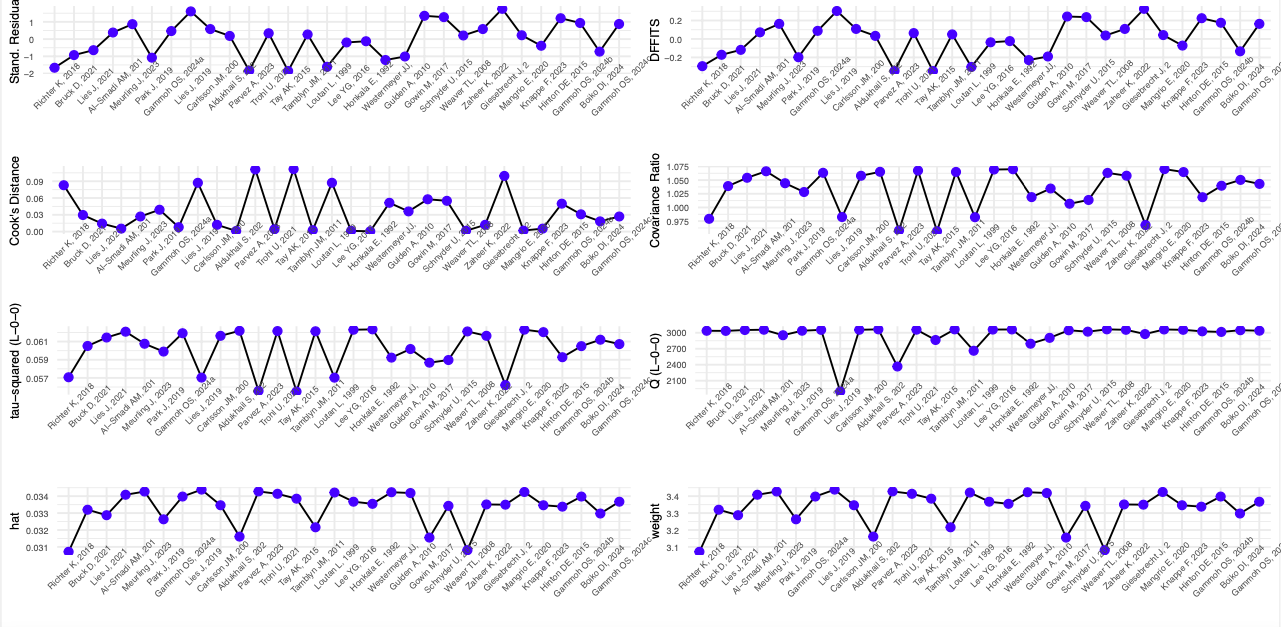


## 5.5. Funnel plot


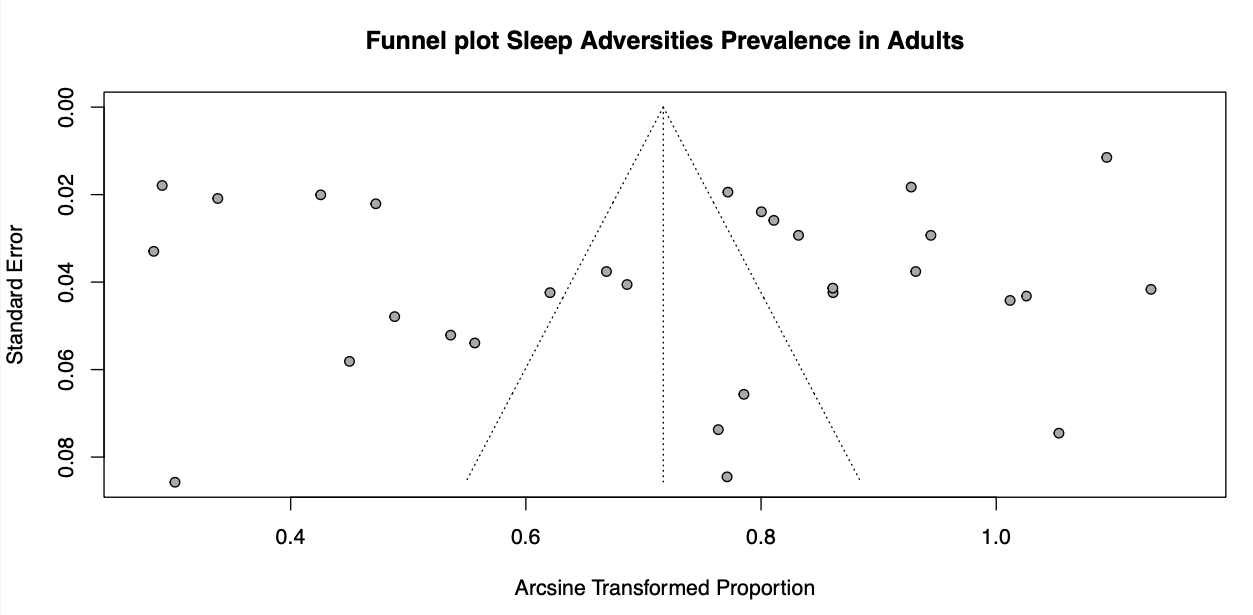


## 5.6. Subgroup analysis forest plot (by instrument type)


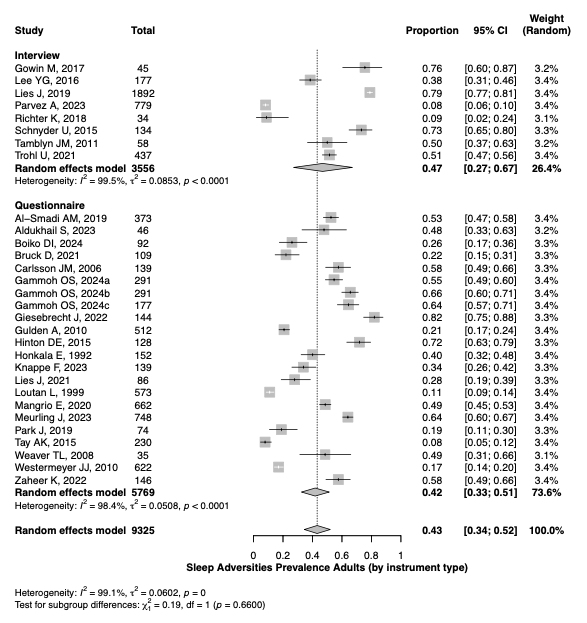


## 5.7. Subgroup analysis forest plot (by quality)


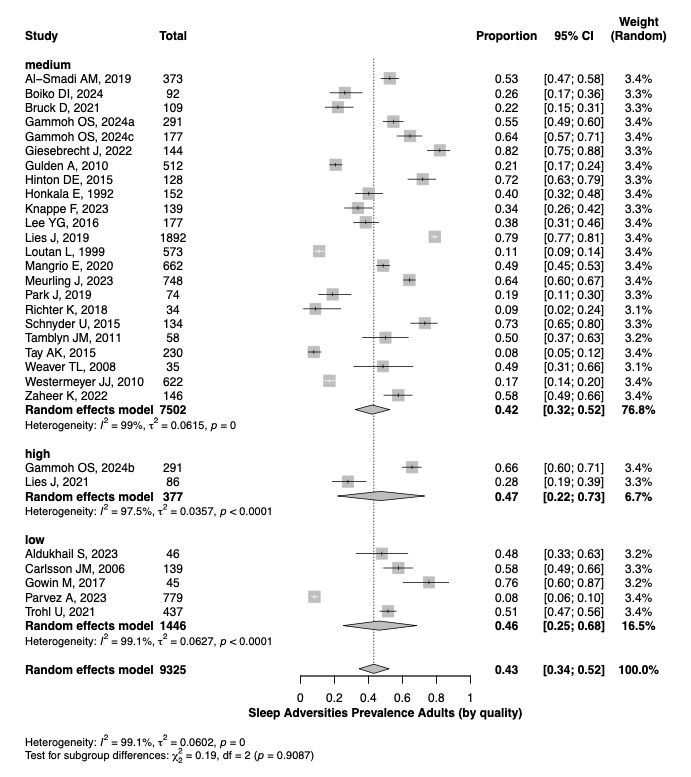


## 5.8. Subgroup analysis forest plot (by health establishment)


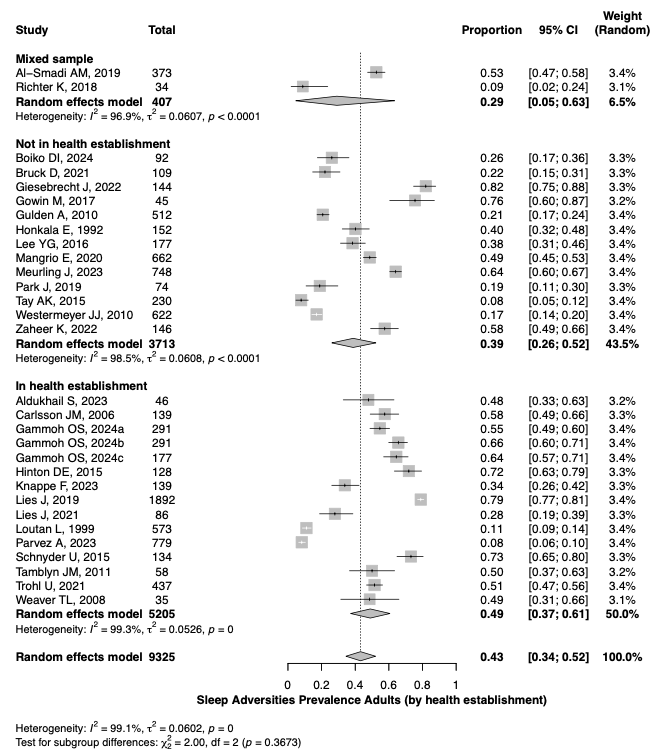


# 6. Sleep Adversities Prevalence in Children

## 6.1. Forest plot


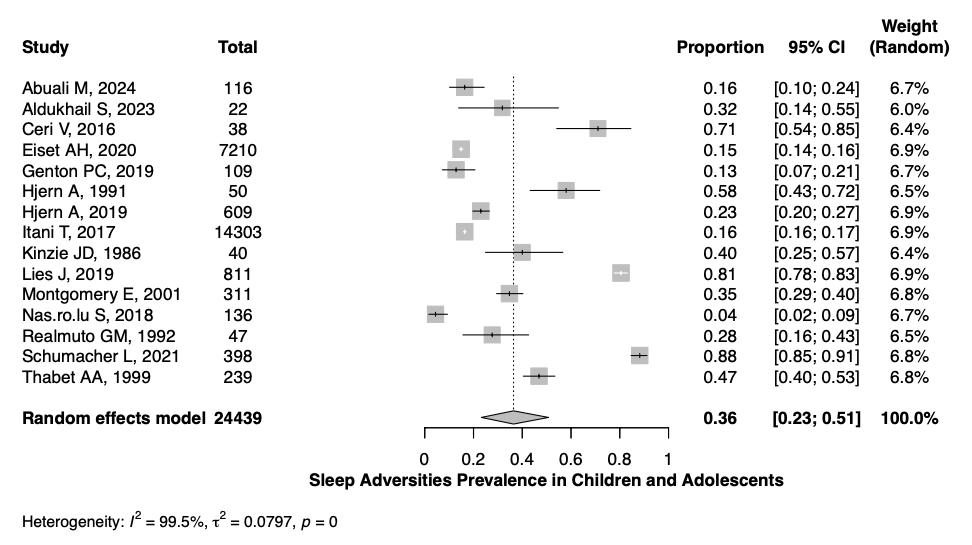


##

##

## 6.2. Baujat plot


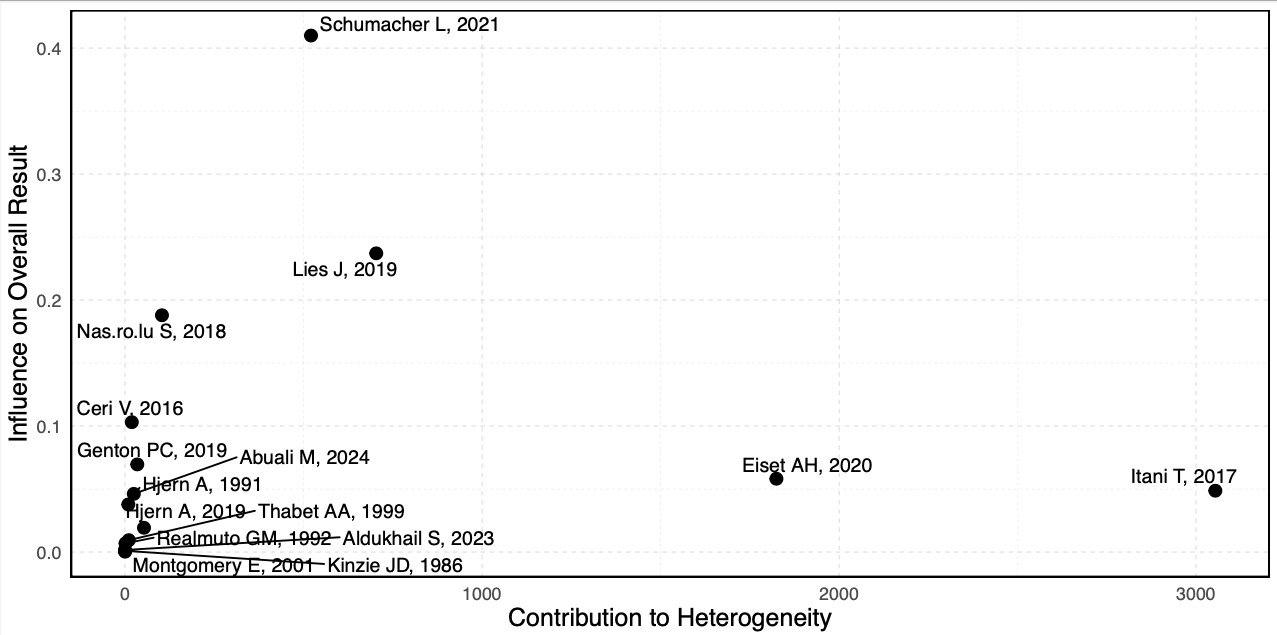


## 6.3. Leave-one-out plot


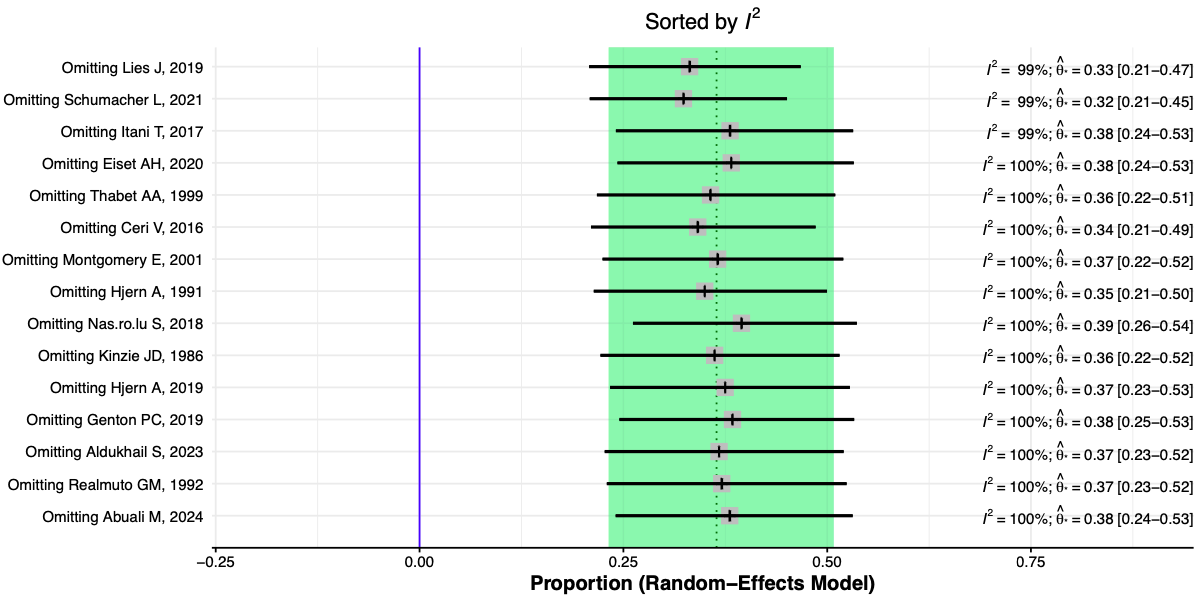


##

## 6.4. Influence plots


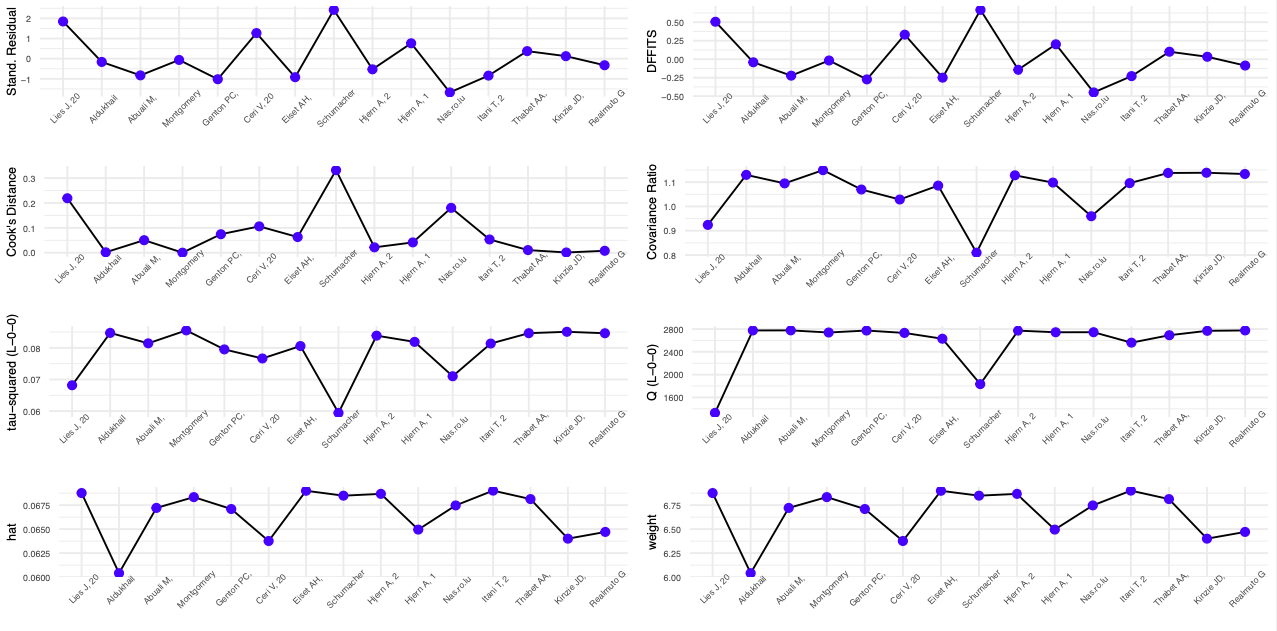


## 6.5. Funnel plot


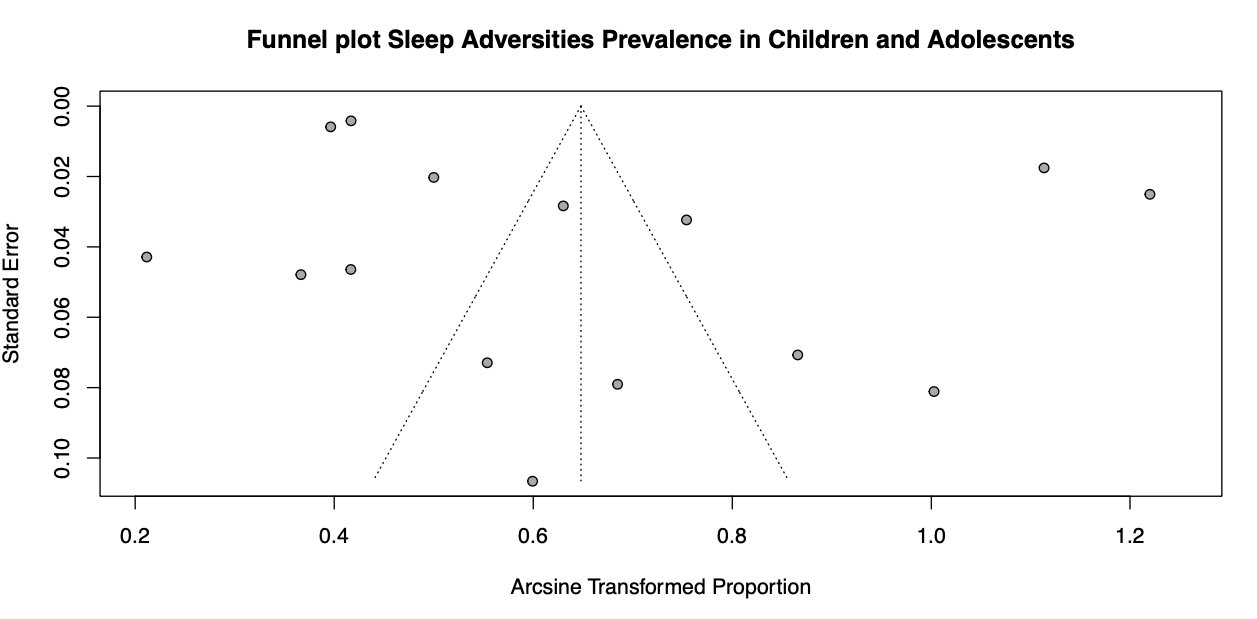


## 6.6. Subgroup analysis forest plot (by instrument type)


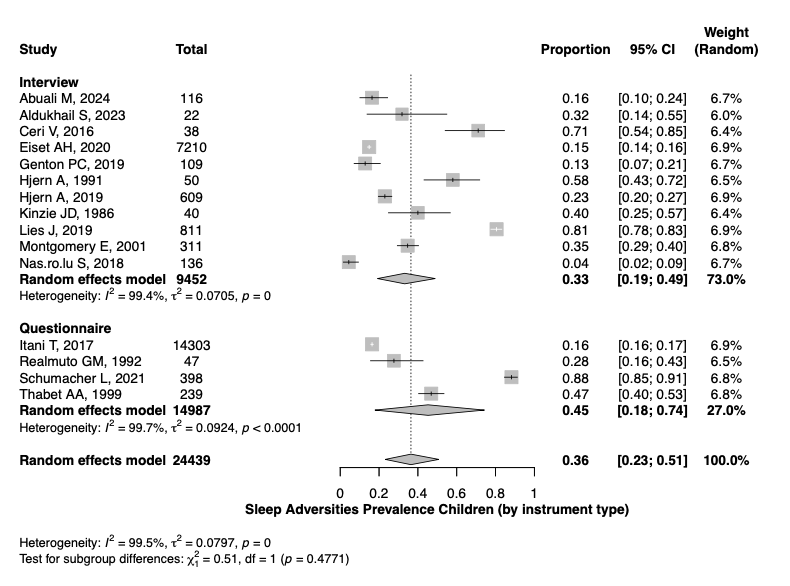


## 6.7. Subgroup analysis forest plot (by quality)


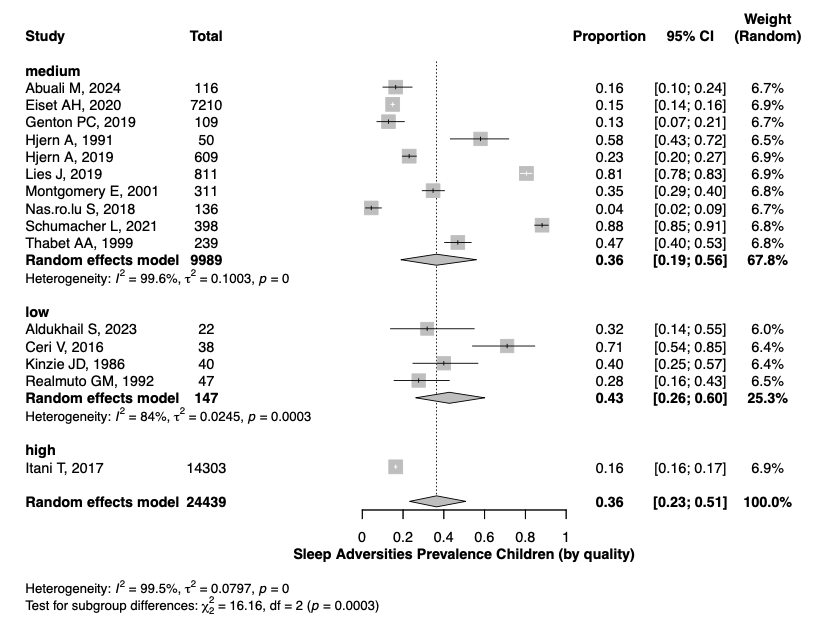


## 6.8. Subgroup analysis forest plot (by health establishment)


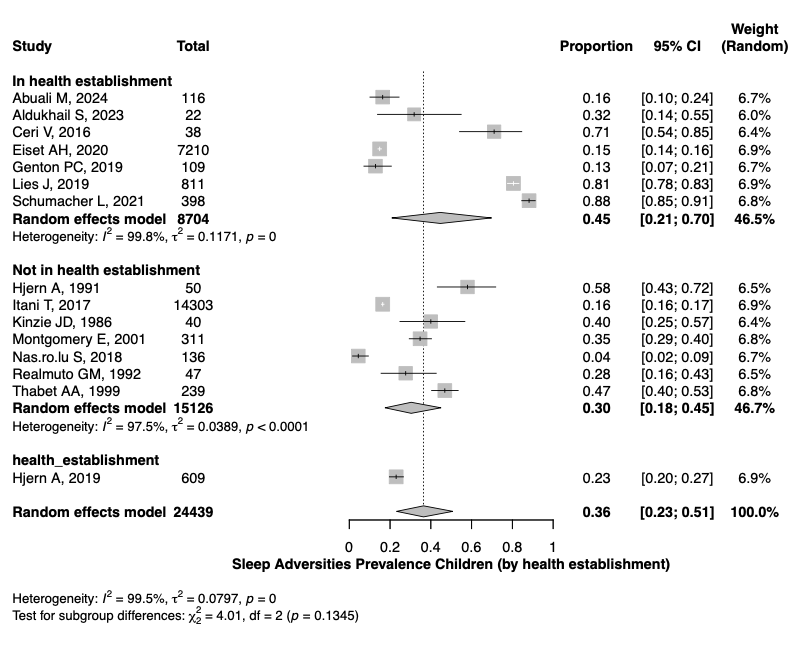


# 7. Sleep Duration Mean in Adults

## 7.1. Forest plot


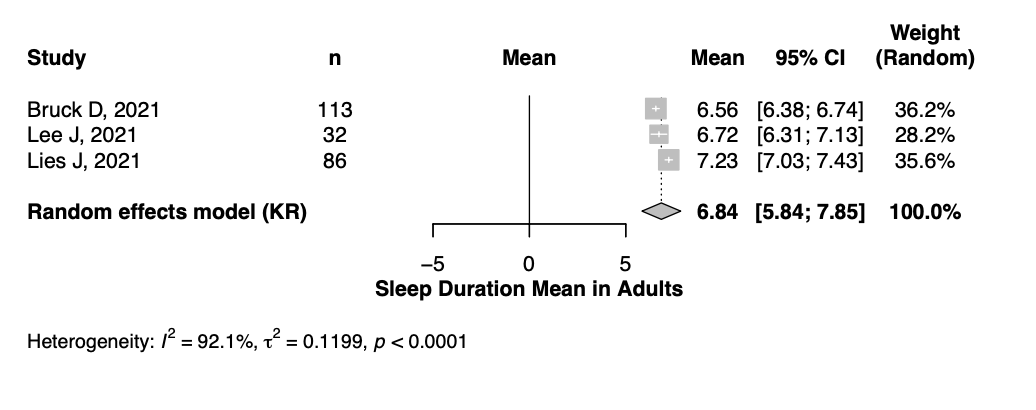


## 7.2. Baujat plot


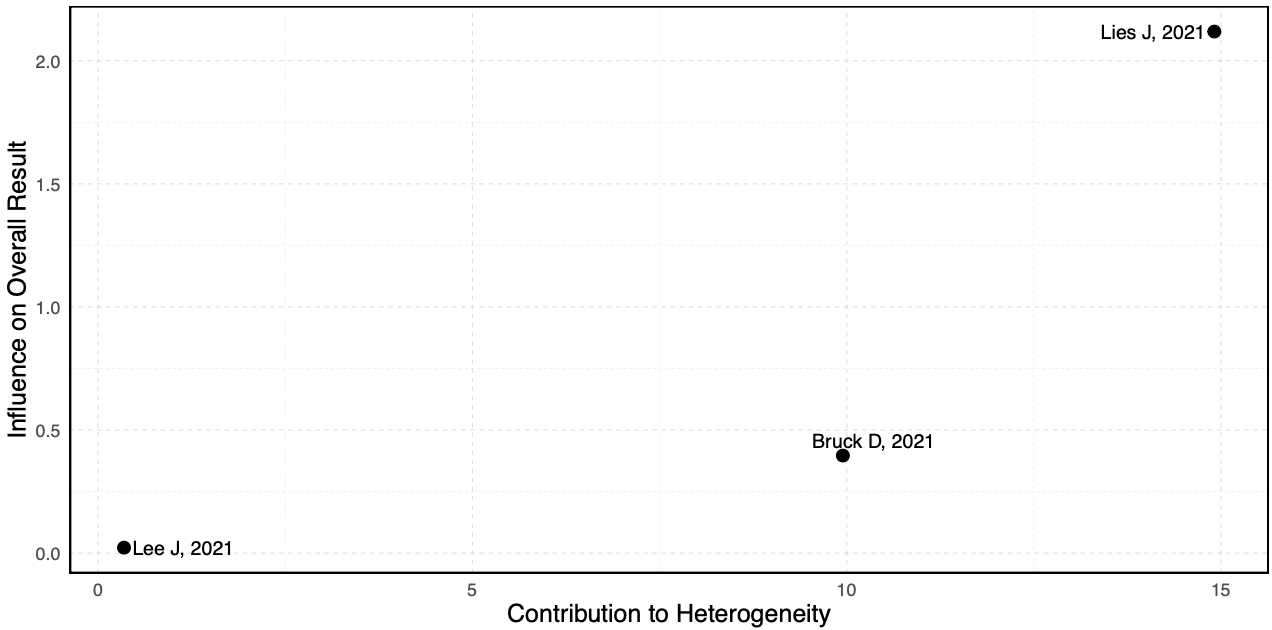


## 7.3. Leave-one-out plot


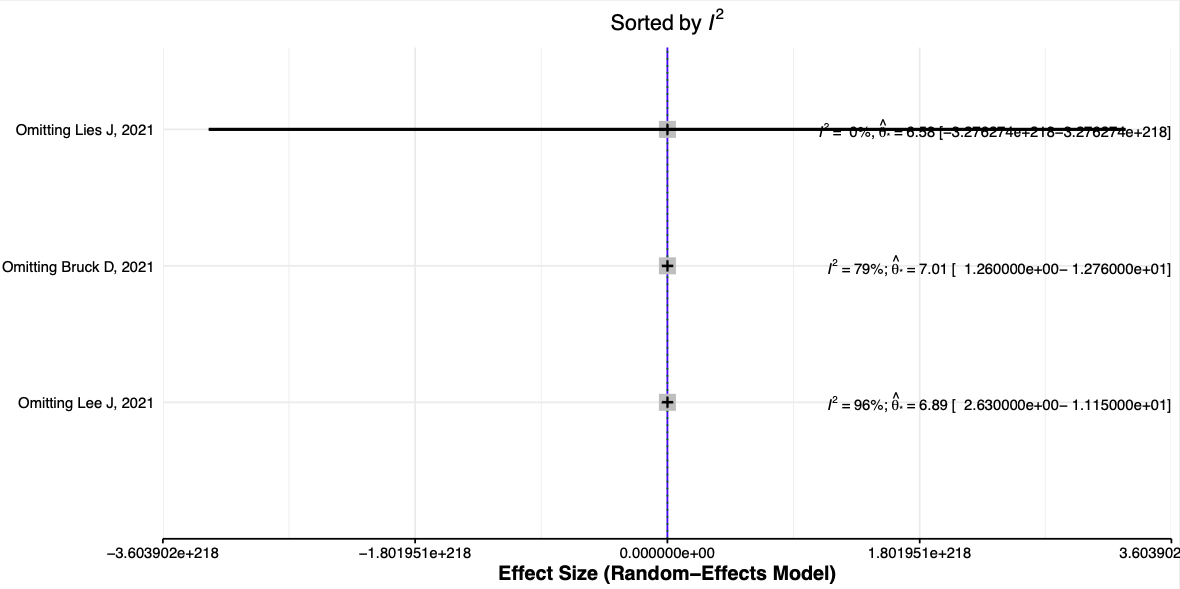


## 7.4. Influence plots


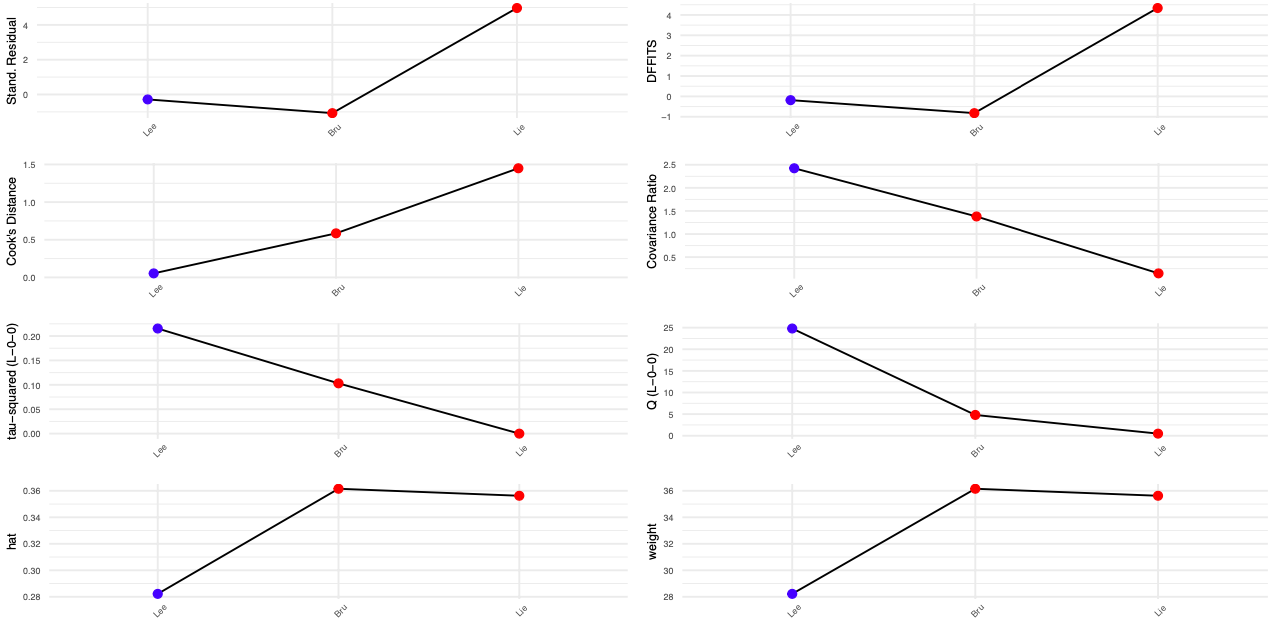


# 8. Sleep Duration Mean in Children and Adolescents

## 8.1. Forest plot


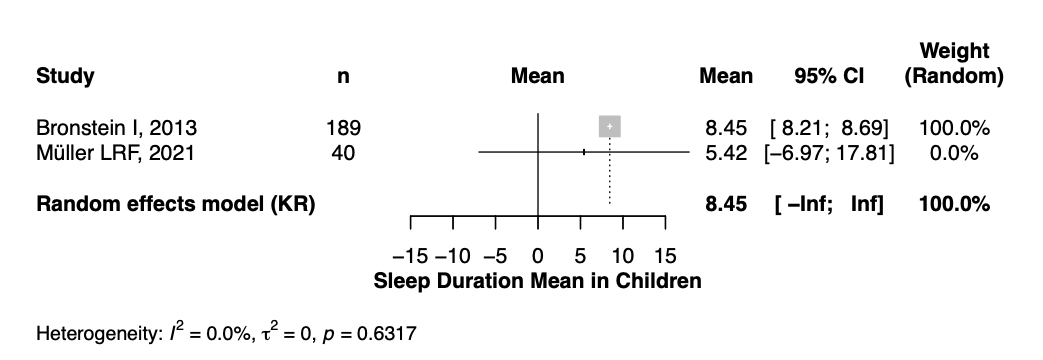


## 8.2. Baujat plot


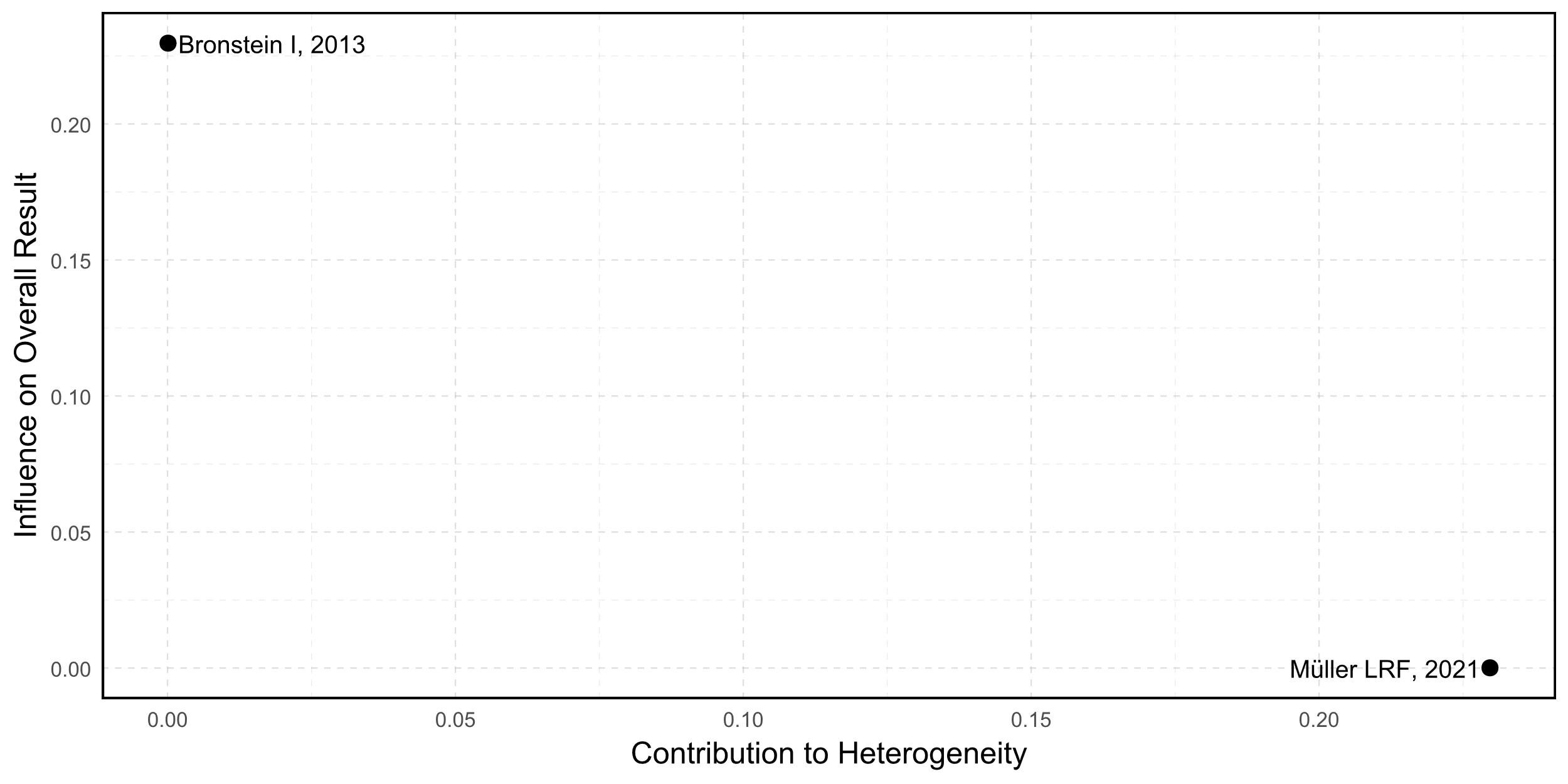


## 8.3. Leave-one-out plot


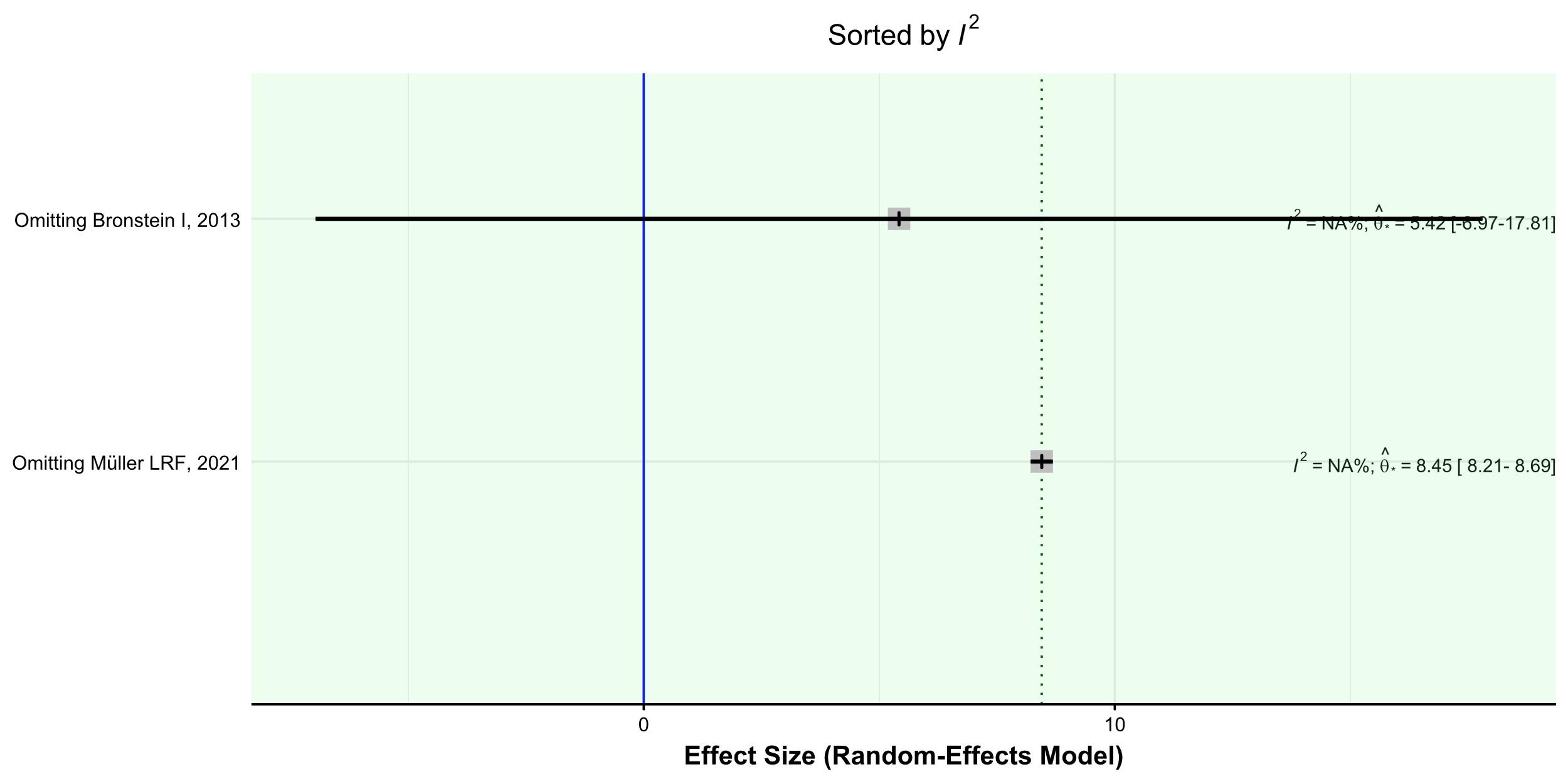


## 8.4. Influence plots


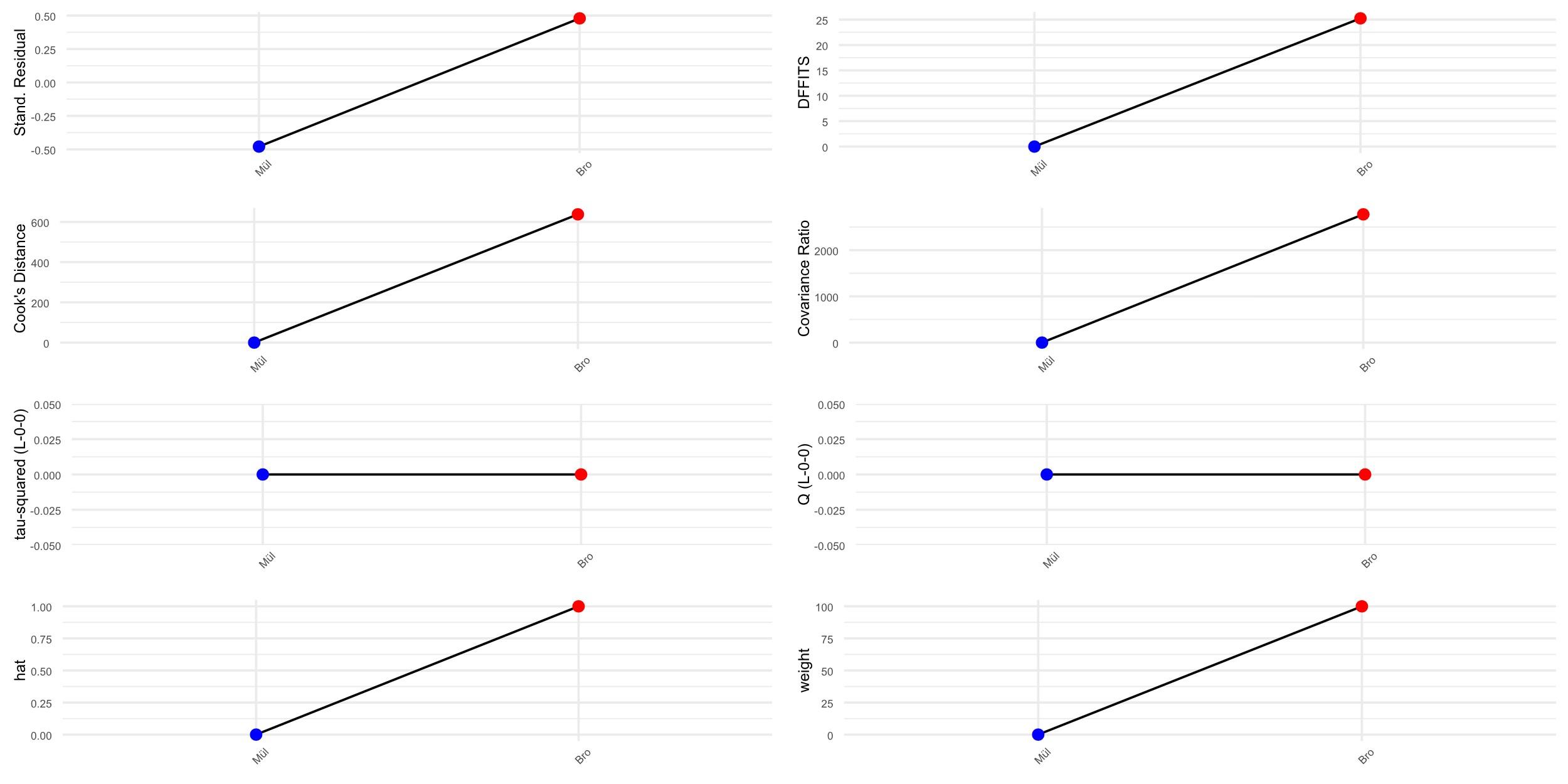


# 9. Sleep Latency Adults

## 9.1. Forest plot


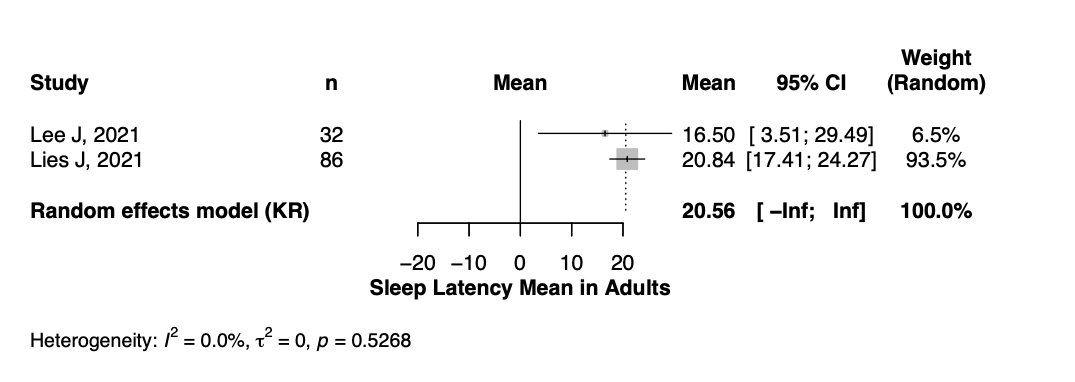


## 9.2. Baujat plot


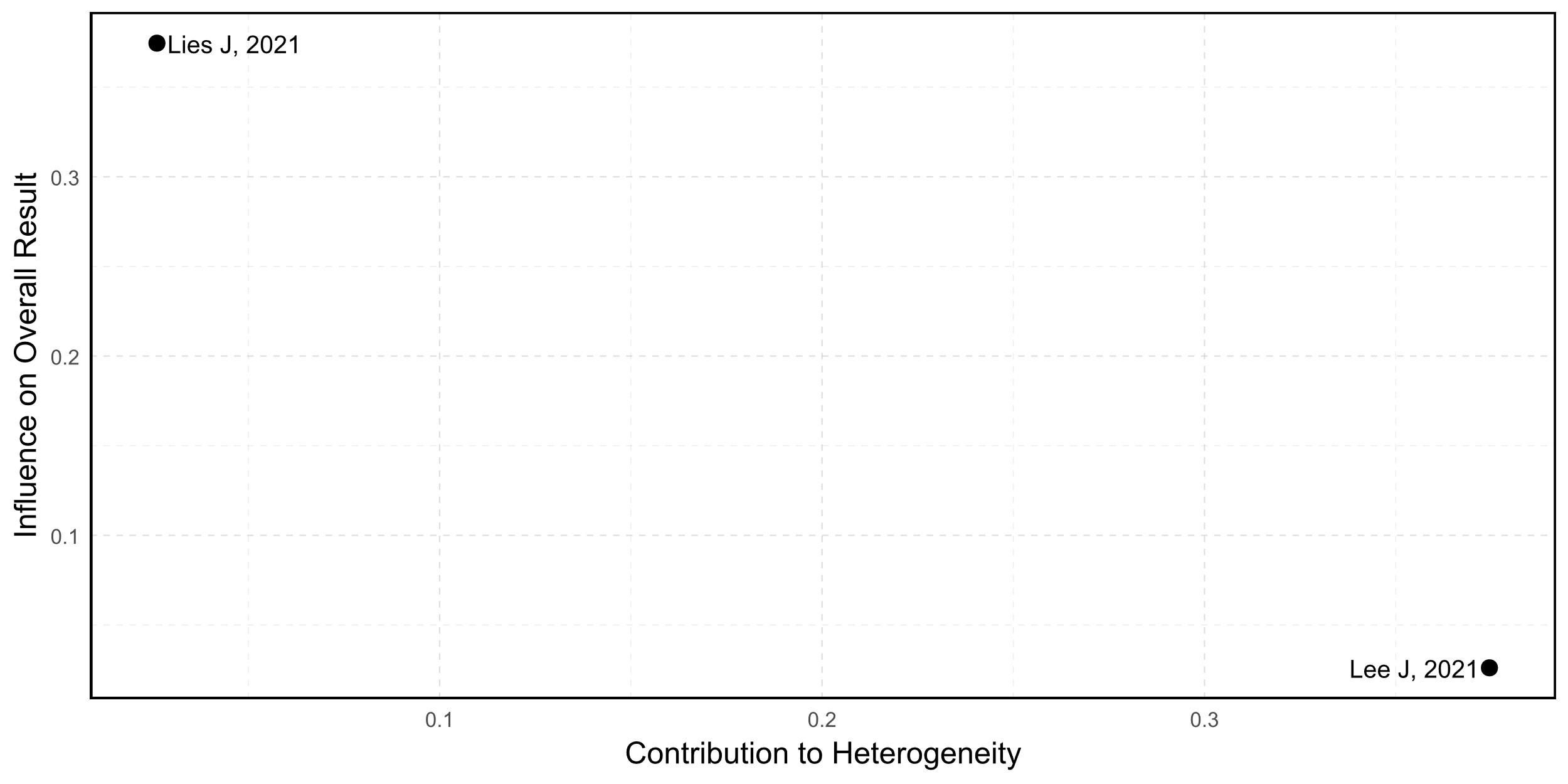


## 9.3. Leave-one-out plot


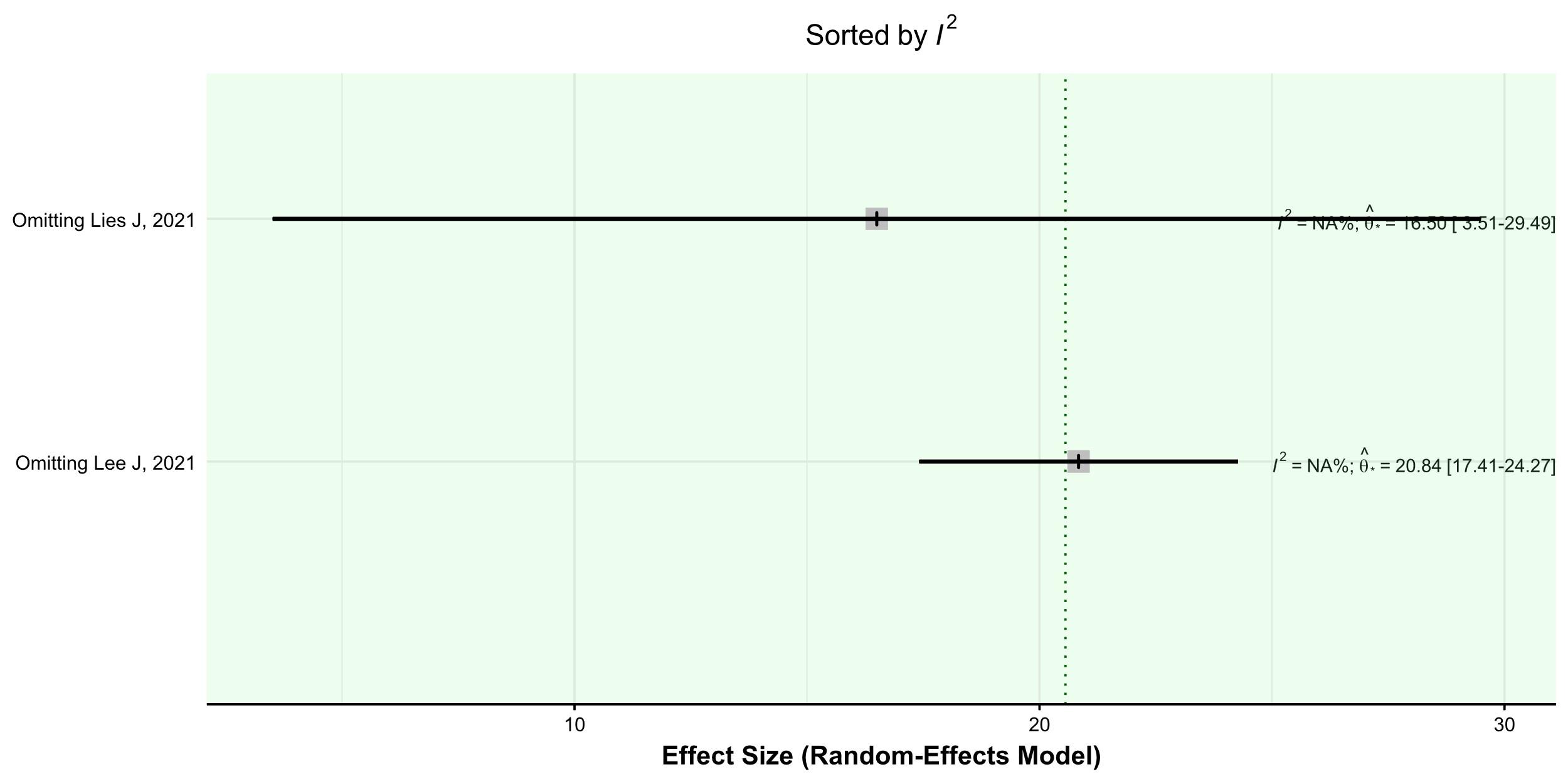


## 9.4. Influence plots


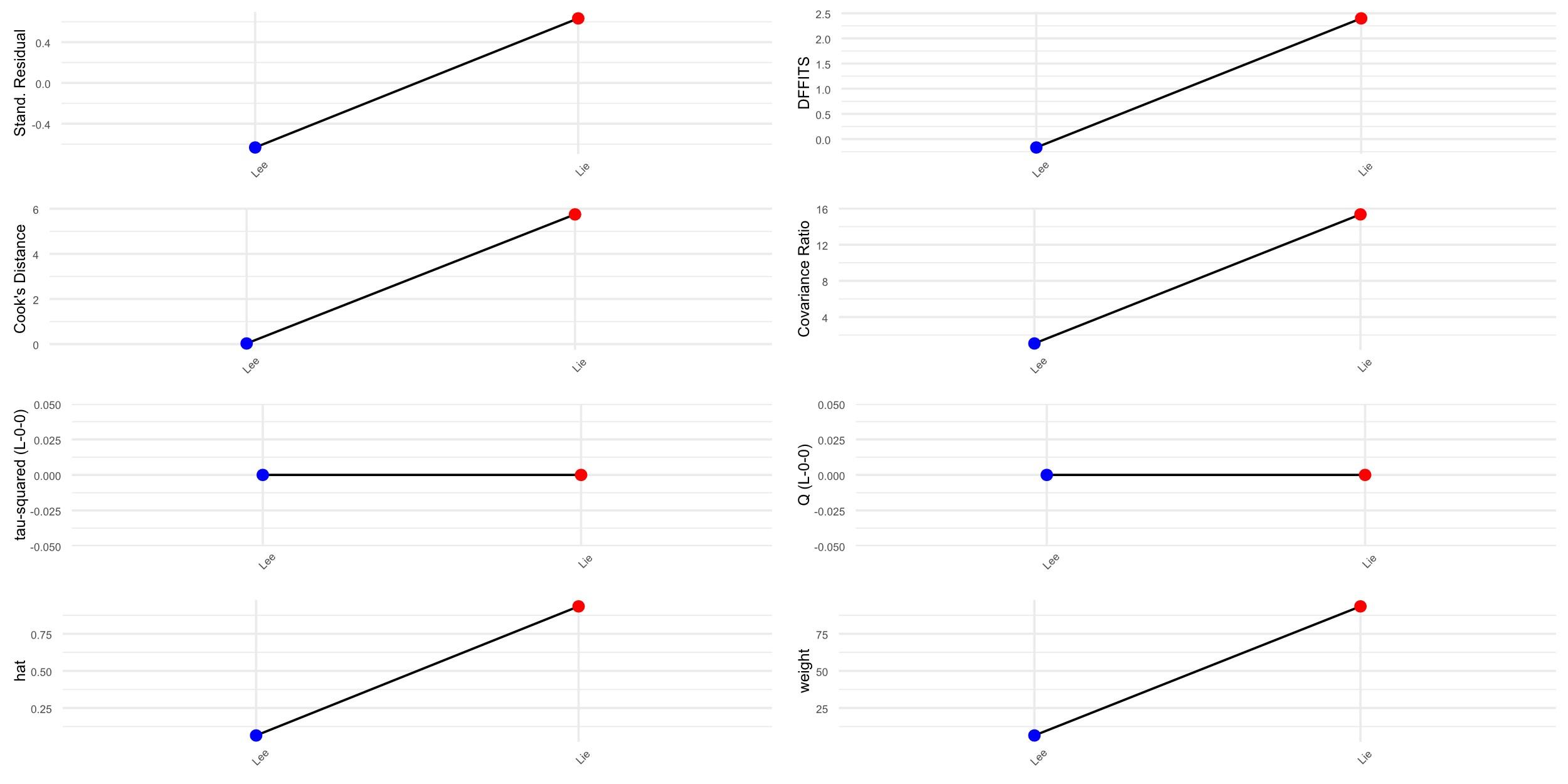


# 10. Sleep Latency Children and Adolescents

## 10.1. Forest plot


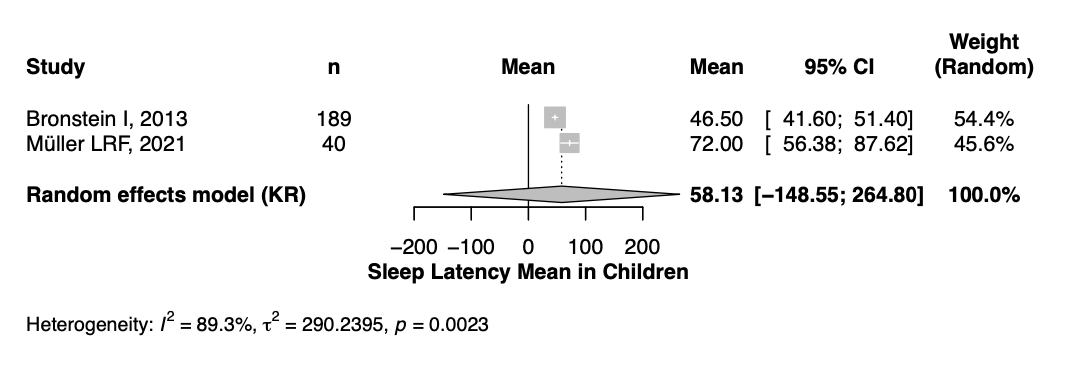


## 10.2. Baujat plot


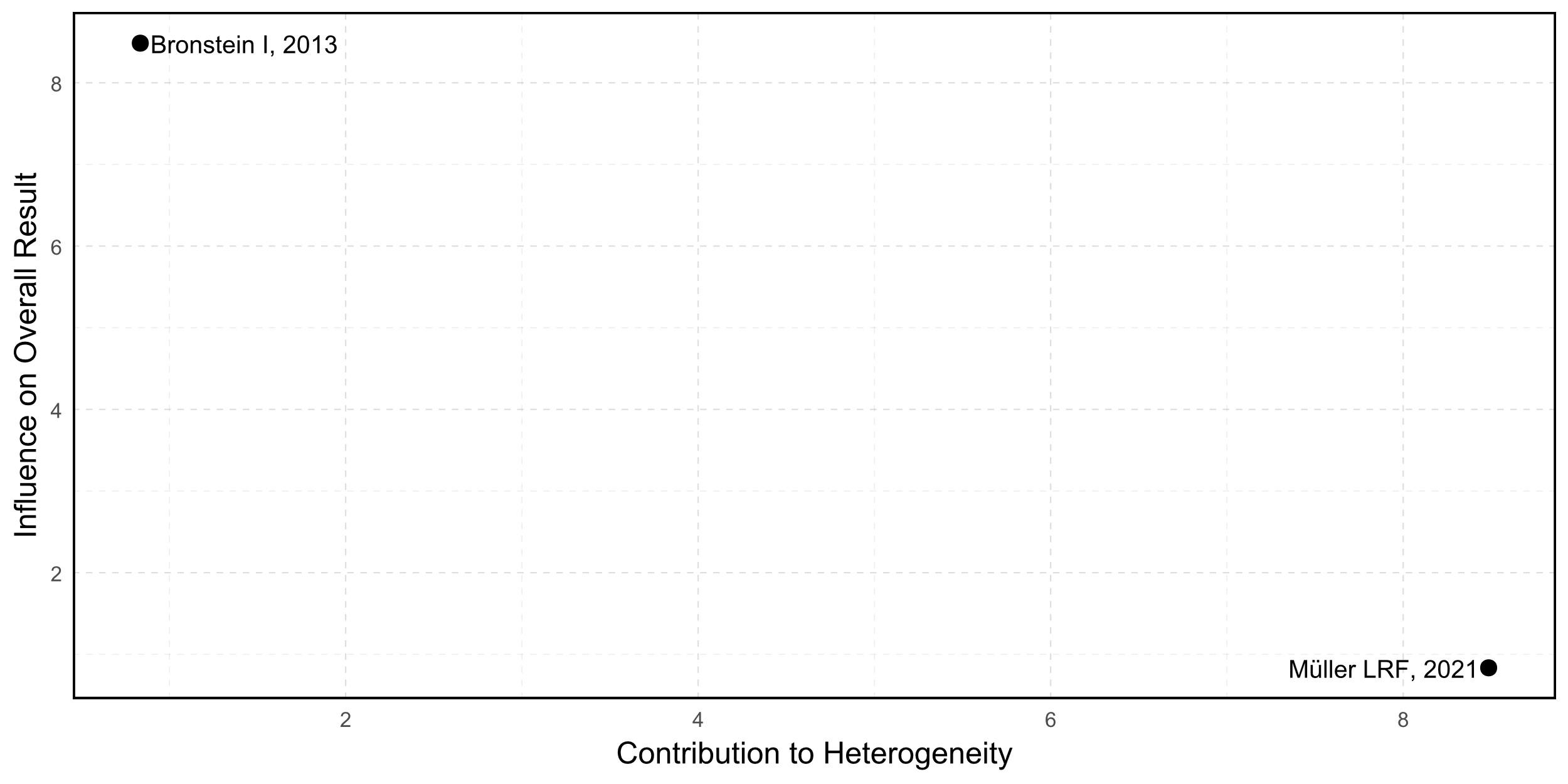


## 10.3. Leave-one-out plot


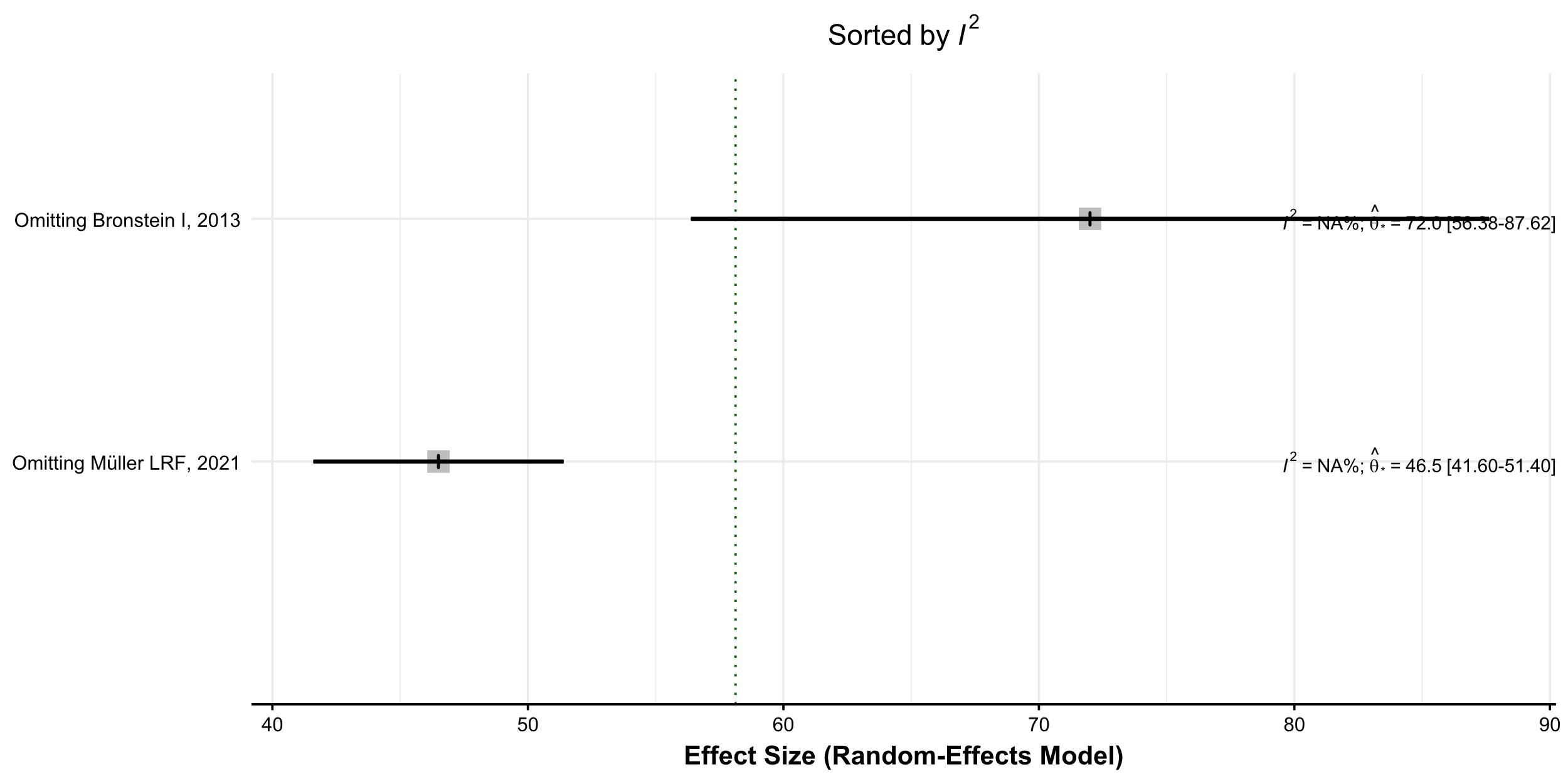


# 11. Sleep Quality 4-Points Likert Scale Mean in Adults

## 11.1. Forest plot


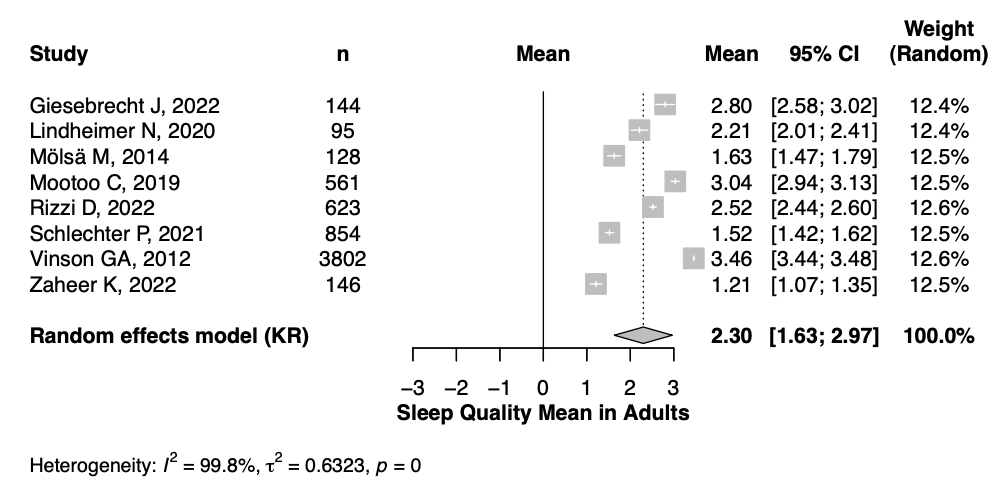


## 11.2. Baujat plot


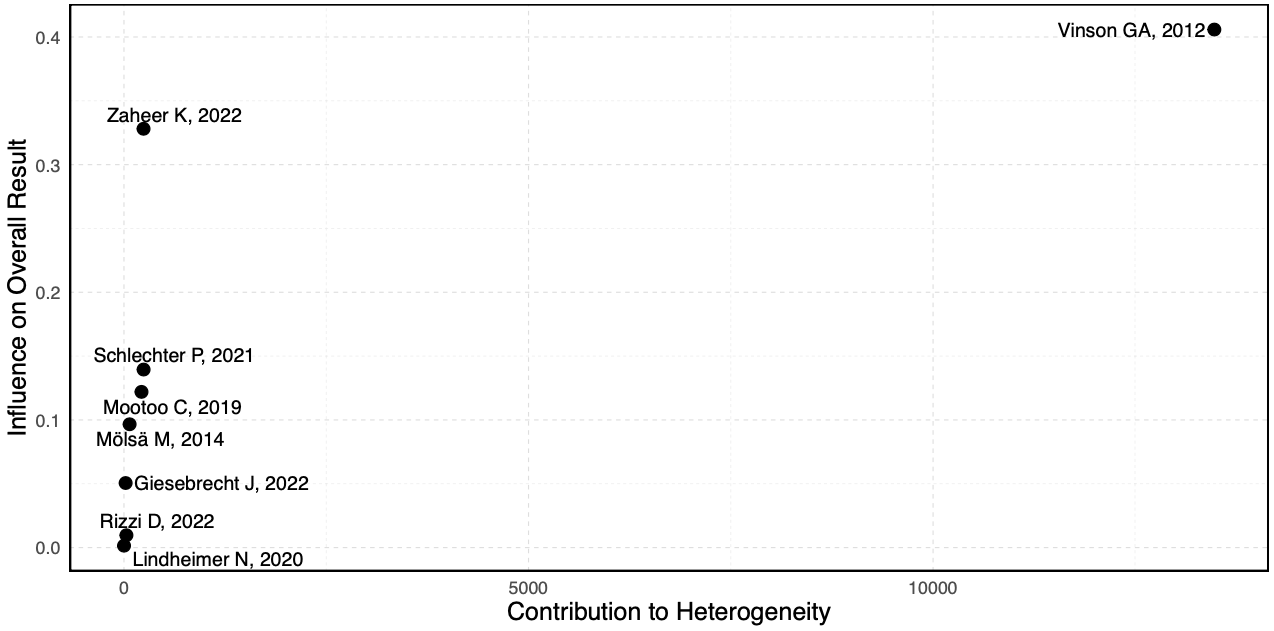


## 11.3. Leave-one-out plot


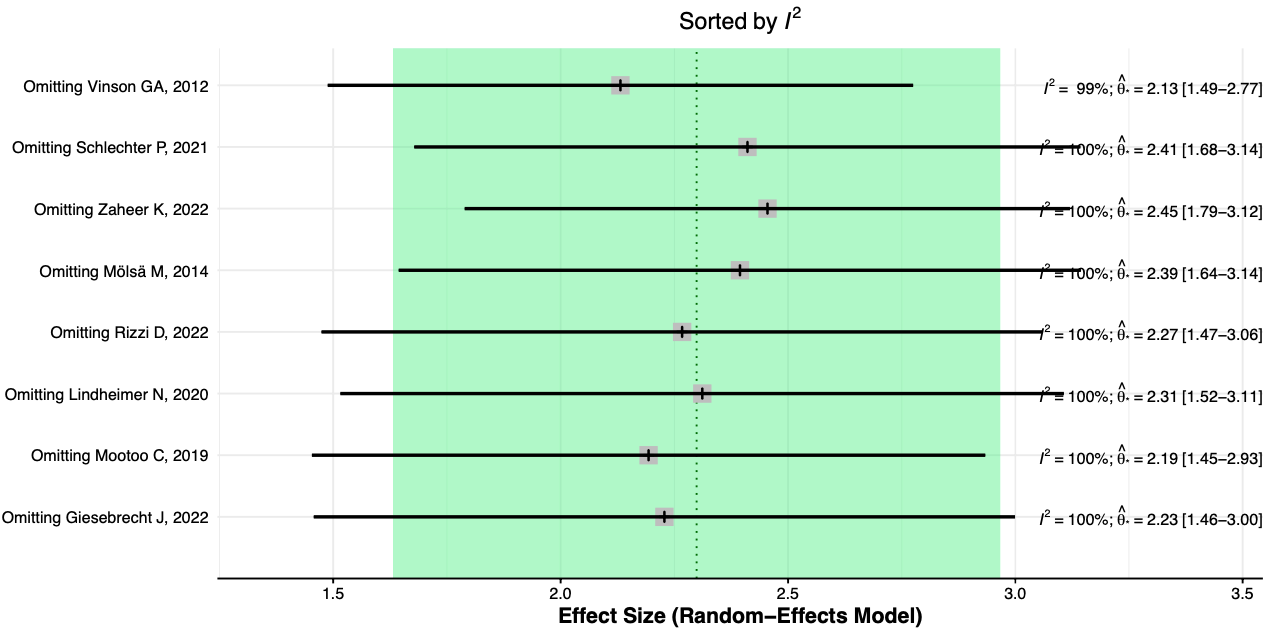


## 11.4. Influence plots


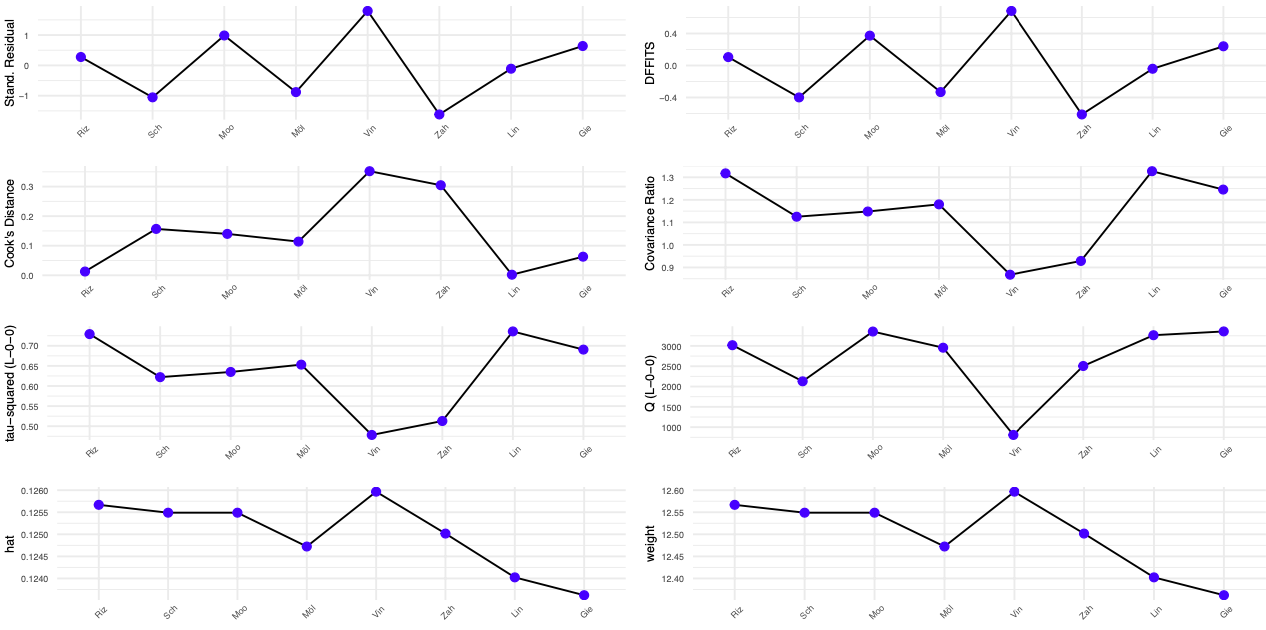


11.5. Funnel plot


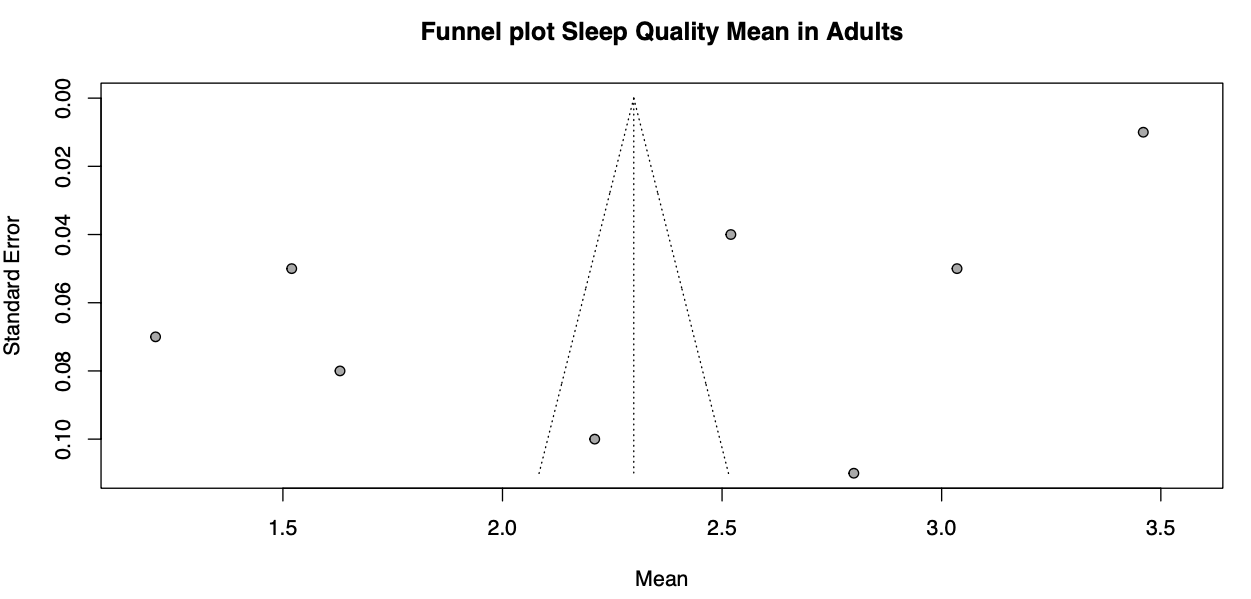


# 12. Sleep Quality 4-Points Likert Scale Mean in Children and Adolescents

## 12.1. Forest plot


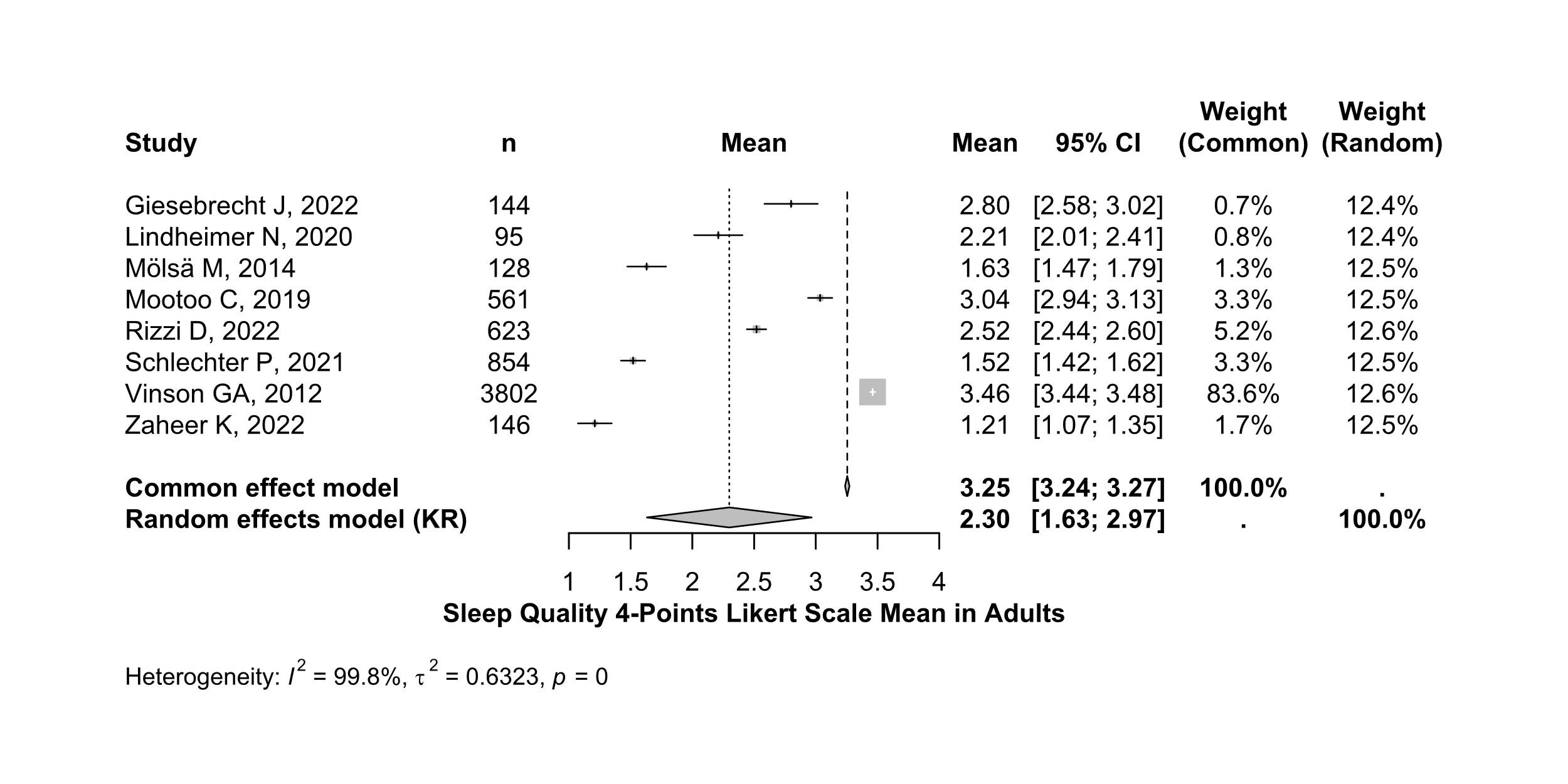


## 12.2. Baujat plot


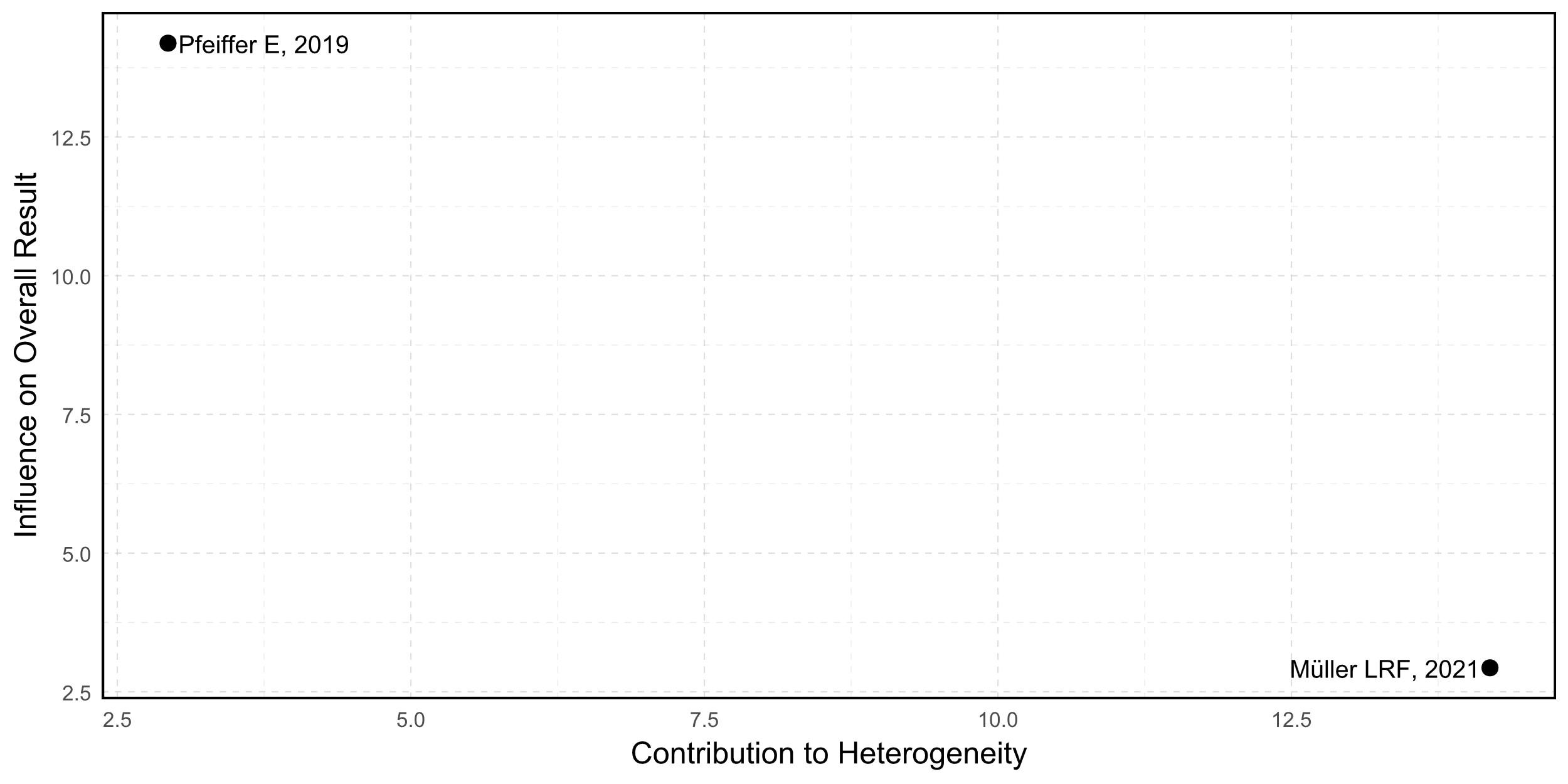


## 12.3. Leave-one-out plot


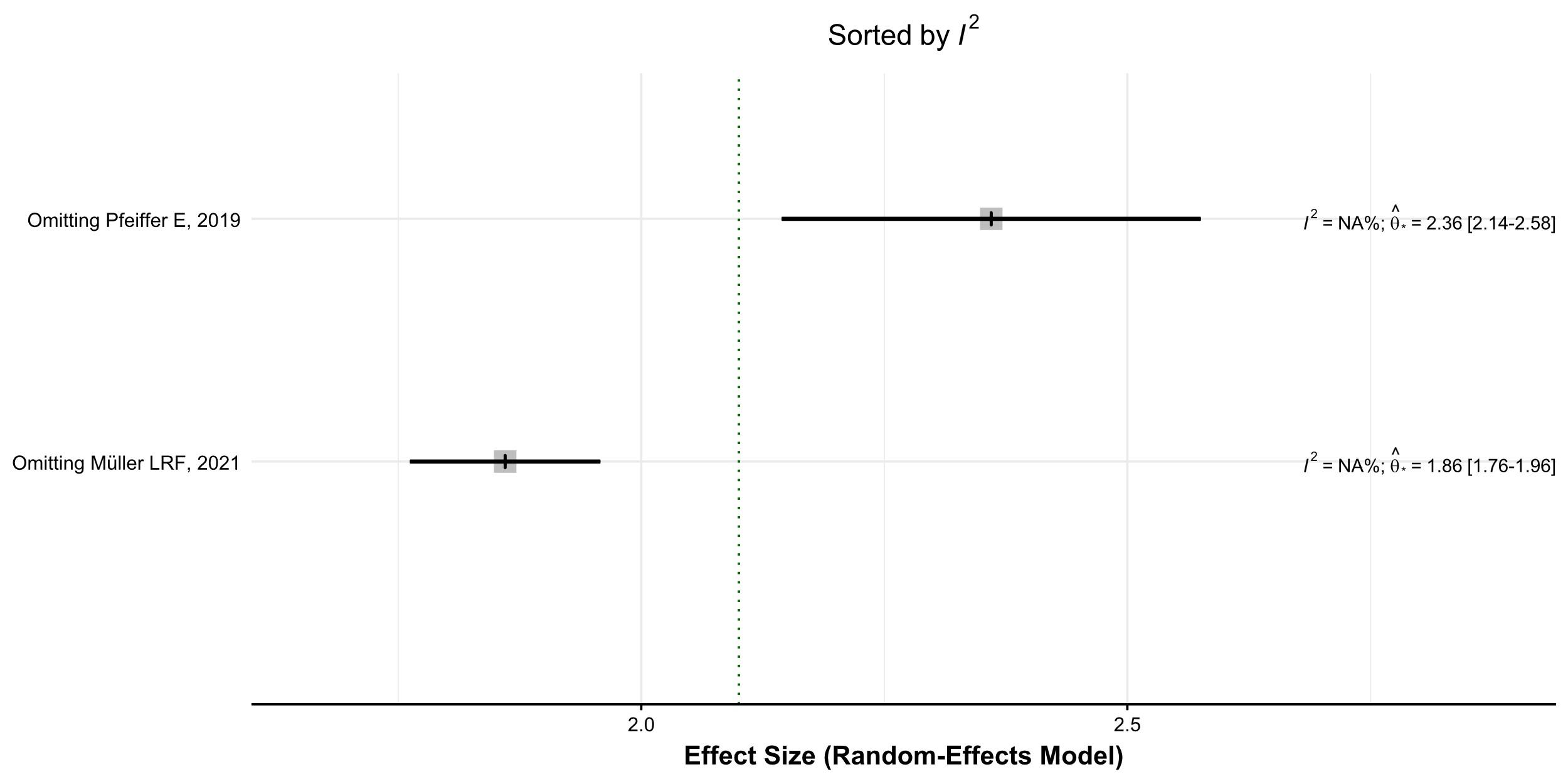


## 12.4. Influence plots


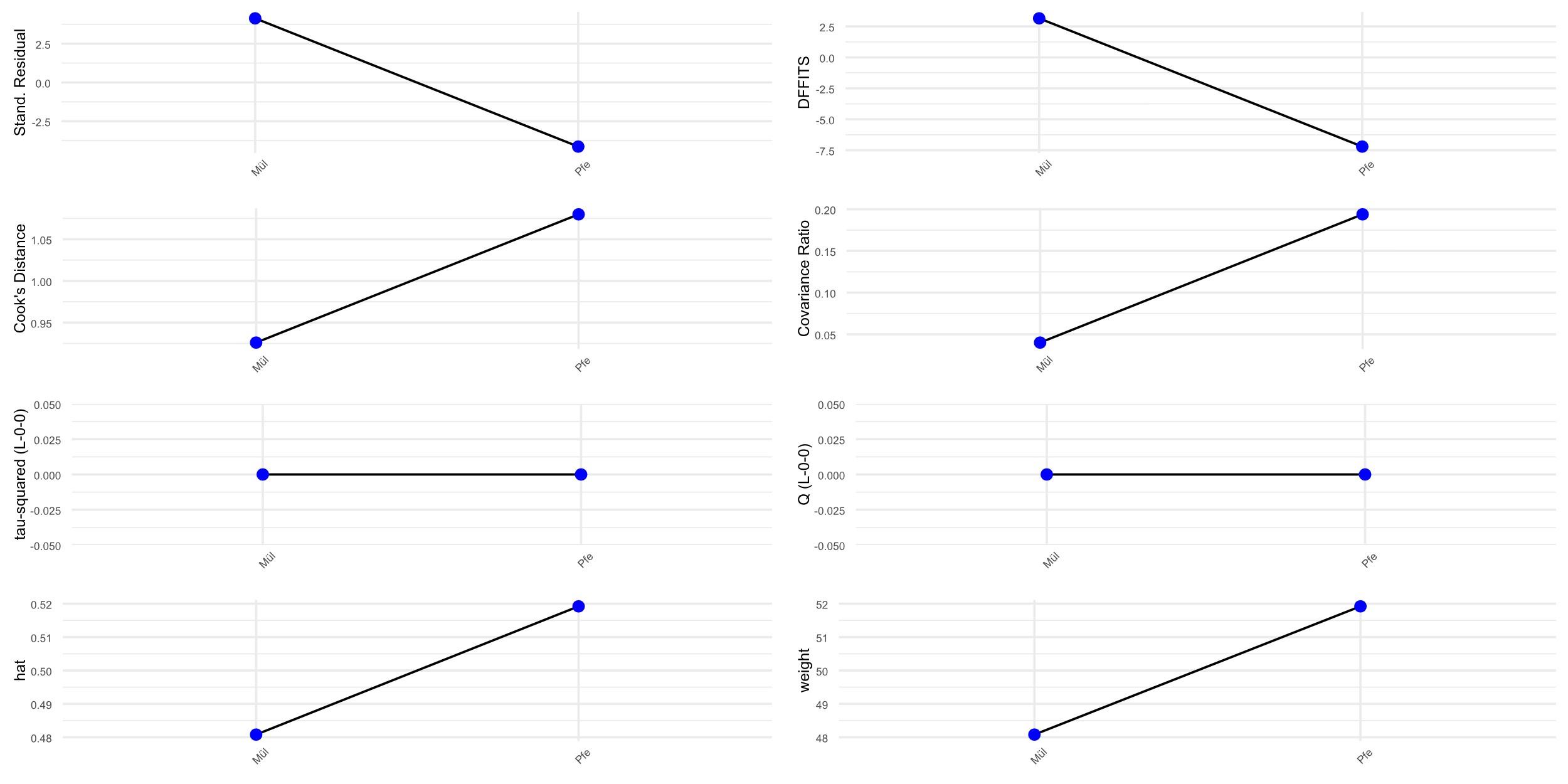


#

14. Supplementary tables

## Table 1. Search strategies.

| PubMed | ("human migration" OR "transients and migrants" OR refugee OR refugees OR "emigration and immigration" OR "emigrants and immigrants" OR "undocumented immigrants" OR "asylum seeker*" OR migrant* OR immigrant* OR immigration* OR emigrant* OR emigration* OR "internal displace*" OR "displaced people" OR "displaced population*" OR "displaced person*" OR "displaced men" OR "displaced individual*" OR "Human Migration"[Mesh] OR "Transients and Migrants"[Mesh] OR "Refugees"[Mesh] OR "Emigration and Immigration"[Mesh] OR "Emigrants and Immigrants"[Mesh] OR "Undocumented Immigrants"[Mesh] OR refugee*) AND (sleep OR nonsleep OR "sleep disorders" OR "sleep disturbances" OR insomnia OR "sleep initiation and maintenance disorders"  OR "disturbed sleep") |
| --- | --- |
| Embase | ('human migration'/exp OR 'human migration' OR 'transients and migrants'/exp OR 'transients and migrants' OR 'refugee'/exp OR refugee OR 'refugees'/exp OR refugees OR 'emigration and immigration'/exp OR 'emigration and immigration' OR 'emigrants and immigrants'/exp OR 'emigrants and immigrants' OR 'undocumented immigrants'/exp OR 'undocumented immigrants' OR 'asylum seeker*' OR migrant* OR immigrant* OR immigration* OR emigrant* OR emigration* OR 'internal displace*' OR 'displaced people' OR 'displaced population*' OR 'displaced person*' OR 'displaced men' OR 'displaced individual*') AND ('sleep'/exp OR sleep OR nonsleep OR 'sleep disorders'/exp OR 'sleep disorders' OR 'sleep disturbances'/exp OR 'sleep disturbances' OR 'insomnia'/exp OR insomnia OR 'sleep initiation and maintenance disorders'/exp OR 'sleep initiation and maintenance disorders' OR 'disturbed sleep') |
| Cochrane  Library | ("human migration" OR "transients and migrants" OR refugee OR refugees OR "emigration and immigration" OR "emigrants and immigrants" OR "undocumented immigrants" OR asylum NEXT seeker* OR migrant* OR immigrant* OR immigration* OR emigrant* OR emigration* OR internal NEXT displace* OR "displaced people" OR displaced NEXT population* OR displaced NEXT person* OR "displaced men" OR displaced NEXT individual*) AND (sleep OR nonsleep OR "sleep disorders" OR "sleep disturbances" OR insomnia OR "sleep initiation and maintenance disorders"  OR "disturbed sleep") |

## Table 2. Newcastle-Ottawa Scale (NOS) for cohort studies.

| Note: A study can be awarded a maximum of one point for each numbered item within the Selection and Outcome categories. A maximum of two points can be given for Comparability  **Selection:**  1) Representativeness of the exposed cohort   1. truly representative of the average in the community (1 point) 2. somewhat representative of the average in the community (1 point) 3. selected group of users eg nurses, volunteers 4. no description of the derivation of the cohort   2) Selection of the non exposed cohort   1. drawn from the same community as the exposed cohort (1 point) 2. drawn from a different source 3. no description of the derivation of the non exposed cohort   3) Ascertainment of exposures   1. secure record (eg surgical records) (1 point) 2. structured interview (1 point) 3. written self report 4. no description   4) Demonstration that outcome of interest was not present at start of study   1. yes (1 point) 2. no |
| --- |
| **Comparability:**  1) Comparability of cohorts on the basis of the design or analysis   1. study controls for (select the most important factor) (1 point) 2. study controls for any additional factor (1 point) (This criteria could be modified to indicate specific control for a second important factor.) |
| **Outcome**:  1) Assessment of outcome   1. independent blind assessment (1 point) 2. record linkage (1 point) 3. self report 4. no description   2) Was follow-up long enough for outcomes to occur   1. yes (select an adequate follow up period for outcome of interest) (1 point) 2. no   3) Adequacy of follow up of cohorts   1. complete follow up - all subjects accounted for (1 point) 2. subjects lost to follow up unlikely to introduce bias - small number lost follow up, or description provided of those lost (1 point) 3. follow up rate inadequate and no description of those lost 4. no statement |

## Table 3. Newcastle-Ottawa Scales (NOS) adapted for cross-sectional studies.

| **Selection:** (Maximum 5 points)  1) Representativeness of the sample:   1. Truly representative of the average in the target population. (all subjects or random sampling) (1 point) 2. Somewhat representative of the average in the target population. (nonrandom sampling) (1 point) 3. Selected group of users. 4. No description of the sampling strategy. 2) Sample size: 5. Justified and satisfactory. (1 point) 6. Not justified.   2) Non-respondents:   1. Comparability between respondents and non-respondents characteristics is established, and the response rate is satisfactory. (1 point) 2. The response rate is unsatisfactory, or the comparability between respondents and non-respondents is unsatisfactory. 3. No description of the response rate or the characteristics of the responders and the non-responders.   3) Ascertainment of the exposure (risk factor):   1. Validated measurement tool. (2 points) 2. Non-validated measurement tool, but the tool is available or described. (1 point) 3. No description of the measurement tool. |
| --- |
| **Comparability**: (Maximum 2 points)  1) The subjects in different outcome groups are comparable, based on the study design or analysis. Confounding factors are controlled.   1. The study controls for the most important factor (select one). (1 point) 2. The study control for any additional factor. (1 point) |
| **Outcome**: (Maximum 3 points)  1) Assessment of the outcome:   1. Independent blind assessment. (2 points) 2. Record linkage. (2 points) 3. Self report. (1 point) 4. No description.   2) Statistical test:   1. The statistical test used to analyze the data is clearly described and appropriate, and the measurement of the association is presented, including confidence intervals and the probability level (p value). (1 point) 2. The statistical test is not appropriate, not described or incomplete. |

## Table 4. Moderators literature gap.

| Study | 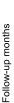 | 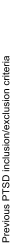 | 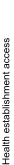 | 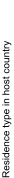 | 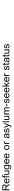 | 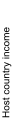 | 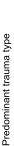 | 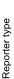 | 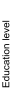 | 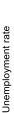 | 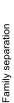 | 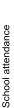 | 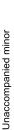 | 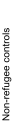 | 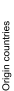 | 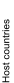 | 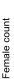 | 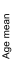 | 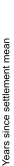 | % |
| --- | --- | --- | --- | --- | --- | --- | --- | --- | --- | --- | --- | --- | --- | --- | --- | --- | --- | --- | --- | --- |
| Spanhel K, 2022 | RP | NA | RP | NR | RP | RP | RP | NA | RP | NR | NR | NA | NA | NA | RP | RP | RP | RP | RP | 58 |
| Sankari S, 2023 | NA | NA | RP | RP | RP | RP | RP | NA | NR | RP | NR | NA | NA | NA | RP | RP | RP | NR | RP | 53 |
| Richter K, 2018 | NA | NA | RP | RP | RP | RP | RP | NA | NR | NR | NR | NA | NA | NA | RP | RP | RP | RP | RP | 53 |
| Özdemir PG, 2021 | NA | NA | RP | NR | RP | RP | RP | NA | RP | RP | NR | NA | NA | NA | RP | RP | RP | RP | NR | 53 |
| Lee J, 2021 | NA | NA | NR | NR | RP | RP | RP | NA | NR | NR | RP | NA | NA | RP | RP | RP | RP | RP | RP | 53 |
| Sandahl H, 2020 | RP | RP | RP | NR | RP | RP | RP | NA | RP | RP | NR | NA | NA | NA | RP | RP | RP | RP | RP | 68 |
| Schumm H, 2023 | NA | RP | RP | RP | RP | RP | NR | NA | RP | RP | NR | NA | NA | NA | RP | RP | RP | RP | RP | 63 |
| Bruck D, 2021 | NA | NA | NR | RP | RP | RP | RP | NA | RP | RP | NR | NA | NA | RP | RP | RP | RP | RP | RP | 63 |
| Lies J, 2021 | NA | NA | NR | RP | RP | RP | RP | NA | RP | RP | NR | NA | NA | NA | RP | RP | RP | RP | RP | 58 |
| Al-Smadi AM, 2019 | NA | NA | RP | RP | RP | RP | RP | NA | RP | RP | NR | NA | NA | NA | RP | RP | RP | RP | NR | 58 |
| Meurling J, 2023 | NA | NA | NR | NR | RP | RP | RP | NA | RP | NR | NR | NA | NA | NA | RP | RP | RP | RP | RP | 47 |
| Park J, 2019 | NA | NA | NR | NR | RP | RP | RP | NA | NR | NR | NR | RP | NA | NA | RP | RP | RP | RP | NR | 42 |
| Gammoh OS, 2024a | NA | NA | RP | RP | RP | RP | RP | NA | RP | RP | NR | NA | NA | NA | RP | RP | RP | NR | RP | 58 |
| Lies J, 2019 | NA | NA | RP | RP | RP | RP | RP | RP | NA | NA | NR | NR | NR | NA | RP | RP | RP | RP | RP | 58 |
| Carlsson JM, 2006 | RP | NA | RP | NR | RP | RP | RP | NA | RP | RP | NR | NA | NA | NA | RP | RP | RP | RP | RP | 63 |
| Aldukhail S, 2023 | NR | NA | RP | RP | RP | RP | RP | RP | RP | RP | NR | NR | NR | NA | RP | RP | RP | RP | NR | 63 |
| Rizzi D, 2022 | NA | NA | RP | RP | RP | RP | RP | NA | NR | NR | RP | NA | NA | NA | RP | RP | RP | RP | NR | 53 |
| Parvez A, 2023 | RP | NA | RP | RP | RP | RP | RP | NA | RP | RP | NR | NA | NA | NA | RP | RP | RP | RP | RP | 68 |

| Trohl U, 2021 | RP | NA | RP | RP | RP | RP | RP | NA | NR | NR | RP | NA | NA | NA | RP | RP | RP | RP | RP | 63 |
| --- | --- | --- | --- | --- | --- | --- | --- | --- | --- | --- | --- | --- | --- | --- | --- | --- | --- | --- | --- | --- |
| Tay AK, 2015 | NA | NA | NR | RP | RP | RP | RP | NA | RP | RP | RP | NA | NA | NA | RP | RP | RP | RP | RP | 63 |
| Tamblyn JM, 2011 | RP | NA | RP | RP | RP | RP | RP | NA | NR | NR | NR | NA | NA | NA | RP | RP | RP | RP | RP | 58 |
| Loutan L, 1999 | NA | NA | RP | NR | RP | RP | RP | NA | RP | NR | RP | NA | NA | NA | RP | RP | RP | RP | NR | 53 |
| Lee YG, 2016 | NA | NA | NR | NR | RP | RP | RP | NA | NR | NR | NR | NA | NA | RP | RP | RP | RP | RP | RP | 47 |
| Honkala E, 1992 | NA | NA | RP | RP | RP | RP | RP | RP | NA | NA | NR | NR | NR | NA | RP | RP | RP | RP | NR | 53 |
| Westermeyer JJ, 2010 | NA | NA | NR | RP | RP | RP | RP | NA | NR | NR | NR | NA | NA | NA | RP | RP | RP | RP | RP | 47 |
| Gulden A, 2010 | NA | NA | NR | RP | RP | RP | RP | NA | RP | NR | NR | NA | NA | NA | RP | RP | RP | RP | RP | 53 |
| Gowin M, 2017 | NA | NA | NR | NR | RP | RP | RP | NA | RP | NR | RP | NA | NA | NA | RP | RP | NA | RP | NR | 42 |
| Schlechter P, 2021 | NA | NA | NR | NR | RP | RP | RP | NA | NR | NR | NR | NA | NA | NA | RP | RP | RP | RP | NR | 37 |
| Mootoo C, 2019 | NA | NA | RP | RP | RP | RP | RP | NA | NR | NR | NR | NA | NA | NA | RP | RP | RP | RP | NR | 47 |
| Schnyder U, 2015 | NA | NA | RP | NR | RP | RP | RP | NA | RP | NR | NR | NA | NA | NA | RP | RP | RP | RP | RP | 53 |
| Weaver TL, 2008 | NA | NA | RP | NR | RP | RP | RP | NA | RP | RP | NR | NA | NA | NA | RP | RP | RP | RP | RP | 58 |
| Mölsä M, 2014 | NA | NA | RP | RP | RP | RP | RP | NA | RP | RP | NR | NA | NA | RP | RP | RP | RP | RP | RP | 68 |
| Vinson GA, 2012 | NA | NA | RP | RP | RP | RP | RP | NA | RP | NR | RP | NA | NA | NA | RP | RP | RP | RP | NR | 58 |
| Zaheer K, 2022 | NA | NA | RP | RP | RP | RP | RP | NA | RP | NR | NR | NA | NA | NA | RP | RP | RP | RP | NR | 53 |
| Lindheimer N, 2020 | NA | NA | RP | RP | RP | RP | RP | NA | RP | NR | NR | NA | NA | NA | RP | RP | RP | RP | NR | 53 |
| Giesebrecht J, 2022 | NA | NA | RP | RP | RP | RP | RP | NA | RP | NR | NR | NA | NA | NA | RP | RP | RP | RP | RP | 58 |
| Abuali M, 2024 | NA | NA | RP | RP | RP | RP | RP | RP | RP | NA | RP | NR | NR | NA | RP | RP | RP | RP | NR | 63 |
| Montgomery E, 2001 | NA | NA | NR | RP | RP | RP | RP | RP | NA | NA | RP | NR | NR | NA | RP | RP | RP | RP | NR | 53 |
| Pfeiffer E, 2019 | NA | NA | RP | NR | RP | RP | RP | RP | NA | NA | RP | NR | RP | NA | RP | RP | RP | RP | RP | 63 |
| Genton PC, 2019 | NA | NA | RP | NR | RP | RP | RP | RP | NA | NA | RP | NR | RP | NA | RP | RP | RP | RP | NR | 58 |
| Ceri V, 2016 | NA | NA | RP | RP | RP | RP | RP | RP | NA | NA | RP | NR | NR | NA | RP | RP | RP | RP | NR | 58 |
| Eiset AH, 2020 | NA | NA | RP | RP | RP | RP | RP | RP | NA | NA | RP | NR | RP | NA | RP | RP | RP | RP | NR | 63 |
| Schumacher L, 2021 | NR | NA | RP | NR | RP | RP | RP | RP | NA | NA | RP | NR | RP | NA | RP | RP | RP | RP | RP | 63 |
| Hjern A, 2019 | NA | NA | RP | RP | RP | RP | RP | RP | NA | NA | RP | RP | RP | NA | RP | RP | RP | RP | NR | 68 |
| Hjern A, 1991 | NR | NA | RP | NR | RP | RP | RP | RP | NA | NA | RP | NR | RP | NA | RP | RP | RP | RP | NR | 58 |
| Nasıroğlu S, 2018 | NA | NA | RP | RP | RP | RP | RP | RP | NA | NA | NA | RP | RP | NA | RP | RP | RP | RP | NR | 63 |
| Husni M, 2001 | NA | NA | NR | NR | RP | RP | RP | NA | RP | RP | NR | NA | NA | NA | RP | RP | RP | RP | RP | 53 |
| Hinton DE, 2009 | NA | RP | RP | RP | RP | RP | RP | NA | RP | NR | RP | NA | NA | NA | RP | RP | RP | RP | NR | 63 |

| Cernovsky Z, 1988 | NA | NA | NR | RP | RP | RP | RP | NA | RP | RP | RP | NA | NA | NA | RP | RP | RP | RP | NR | 58 |
| --- | --- | --- | --- | --- | --- | --- | --- | --- | --- | --- | --- | --- | --- | --- | --- | --- | --- | --- | --- | --- |
| Lee S, 2021 | NA | NA | RP | RP | RP | RP | RP | NA | NA | NR | NA | NA | NA | NA | RP | RP | RP | RP | NR | 47 |
| Berkson SY, 2014 | NR | NA | RP | RP | RP | RP | RP | NA | RP | NR | NA | NA | NA | NA | RP | RP | RP | RP | NR | 53 |
| Bronstein I, 2013 | NA | NA | NR | RP | RP | RP | RP | RP | NA | NA | RP | NR | RP | NA | RP | RP | RP | RP | RP | 63 |
| Simich L, 2006 | NA | NA | NR | RP | RP | RP | RP | NA | RP | RP | RP | NA | NA | NA | RP | RP | RP | RP | RP | 63 |
| Müller LRF, 2021 | NR | NA | RP | RP | RP | RP | RP | NR | NA | NA | RP | RP | NR | NA | RP | RP | RP | RP | RP | 63 |
| Mangrio E, 2020 | NA | NA | RP | NR | RP | RP | RP | NA | RP | NR | NR | NA | NA | NA | RP | RP | RP | RP | NR | 47 |
| Ku SY, 2006 | NA | NA | RP | RP | RP | RP | NR | NA | RP | NR | NR | NA | NA | NA | RP | RP | RP | RP | NR | 47 |
| Knappe F, 2023 | NR | NA | RP | RP | RP | RP | RP | NA | RP | RP | RP | NA | NA | NA | RP | RP | RP | RP | RP | 68 |
| Itani T, 2017 | NA | NA | RP | RP | RP | RP | RP | RP | NA | NA | NR | RP | NR | NA | RP | RP | RP | NA | NR | 53 |
| Hinton DE, 2015 | NA | NA | RP | RP | RP | RP | RP | NA | NR | NR | NR | NA | NA | NA | RP | RP | RP | NA | NR | 42 |
| Hinton DE, 2005 | NA | NA | RP | RP | RP | RP | RP | NA | NR | NR | NR | NA | NA | NA | RP | RP | RP | RP | NR | 47 |
| Gammoh OS, 2024b | NA | NA | RP | RP | RP | RP | RP | NA | RP | RP | NR | NA | NA | NA | RP | RP | RP | NA | NR | 53 |
| Boiko DI, 2024 | NA | NA | NR | NR | RP | NR | RP | NA | NR | RP | NR | NA | NA | RP | RP | RP | RP | RP | NR | 42 |
| Thabet AA, 1999 | NA | NA | NR | RP | RP | NR | RP | RP | NA | NA | NR | RP | NR | NA | RP | RP | RP | RP | NR | 47 |
| Kinzie JD, 1986 | NA | NA | RP | NR | RP | RP | RP | RP | NA | NA | RP | RP | RP | NA | RP | RP | RP | RP | RP | 68 |
| Realmuto GM, 1992 | NA | NA | NR | NR | RP | RP | RP | RP | NA | RP | RP | RP | NR | NA | RP | RP | RP | RP | NR | 58 |
| Gammoh OS, 2024c | NA | NA | RP | RP | RP | RP | RP | NA | RP | RP | RP | NA | NA | NA | RP | RP | RP | RP | NR | 63 |
| % | 9 | 5 | 71 | 67 | 100 | 97 | 97 | 27 | 52 | 33 | 38 | 12 | 14 | 8 | 100 | 100 | 98 | 92 | 47 |  |
| Note: NA = "Not applicable", NR = "Not reported", RP="Reported" | | | | | | | | | |  |  |  |  |  |  |  |  |  |  |  |

## Table 5. Terminology and definitions.

| Terminology | Description category | Definition |
| --- | --- | --- |
| Sleep adversities | Broad description on impaired sleep homeostasis grouping multiple  phenomenol  ogical instances | Abuali, M. (2014): Reported 'poor sleep' in a clinical interview according to the CDC domestic refugee guidance (p. 1) |
|  |  | Ceri, V. (2016): Answered "yes" to having "sleep problems", such as problems falling asleep, frequent awakenings, and parasomnias like somnambulism and nightmares |
|  |  | Eiset, A.H. (2020): Answered "yes" to having "sleep problems", without further detail |
|  |  | Giesebrecht, J. (2022): Answered "several days", "more than half the days" or "nearly every day"to having "trouble sleeping" according to PHQ-9 item |
|  |  | Gowin, M. (2017): Said to be diagnosed "by a professional", without further detail |
|  |  | Gulden, A. (2010): Answered "yes" to having "sleep problems", such as insomnia, nightmares, daytime sleepiness |
|  |  | Hjern, A. (2019): Answered "yes" to having "sleeping problems"; to be recorded as yes, mental health problems, including sleeping disturbances, had to be severe enough that the nurse judged them to impair the well-being of the child on a daily basis. |
|  |  | Kinzie, J.D. (1986): Reported "trouble sleeping" du |
|  |  | Lee, Y.G. (2016): According to ICD-10; participants experiencing at least one of the three types of insomnia (initial, maintenance, and terminal) ≥3/week were classified as having significant insomnia. |
|  |  | Mangrio, E. (2020): Answered "bad" to the questio |
|  |  | Nasıroğlu, S. (2018): Said to be diagnosed "by a professional", without further detail |
|  |  | Parvez, A. (2023): According to ICD-10; "listed as having the diagnosis if made by the primary care physician" |

|  |  | Realmuto, G.M. (1992): Answered "yes" to the item of Sleep Disturbance of an adapted version of the CPTSD-RI scale |
| --- | --- | --- |
|  |  | Richter, K. (2018): According to ICD-10; tried to establish the difference between insomnia symptoms and disease; the subject reported that their insomnia bothered them much more than the depressive or anxious mood and that better sleep would also improve their mood |
|  |  | Schumacher, L. (2021): Answered "yes" to having "sleep problems", such as disrupted sleep patterns or insomnia that occur as part of the arousal symptoms following a traumatic event |
|  |  | Tamblyn, J.M. (2011): Diagnostic according to DSM-IV TR criteria, by the RMSC licensed professional counselor, licensed clinical social worker, or psychiatrist; "insomnia as persistent difficulty with falling or staying asleep despite trying to do so" |
|  |  | Tay, A.K. (2015): Yes or no to insomnia-related items according to DSM-IV and 5 |
|  |  | Thabet, A.A. (1999): Answered "few", "some", "frequent" or "most of the time" to the item D1 (Sleep Disturbance) of the scale CPTSD-RI |
|  |  | Trohl, U. (2021): Noted as a symptom complaint; "in the course of the assessment interview, the psychologist specifically asked for ‘disorder-relevant psychopathological symptoms'" |
|  |  | Westermeyer, J.J. (2010): Answered "yes" to having "sleep problems", such as insomnia, nightmares, daytime sleepiness |
|  |  | Zaheer, K. (2022): Answered "very often", "occasionally" and "rarely" to having "problems sleeping" according to Oral Health and  Well-Being questionnaire item from the American Dental Association |
|  | Clinical insomnia as per validated scale cut-offs | Al-Smadi, A.M. (2019): Defined as ISI>14 |
|  |  | Boiko, D.I. (2024): Defined as ISI>14 |
|  |  | Bruck, D. (2021): Defined as ISI>14 |
|  |  | Gammoh, O.S. (2024a): Defined as "cutoff score of >14 indicates severe insomnia symptoms" |
|  |  | Gammoh, O.S. (2024b): Defined as "cutoff score of >14 indicates severe insomnia symptoms" |
|  |  | Gammoh, O.S. (2024c): Defined as "cutoff score of >14 indicates severe insomnia symptoms" |
|  |  | Knappe, F. (2023): Defined as "cutoff score of >14 indicates severe insomnia symptoms" |
|  |  | Lies, J. (2021): Defined as ISI>14; estimated assuming normal distribution as raw data wasn't available through contact |
|  |  | Meurling, J. (2023): Defined as ISI>14 |
|  |  | Park, J. (2019): Probably as ISI>14; the study claimed that 14 subjects surpassed the "cut-off" score, which commonly is 14 points |
|  |  | Spanhel, K. (2022): At baseline, defined as ISI>14 |
|  | Disorders of initiating or maintaining sleep | Aldukhail, S. (2023): Answered "yes" to difficulty staying asleep |
|  |  | Carlsson, J.M. (2006): Baseline; rating to insomnia-related item of HTQ based on DSM-III-R |
|  |  | Genton, P.C. (2019): Diagnosed accordingly to ICD-10 |
|  |  | Hjern, A. (1991): Answered "yes" to having “nightmares at least twice a week, night terrors at least once a month, often waking up and not being able to fall asleep without parental assistance and/or often having difficulties of going to sleep at night." |
|  |  | Honkala, E. (1992): Answered "yes" to "waking up during the night without any reason" |
|  |  | Lies, J. (2019): Clinical interview according to National Minimum Data Set Guidelines, in which sleep disturbance is defined as difficulty falling/staying asleep |
|  |  | Lies, J. (2020): ISI≥10 and/or sleep diary calculated SE≤85% |
|  |  | Loutan, L. (1999): Rating to insomnia-related item of HTQ based on DSM-III-R |
|  |  | Montgomery, E. (2001): Answered "frequent", "sometimes" or "rare" to question regarding difficulty staying asleep |
|  |  | Schnyder, U. (2015): Clinical interview according to DSM-IV or 5, in which sleep disturbance is defined as difficulty falling/staying asleep |
|  |  | Weaver, T.L. (2008): Answered "yes" to difficulty falling/staying asleep |
|  | Worries or behaviors that impair sleep homeostasis | Hinton, D.E. (2015): Defined as "insomnia caused by thinking a lot" |
|  |  | Itani, T. (2017): Answered "yes" to "were so worrie |
|  |  | Mangrio, E. (2020): Answered "more than usually" to "do you have difficulty sleeping due to anxiety?" |
| Nightmares | Nightmares as broad symptom complaint | Husni, M. (2001): Answered "1 to 5 times", "6 to 10 times" or "almost daily" to having "nightmares about escape circumstances from Kurdistan or oppression in Kurdistan in the last six months" |
|  |  | Cernovsky, Z. (1988): Answered "yes" to having nightmares |
|  |  | Bronstein, I. (2013): Answered "everyday", "3 to 6 times", or "1 to 2 times" to having "nightmares in the last 2 weeks" |
|  |  | Montgomery, E. (2001): Answered "frequent", "sometimes" or "rare" to frequency of nightmares |
|  |  | Kinzie, J.D. (1986): Reported having "nightmares" during clinical interview |

|  |  | Berkson, S.Y. (2014): Answered "daily" when asked about the frequency of nightmares |
| --- | --- | --- |
|  |  | Trohl, U. (2021): Noted as a symptom complaint; "in the course of the assessment interview, the psychologist specifically asked for ‘disorder-relevant psychopathological symptoms'" |
|  | Clinical or diagnostic  assessmen  t of  nightmares | Hinton, D.E. (2009): Clinical interview according to the DSM-IV |
|  |  | Abuali, M. (2024): Clinical interview according to the CDC domestic refugee guidance |
|  |  | Carlsson, J.M. (2006): Baseline; rating to nightmares-related item of HTQ based on DSM-III-R |
| Sleep quality | General sleep quality/dist urbance measurem  ent | Rizzi, D. (2022): Rated "sleep disturbances" on a Likert scale of 0  ("none") to 4 ("very severe") adapted from the DSM-5 Level 1 Cross-Cutting Symptom Measure |
|  |  | Pfeiffer, E. (2019): Rated "sleep disturbance" on a 4-point response scale ranging from 0 = ‘never’ to 3 = ‘almost always’ as an item from Child and Adolescent Trauma Screen |
|  |  | Müller, L.R.F. (2021): Rated "sleep quality" using a single item on a visual analogue scale ranging from 0 (“very well”) to 100 (“very badly”) |
|  | Specific sleep  difficulties | Schlechter, P. (2021): Rated trouble falling, staying asleep and nightmares recurrence on a Likert-Type scale item from 0 (“not at all”) to 4 (“extremely”) in the Impact of Event Scale-Revised |
|  |  | Zaheer, K. (2022): Rated "difficulty sleeping" on a 5-point Likert scale of “very often,” “occasionally,” “rarely,” “never,” or “I don't know” as an item of Oral Health and Well-Being questionnaire |
|  |  | Mölsä, M. (2014): Rated two questions concerning the difficulty to fall asleep and maintain sleep on a Likert scale of 1 ("no problem") to 4 ("nearly always") |
|  |  | Vinson, G.A. (2012): Rated "sleep difficulties" on a 4-point Likert scale from 1 ("not at all") to 4 ("often") as an item from Posttraumatic Stress Diagnostic Scale |
|  |  | Lindheimer, N. (2020): Rated trouble falling or staying asleep, or sleeping too much on a 4 point Likert scale, ranging from “not at all”  (0) to “nearly every day” (3) as items from PHQ-9 |
|  |  | Giesebrecht, J. (2022): Rated “feeling tired or having low energy” and “trouble sleeping” on a 3-point Likert scale ranging from “0” (not at all) to “2” (bothered a lot) as items from PHQ-15 |
|  |  | Mootoo, C. (2019): Rated distress from recurrent nightmares and difficulty falling asleep on a scale of 0 ("not at all distressing") to 4 ("extremely distressing") based on DSM-5 constructs |

## Table 6. Meta-regression models.

##

| Outcome | Model | k | I² (%) | R² (%) | AIC | p-value (QM) |
| --- | --- | --- | --- | --- | --- | --- |
| **Sleep adversities prevalence – adults** | Instrument type | 30 | 98.3 | 0.9 | 97.42 | 0.6669 |
|  | Nature of exposure | 28 | 98.2 | 12.5 | 91.20 | 0.1749 |
|  | Healthcare availability | 30 | 98.3 | 4.8 | 97.91 | 0.4660 |
|  | Origin region | 30 | 97.3 | 39.1 | 100.48 | 0.3446 |
|  | Host region | 30 | 97.5 | 26.7 | 104.97 | 0.6747 |
|  | Percentage of females | 29 | 98.5 | 0.1 | 93.83 | 0.8779 |
|  | Mean age | 27 | 98.3 | 6.0 | 87.45 | 0.2338 |
|  | Years since resettlement | 20 | 98.3 | 11.4 | 70.91 | 0.5887 |
|  | Study quality | 30 | 98.4 | 0.3 | 99.51 | 0.9500 |
|  | Origin region + Host region + Years since resettlement | 20 | 0.0 | 100.0 | 7.97 | 0.0065 |
| Sleep adversities prevalence – children | Instrument type | 15 | 99.0 | 4.4 | 56.18 | 0.4594 |
|  | Nature of exposure | 14 | 99.2 | 5.1 | 54.69 | 0.7529 |
|  | Healthcare availability | 15 | 98.9 | 8.0 | 57.64 | 0.6203 |
|  | Origin region | 15 | 98.2 | 23.7 | 61.06 | 0.7594 |
|  | Host region | 15 | 98.4 | 27.1 | 58.56 | 0.5386 |
|  | Percentage of females | 15 | 99.5 | 0.8 | 56.70 | 0.7362 |
|  | Mean age | 15 | 99.1 | 0.4 | 56.78 | 0.8276 |
|  | Years since resettlement | 8 | 95.4 | 65.0 | 27.02 | 0.0956 |
|  | Study quality | 15 | 99.0 | 5.9 | 57.98 | 0.7100 |

##

## Table 7. PRISMA Checklist

| **Section and**  **Topic** | **Item #** | **Checklist item** | **Location where item is reported** |
| --- | --- | --- | --- |
| **TITLE** | | |  |
| Title | 1 | Identify the report as a systematic review. | pg. 1 |
| **ABSTRACT** | | |  |
| Abstract | 2 | See the PRISMA 2020 for Abstracts checklist. | pg. 2 |
| **INTRODUCTION** | | |  |
| Rationale | 3 | Describe the rationale for the review in the context of existing knowledge. | pg 3 - 4. |
| Objectives | 4 | Provide an explicit statement of the objective(s) or question(s) the review addresses. | pg. 4 |
| **METHODS** | | |  |
| Eligibility criteria | 5 | Specify the inclusion and exclusion criteria for the review and how studies were grouped for the syntheses. | pg. 6 and 7 |
| Information sources | 6 | Specify all databases, registers, websites, organisations, reference lists and other sources | pg. 7 |

|  |  | searched or consulted to identify studies. Specify the date when each source was last searched or consulted. |  |
| --- | --- | --- | --- |
| Search strategy | 7 | Present the full search strategies for all databases, registers and websites, including any filters and limits used. | S1_Table (cited pg. 7) |
| Selection process | 8 | Specify the methods used to decide whether a study met the inclusion criteria of the review, including how many reviewers screened each record and each report retrieved, whether they worked independently, and if applicable, details of automation tools used in the process. | pg. 7 and 8 |
| Data collection process | 9 | Specify the methods used to collect data from reports, including how many reviewers collected data from each report, whether they worked independently, any processes for obtaining or confirming data from study investigators, and if applicable, details of automation tools used in the process. | pg. 8 and 9 |
| Data items | 10a | List and define all outcomes for which data were sought. Specify whether all results that were compatible with each outcome domain in each study were sought (e.g. for all measures, time points, analyses), and if not, the methods used to decide which results to collect. | pg. 8 and 9 |
|  | 10b | List and define all other variables for which data were sought (e.g. participant and intervention characteristics, funding sources). Describe any assumptions made about any missing or unclear information. | pg. 8 and 9. |
| Study risk of bias assessment | 11 | Specify the methods used to assess risk of bias in the included studies, including details of the tool(s) used, how many reviewers assessed each study and whether they worked independently, and if applicable, details of automation tools used in the process. | pg. 9 |
| Effect  measures | 12 | Specify for each outcome the effect measure(s) (e.g. risk ratio, mean difference) used in the synthesis or presentation of results. | pg. 10 |

| Synthesis methods | 13a | Describe the processes used to decide which studies were eligible for each synthesis (e.g. tabulating the study intervention characteristics and comparing against the planned groups for each synthesis (item #5)). | pg 8 and 10. |
| --- | --- | --- | --- |
|  | 13b | Describe any methods required to prepare the data for presentation or synthesis, such as handling of missing summary statistics, or data conversions. | pg 10 and 11. |
|  | 13c | Describe any methods used to tabulate or visually display results of individual studies and syntheses. | pg. 10 and 11. |
|  | 13d | Describe any methods used to synthesize results and provide a rationale for the choice(s). If meta-analysis was performed, describe the model(s), method(s) to identify the presence and extent of statistical heterogeneity, and software package(s) used. | pg 10 and 11. |
|  | 13e | Describe any methods used to explore possible causes of heterogeneity among study results (e.g. subgroup analysis, meta-regression). | pg. 10 and 11 |
|  | 13f | Describe any sensitivity analyses conducted to assess robustness of the synthesized results. | pg. 11 |
| Reporting bias assessment | 14 | Describe any methods used to assess risk of bias due to missing results in a synthesis (arising from reporting biases). | pg. 11 |
| Certainty assessment | 15 | Describe any methods used to assess certainty (or confidence) in the body of evidence for an outcome. | pg. 10 |
| **RESULTS** | | |  |
| Study selection | 16a | Describe the results of the search and selection process, from the number of records identified in the search to the number of studies included in the review, ideally using a flow diagram. | pg. 13 |
|  | 16b | Cite studies that might appear to meet the inclusion criteria, but which were excluded, and explain why they were excluded. | pg. 13 |

| Study characteristi cs | 17 | Cite each included study and present its characteristics. | pg 12, 13.  and Table 1 |
| --- | --- | --- | --- |
| Risk of bias in studies | 18 | Present assessments of risk of bias for each included study. | pg 23 and Table 1 |
| Results of individual studies | 19 | For all outcomes, present, for each study: (a) summary statistics for each group (where appropriate) and (b) an effect estimate and its precision (e.g. confidence/credible interval), ideally using structured tables or plots. | pg 23 - 26. |
| Results of syntheses | 20a | For each synthesis, briefly summarise the characteristics and risk of bias among contributing studies. | pg. 23 |
|  | 20b | Present results of all statistical syntheses conducted. If meta-analysis was done, present for each the summary estimate and its precision (e.g.  confidence/credible interval) and measures of statistical heterogeneity. If comparing groups, describe the direction of the effect. | pg 23 - 30.    and S1_File |
|  | 20c | Present results of all investigations of possible causes of heterogeneity among study results. | pg 25 - 28.    and S1_FIle |
|  | 20d | Present results of all sensitivity analyses conducted to assess the robustness of the synthesized results. | pg. 25    and S1_File |
| Reporting biases | 21 | Present assessments of risk of bias due to missing results (arising from reporting biases) for each synthesis assessed. | pg. 28 |
| Certainty of evidence | 22 | Present assessments of certainty (or confidence) in the body of evidence for each outcome assessed. | Not performed |
| **DISCUSSION** | | |  |
| Discussion | 23a | Provide a general interpretation of the results in the context of other evidence. | pg 30 - 33 |
|  | 23b | Discuss any limitations of the evidence included in the review. | pg. 33 |
|  | 23c | Discuss any limitations of the review processes | pg 33 |
|  |  | used. |  |
|  | 23d | Discuss implications of the results for practice, policy, and future research. | pg. 34 and 35 |
| **OTHER INFORMATION** | | |  |
| Registration and protocol | 24a | Provide registration information for the review, including register name and registration number, or state that the review was not registered. | pg. 5 |
|  | 24b | Indicate where the review protocol can be accessed, or state that a protocol was not prepared. | pg. 5 |
|  | 24c | Describe and explain any amendments to information provided at registration or in the protocol. | pg. 5 |
| Support | 25 | Describe sources of financial or non-financial support for the review, and the role of the funders or sponsors in the review. | Submission  System |
| Competing interests | 26 | Declare any competing interests of review authors. | Submission  System |
| Availability of data, code and other materials | 27 | Report which of the following are publicly available and where they can be found: template data collection forms; data extracted from included studies; data used for all analyses; analytic code; any other materials used in the review. | pg. 12 and S1_File. |
