# Supplementary material for: Insomnia and poor sleep quality in refugee and asylum-seeking populations: A systematic review and meta-analysis
Source: PLoS One. 2026 Jul 2;21(7):e0352964. doi: 10.1371/journal.pone.0352964 (PMC13327149; doi:10.1371/journal.pone.0352964)
Supplement: S1 Table — (DOCX) [file pone.0352964.s002.docx]

## **S1 Table. Search strategies.**

| PubMed | ("human migration" OR "transients and migrants" OR refugee OR refugees OR "emigration and immigration" OR "emigrants and immigrants" OR "undocumented immigrants" OR "asylum seeker*" OR migrant* OR immigrant* OR immigration* OR emigrant* OR emigration* OR "internal displace*" OR "displaced people" OR "displaced population*" OR "displaced person*" OR "displaced men" OR "displaced individual*" OR "Human Migration"[Mesh] OR "Transients and Migrants"[Mesh] OR "Refugees"[Mesh] OR "Emigration and Immigration"[Mesh] OR "Emigrants and Immigrants"[Mesh] OR "Undocumented Immigrants"[Mesh] OR refugee*) AND (sleep OR nonsleep OR "sleep disorders" OR "sleep disturbances" OR insomnia OR "sleep initiation and maintenance disorders" OR "disturbed sleep") |
| --- | --- |
| Embase | ('human migration'/exp OR 'human migration' OR 'transients and migrants'/exp OR 'transients and migrants' OR 'refugee'/exp OR refugee OR 'refugees'/exp OR refugees OR 'emigration and immigration'/exp OR 'emigration and immigration' OR 'emigrants and immigrants'/exp OR 'emigrants and immigrants' OR 'undocumented immigrants'/exp OR 'undocumented immigrants' OR 'asylum seeker*' OR migrant* OR immigrant* OR immigration* OR emigrant* OR emigration* OR 'internal displace*' OR 'displaced people' OR 'displaced population*' OR 'displaced person*' OR 'displaced men' OR 'displaced individual*') AND ('sleep'/exp OR sleep OR nonsleep OR 'sleep disorders'/exp OR 'sleep disorders' OR 'sleep disturbances'/exp OR 'sleep disturbances' OR 'insomnia'/exp OR insomnia OR 'sleep initiation and maintenance disorders'/exp OR 'sleep initiation and maintenance disorders' OR 'disturbed sleep') |
| Cochrane Library | ("human migration" OR "transients and migrants" OR refugee OR refugees OR "emigration and immigration" OR "emigrants and immigrants" OR "undocumented immigrants" OR asylum NEXT seeker* OR migrant* OR immigrant* OR immigration* OR emigrant* OR emigration* OR internal NEXT displace* OR "displaced people" OR displaced NEXT population* OR displaced NEXT person* OR "displaced men" OR displaced NEXT individual*) AND (sleep OR nonsleep OR "sleep disorders" OR "sleep disturbances" OR insomnia OR "sleep initiation and maintenance disorders" OR "disturbed sleep") |

# 
