# Supplementary material for: Insomnia and poor sleep quality in refugee and asylum-seeking populations: A systematic review and meta-analysis
Source: PLoS One. 2026 Jul 2;21(7):e0352964. doi: 10.1371/journal.pone.0352964 (PMC13327149; doi:10.1371/journal.pone.0352964)
Supplement: S2 Table — (DOCX) [file pone.0352964.s003.docx]

#

| **Table 2. Newcastle-Ottawa Scale (NOS) for cohort studies.** | | | | | |
| --- | --- | --- | --- | --- | --- |
| Note: A study can be awarded a maximum of one point for each numbered item within the Selection and Outcome categories. A maximum of two points can be given for Comparability **Selection:**  1) Representativeness of the exposed cohort   1. truly representative of the average in the community (1 point) 2. somewhat representative of the average in the community (1 point) 3. selected group of users eg nurses, volunteers 4. no description of the derivation of the cohort   2) Selection of the non exposed cohort   1. drawn from the same community as the exposed cohort (1 point) 2. drawn from a different source 3. no description of the derivation of the non exposed cohort   3) Ascertainment of exposure   1. secure record (eg surgical records) (1 point) 2. structured interview (1 point) 3. written self report 4. no description   4) Demonstration that outcome of interest was not present at start of study   1. yes (1 point) 2. no | | | | | |
| **Comparability:**  1) Comparability of cohorts on the basis of the design or analysis   1. study controls for (select the most important factor) (1 point) 2. study controls for any additional factor (1 point) (This criteria could be modified to indicate specific control for a second important factor.) | | | | | |
| **Outcome**:  1) Assessment of outcome   1. independent blind assessment (1 point) 2. record linkage (1 point) 3. self report 4. no description   2) Was follow-up long enough for outcomes to occur   1. yes (select an adequate follow up period for outcome of interest) (1 point) 2. no   3) Adequacy of follow up of cohorts   1. complete follow up - all subjects accounted for (1 point) 2. subjects lost to follow up unlikely to introduce bias - small number lost follow up, or description provided of those lost (1 point) 3. follow up rate inadequate and no description of those lost 4. no statement | | | | | |
|  | | | | | |

# 
