# Supplementary material for: Insomnia and poor sleep quality in refugee and asylum-seeking populations: A systematic review and meta-analysis
Source: PLoS One. 2026 Jul 2;21(7):e0352964. doi: 10.1371/journal.pone.0352964 (PMC13327149; doi:10.1371/journal.pone.0352964)
Supplement: S3 Table — (DOCX) [file pone.0352964.s004.docx]

#

| **Table 3. Newcastle-Ottawa Scales (NOS) adapted for cross-sectional studies.** | | | | | | |
| --- | --- | --- | --- | --- | --- | --- |
| **Selection:** (Maximum 5 points)  1) Representativeness of the sample:   1. Truly representative of the average in the target population. (all subjects or random sampling) (1 point) 2. Somewhat representative of the average in the target population. (nonrandom sampling) (1 point) 3. Selected group of users. 4. No description of the sampling strategy.   2) Sample size:   1. Justified and satisfactory. (1 point) 2. Not justified.   3) Non-respondents:   1. Comparability between respondents and non-respondents characteristics is established, and the response rate is satisfactory. (1 point) 2. The response rate is unsatisfactory, or the comparability between respondents and non-respondents is unsatisfactory. 3. No description of the response rate or the characteristics of the responders and the non-responders.   4) Ascertainment of the exposure (risk factor):   1. Validated measurement tool. (2 points) 2. Non-validated measurement tool, but the tool is available or described. (1 point) 3. No description of the measurement tool. | | | | | | |
| **Comparability**: (Maximum 2 points)  1) The subjects in different outcome groups are comparable, based on the study design or analysis. Confounding factors are controlled.   1. The study controls for the most important factor (select one). (1 point) 2. The study control for any additional factor. (1 point) | | | | | | |
| **Outcome**: (Maximum 3 points)  1) Assessment of the outcome:   1. Independent blind assessment. (2 points) 2. Record linkage. (2 points) 3. Self report. (1 point) 4. No description.   2) Statistical test:   1. The statistical test used to analyze the data is clearly described and appropriate, and the measurement of the association is presented, including confidence intervals an the probability level (p value). (1 point) 2. The statistical test is not appropriate, not described or incomplete.   Cross-sectional Studies: Very Good Studies: 9-10 points Good Studies: 7-8 points Satisfactory Studies: 5- 6 points Unsatisfactory Studies: 0 to 4 points | | | | | | |
|  | | | | | | |
