# Supplementary material for: Insomnia and poor sleep quality in refugee and asylum-seeking populations: A systematic review and meta-analysis
Source: PLoS One. 2026 Jul 2;21(7):e0352964. doi: 10.1371/journal.pone.0352964 (PMC13327149; doi:10.1371/journal.pone.0352964)
Supplement: S4 Table — (DOCX) [file pone.0352964.s005.docx]

| Table 4. Moderators literature gap. | | | | | | | | | | | | | | | | | | | | |
| --- | --- | --- | --- | --- | --- | --- | --- | --- | --- | --- | --- | --- | --- | --- | --- | --- | --- | --- | --- | --- |
| Study |  |  |  |  |  |  |  |  |  |  |  |  |  |  |  |  |  |  |  | % |
| Spanhel K, 2022 | RP | NA | RP | NR | RP | RP | RP | NA | RP | NR | NR | NA | NA | NA | RP | RP | RP | RP | RP | 58 |
| Sankari S, 2023 | NA | NA | RP | RP | RP | RP | RP | NA | NR | RP | NR | NA | NA | NA | RP | RP | RP | NR | RP | 53 |
| Richter K, 2018 | NA | NA | RP | RP | RP | RP | RP | NA | NR | NR | NR | NA | NA | NA | RP | RP | RP | RP | RP | 53 |
| Özdemir PG, 2021 | NA | NA | RP | NR | RP | RP | RP | NA | RP | RP | NR | NA | NA | NA | RP | RP | RP | RP | NR | 53 |
| Lee J, 2021 | NA | NA | NR | NR | RP | RP | RP | NA | NR | NR | RP | NA | NA | RP | RP | RP | RP | RP | RP | 53 |
| Sandahl H, 2020 | RP | RP | RP | NR | RP | RP | RP | NA | RP | RP | NR | NA | NA | NA | RP | RP | RP | RP | RP | 68 |
| Schumm H, 2023 | NA | RP | RP | RP | RP | RP | NR | NA | RP | RP | NR | NA | NA | NA | RP | RP | RP | RP | RP | 63 |
| Bruck D, 2021 | NA | NA | NR | RP | RP | RP | RP | NA | RP | RP | NR | NA | NA | RP | RP | RP | RP | RP | RP | 63 |
| Lies J, 2021 | NA | NA | NR | RP | RP | RP | RP | NA | RP | RP | NR | NA | NA | NA | RP | RP | RP | RP | RP | 58 |
| Al-Smadi AM, 2019 | NA | NA | RP | RP | RP | RP | RP | NA | RP | RP | NR | NA | NA | NA | RP | RP | RP | RP | NR | 58 |
| Meurling J, 2023 | NA | NA | NR | NR | RP | RP | RP | NA | RP | NR | NR | NA | NA | NA | RP | RP | RP | RP | RP | 47 |
| Park J, 2019 | NA | NA | NR | NR | RP | RP | RP | NA | NR | NR | NR | RP | NA | NA | RP | RP | RP | RP | NR | 42 |
| Gammoh OS, 2024a | NA | NA | RP | RP | RP | RP | RP | NA | RP | RP | NR | NA | NA | NA | RP | RP | RP | NR | RP | 58 |
| Lies J, 2019 | NA | NA | RP | RP | RP | RP | RP | RP | NA | NA | NR | NR | NR | NA | RP | RP | RP | RP | RP | 58 |
| Carlsson JM, 2006 | RP | NA | RP | NR | RP | RP | RP | NA | RP | RP | NR | NA | NA | NA | RP | RP | RP | RP | RP | 63 |
| Aldukhail S, 2023 | NR | NA | RP | RP | RP | RP | RP | RP | RP | RP | NR | NR | NR | NA | RP | RP | RP | RP | NR | 63 |
| Rizzi D, 2022 | NA | NA | RP | RP | RP | RP | RP | NA | NR | NR | RP | NA | NA | NA | RP | RP | RP | RP | NR | 53 |
| Parvez A, 2023 | RP | NA | RP | RP | RP | RP | RP | NA | RP | RP | NR | NA | NA | NA | RP | RP | RP | RP | RP | 68 |

| Trohl U, 2021 | RP | NA | RP | RP | RP | RP | RP | NA | NR | NR | RP | NA | NA | NA | RP | RP | RP | RP | RP | 63 |
| --- | --- | --- | --- | --- | --- | --- | --- | --- | --- | --- | --- | --- | --- | --- | --- | --- | --- | --- | --- | --- |
| Tay AK, 2015 | NA | NA | NR | RP | RP | RP | RP | NA | RP | RP | RP | NA | NA | NA | RP | RP | RP | RP | RP | 63 |
| Tamblyn JM, 2011 | RP | NA | RP | RP | RP | RP | RP | NA | NR | NR | NR | NA | NA | NA | RP | RP | RP | RP | RP | 58 |
| Loutan L, 1999 | NA | NA | RP | NR | RP | RP | RP | NA | RP | NR | RP | NA | NA | NA | RP | RP | RP | RP | NR | 53 |
| Lee YG, 2016 | NA | NA | NR | NR | RP | RP | RP | NA | NR | NR | NR | NA | NA | RP | RP | RP | RP | RP | RP | 47 |
| Honkala E, 1992 | NA | NA | RP | RP | RP | RP | RP | RP | NA | NA | NR | NR | NR | NA | RP | RP | RP | RP | NR | 53 |
| Westermeyer JJ, 2010 | NA | NA | NR | RP | RP | RP | RP | NA | NR | NR | NR | NA | NA | NA | RP | RP | RP | RP | RP | 47 |
| Gulden A, 2010 | NA | NA | NR | RP | RP | RP | RP | NA | RP | NR | NR | NA | NA | NA | RP | RP | RP | RP | RP | 53 |
| Gowin M, 2017 | NA | NA | NR | NR | RP | RP | RP | NA | RP | NR | RP | NA | NA | NA | RP | RP | NA | RP | NR | 42 |
| Schlechter P, 2021 | NA | NA | NR | NR | RP | RP | RP | NA | NR | NR | NR | NA | NA | NA | RP | RP | RP | RP | NR | 37 |
| Mootoo C, 2019 | NA | NA | RP | RP | RP | RP | RP | NA | NR | NR | NR | NA | NA | NA | RP | RP | RP | RP | NR | 47 |
| Schnyder U, 2015 | NA | NA | RP | NR | RP | RP | RP | NA | RP | NR | NR | NA | NA | NA | RP | RP | RP | RP | RP | 53 |
| Weaver TL, 2008 | NA | NA | RP | NR | RP | RP | RP | NA | RP | RP | NR | NA | NA | NA | RP | RP | RP | RP | RP | 58 |
| Mölsä M, 2014 | NA | NA | RP | RP | RP | RP | RP | NA | RP | RP | NR | NA | NA | RP | RP | RP | RP | RP | RP | 68 |
| Vinson GA, 2012 | NA | NA | RP | RP | RP | RP | RP | NA | RP | NR | RP | NA | NA | NA | RP | RP | RP | RP | NR | 58 |
| Zaheer K, 2022 | NA | NA | RP | RP | RP | RP | RP | NA | RP | NR | NR | NA | NA | NA | RP | RP | RP | RP | NR | 53 |
| Lindheimer N, 2020 | NA | NA | RP | RP | RP | RP | RP | NA | RP | NR | NR | NA | NA | NA | RP | RP | RP | RP | NR | 53 |
| Giesebrecht J, 2022 | NA | NA | RP | RP | RP | RP | RP | NA | RP | NR | NR | NA | NA | NA | RP | RP | RP | RP | RP | 58 |
| Abuali M, 2024 | NA | NA | RP | RP | RP | RP | RP | RP | RP | NA | RP | NR | NR | NA | RP | RP | RP | RP | NR | 63 |
| Montgomery E, 2001 | NA | NA | NR | RP | RP | RP | RP | RP | NA | NA | RP | NR | NR | NA | RP | RP | RP | RP | NR | 53 |
| Pfeiffer E, 2019 | NA | NA | RP | NR | RP | RP | RP | RP | NA | NA | RP | NR | RP | NA | RP | RP | RP | RP | RP | 63 |
| Genton PC, 2019 | NA | NA | RP | NR | RP | RP | RP | RP | NA | NA | RP | NR | RP | NA | RP | RP | RP | RP | NR | 58 |
| Ceri V, 2016 | NA | NA | RP | RP | RP | RP | RP | RP | NA | NA | RP | NR | NR | NA | RP | RP | RP | RP | NR | 58 |
| Eiset AH, 2020 | NA | NA | RP | RP | RP | RP | RP | RP | NA | NA | RP | NR | RP | NA | RP | RP | RP | RP | NR | 63 |
| Schumacher L, 2021 | NR | NA | RP | NR | RP | RP | RP | RP | NA | NA | RP | NR | RP | NA | RP | RP | RP | RP | RP | 63 |
| Hjern A, 2019 | NA | NA | RP | RP | RP | RP | RP | RP | NA | NA | RP | RP | RP | NA | RP | RP | RP | RP | NR | 68 |
| Hjern A, 1991 | NR | NA | RP | NR | RP | RP | RP | RP | NA | NA | RP | NR | RP | NA | RP | RP | RP | RP | NR | 58 |
| Nasıroğlu S, 2018 | NA | NA | RP | RP | RP | RP | RP | RP | NA | NA | NA | RP | RP | NA | RP | RP | RP | RP | NR | 63 |
| Husni M, 2001 | NA | NA | NR | NR | RP | RP | RP | NA | RP | RP | NR | NA | NA | NA | RP | RP | RP | RP | RP | 53 |
| Hinton DE, 2009 | NA | RP | RP | RP | RP | RP | RP | NA | RP | NR | RP | NA | NA | NA | RP | RP | RP | RP | NR | 63 |

| Cernovsky Z, 1988 | NA | NA | NR | RP | RP | RP | RP | NA | RP | RP | RP | NA | NA | NA | RP | RP | RP | RP | NR | 58 |
| --- | --- | --- | --- | --- | --- | --- | --- | --- | --- | --- | --- | --- | --- | --- | --- | --- | --- | --- | --- | --- |
| Lee S, 2021 | NA | NA | RP | RP | RP | RP | RP | NA | NA | NR | NA | NA | NA | NA | RP | RP | RP | RP | NR | 47 |
| Berkson SY, 2014 | NR | NA | RP | RP | RP | RP | RP | NA | RP | NR | NA | NA | NA | NA | RP | RP | RP | RP | NR | 53 |
| Bronstein I, 2013 | NA | NA | NR | RP | RP | RP | RP | RP | NA | NA | RP | NR | RP | NA | RP | RP | RP | RP | RP | 63 |
| Simich L, 2006 | NA | NA | NR | RP | RP | RP | RP | NA | RP | RP | RP | NA | NA | NA | RP | RP | RP | RP | RP | 63 |
| Müller LRF, 2021 | NR | NA | RP | RP | RP | RP | RP | NR | NA | NA | RP | RP | NR | NA | RP | RP | RP | RP | RP | 63 |
| Mangrio E, 2020 | NA | NA | RP | NR | RP | RP | RP | NA | RP | NR | NR | NA | NA | NA | RP | RP | RP | RP | NR | 47 |
| Ku SY, 2006 | NA | NA | RP | RP | RP | RP | NR | NA | RP | NR | NR | NA | NA | NA | RP | RP | RP | RP | NR | 47 |
| Knappe F, 2023 | NR | NA | RP | RP | RP | RP | RP | NA | RP | RP | RP | NA | NA | NA | RP | RP | RP | RP | RP | 68 |
| Itani T, 2017 | NA | NA | RP | RP | RP | RP | RP | RP | NA | NA | NR | RP | NR | NA | RP | RP | RP | NA | NR | 53 |
| Hinton DE, 2015 | NA | NA | RP | RP | RP | RP | RP | NA | NR | NR | NR | NA | NA | NA | RP | RP | RP | NA | NR | 42 |
| Hinton DE, 2005 | NA | NA | RP | RP | RP | RP | RP | NA | NR | NR | NR | NA | NA | NA | RP | RP | RP | RP | NR | 47 |
| Gammoh OS, 2024b | NA | NA | RP | RP | RP | RP | RP | NA | RP | RP | NR | NA | NA | NA | RP | RP | RP | NA | NR | 53 |
| Boiko DI, 2024 | NA | NA | NR | NR | RP | NR | RP | NA | NR | RP | NR | NA | NA | RP | RP | RP | RP | RP | NR | 42 |
| Thabet AA, 1999 | NA | NA | NR | RP | RP | NR | RP | RP | NA | NA | NR | RP | NR | NA | RP | RP | RP | RP | NR | 47 |
| Kinzie JD, 1986 | NA | NA | RP | NR | RP | RP | RP | RP | NA | NA | RP | RP | RP | NA | RP | RP | RP | RP | RP | 68 |
| Realmuto GM, 1992 | NA | NA | NR | NR | RP | RP | RP | RP | NA | RP | RP | RP | NR | NA | RP | RP | RP | RP | NR | 58 |
| Gammoh OS, 2024c | NA | NA | RP | RP | RP | RP | RP | NA | RP | RP | RP | NA | NA | NA | RP | RP | RP | RP | NR | 63 |
| % | 9 | 5 | 71 | 67 | 100 | 97 | 97 | 27 | 52 | 33 | 38 | 12 | 14 | 8 | 100 | 100 | 98 | 92 | 47 |  |
| Note: NA = "Not applicable", NR = "Not reported", RP="Reported" | | | | | | | | | |  |  |  |  |  |  |  |  |  |  |  |
