# Supplementary material for: Insomnia and poor sleep quality in refugee and asylum-seeking populations: A systematic review and meta-analysis
Source: PLoS One. 2026 Jul 2;21(7):e0352964. doi: 10.1371/journal.pone.0352964 (PMC13327149; doi:10.1371/journal.pone.0352964)
Supplement: S5 Table — (DOCX) [file pone.0352964.s006.docx]

| Table 5. Terminology and definitions. | | | | | | | | | | | | | | | |
| --- | --- | --- | --- | --- | --- | --- | --- | --- | --- | --- | --- | --- | --- | --- | --- |
| Terminology | Description category | Definition | | | | | | | | | | | | | |
| Sleep adversities | Broad description on impaired sleep homeostasis grouping multiple phenomenological instances | Abuali, M. (2014): Reported 'poor sleep' in a clinical interview according to the CDC domestic refugee guidance (p. 1) | | | | | | | | | | | | | |
|  |  | Ceri, V. (2016): Answered "yes" to having "sleep problems", such as problems falling asleep, frequent awakenings, and parasomnias like somnambulism and nightmares | | | | | | | | | | | | | |
|  |  | Eiset, A.H. (2020): Answered "yes" to having "sleep problems", without further detail | | | | | | | | | | | | | |
|  |  | Giesebrecht, J. (2022): Answered "several days", "more than half the days" or "nearly every day"to having "trouble sleeping" according to PHQ-9 item | | | | | | | | | | | | | |
|  |  | Gowin, M. (2017): Said to be diagnosed "by a professional", without further detail | | | | | | | | | | | | | |
|  |  | Gulden, A. (2010): Answered "yes" to having "sleep problems", such as insomnia, nightmares, daytime sleepiness | | | | | | | | | | | | | |
|  |  | Hjern, A. (2019): Answered "yes" to having "sleeping problems"; to be recorded as yes, mental health problems, including sleeping disturbances, had to be severe enough that the nurse judged them to impair the well-being of the child on a daily basis. | | | | | | | | | | | | | |
|  |  | Kinzie, J.D. (1986): Reported "trouble sleeping" du | | | | | | | | | | | | | |
|  |  | Lee, Y.G. (2016): According to ICD-10; participants experiencing at least one of the three types of insomnia (initial, maintenance, and terminal) ≥3/week were classified as having significant insomnia. | | | | | | | | | | | | | |
|  |  | Mangrio, E. (2020): Answered "bad" to the questio | | | | | | | | | | | | | |
|  |  | Nasıroğlu, S. (2018): Said to be diagnosed "by a professional", without further detail | | | | | | | | | | | | | |
|  |  | Parvez, A. (2023): According to ICD-10; "listed as having the diagnosis if made by the primary care physician" | | | | | | | | | | | | | |
|  |  | Realmuto, G.M. (1992): Answered "yes" to the item of Sleep Disturbance of an adapted version of the CPTSD-RI scale | | | | | | | | | | | | | |
|  |  | Richter, K. (2018): According to ICD-10; tried to establish the difference between insomnia symptoms and disease; the subject reported that their insomnia bothered them much more than the depressive or anxious mood and that better sleep would also improve their mood | | | | | | | | | | | | | |
|  |  | Schumacher, L. (2021): Answered "yes" to having "sleep problems", such as disrupted sleep patterns or insomnia that occur as part of the arousal symptoms following a traumatic event | | | | | | | | | | | | | |
|  |  | Tamblyn, J.M. (2011): Diagnostic according to DSM-IV TR criteria, by the RMSC licensed professional counselor, licensed clinical social worker, or psychiatrist; "insomnia as persistent difficulty with falling or staying asleep despite trying to do so" | | | | | | | | | | | | | |
|  |  | Tay, A.K. (2015): Yes or no to insomnia-related items according to DSM-IV and 5 | | | | | | | | | | | | | |
|  |  | Thabet, A.A. (1999): Answered "few", "some", "frequent" or "most of the time" to the item D1 (Sleep Disturbance) of the scale CPTSD-RI | | | | | | | | | | | | | |
|  |  | Trohl, U. (2021): Noted as a symptom complaint; "in the course of the assessment interview, the psychologist specifically asked for ‘disorder-relevant psychopathological symptoms'" | | | | | | | | | | | | | |
|  |  | Westermeyer, J.J. (2010): Answered "yes" to having "sleep problems", such as insomnia, nightmares, daytime sleepiness | | | | | | | | | | | | | |
|  |  | Zaheer, K. (2022): Answered "very often", "occasionally" and "rarely" to having "problems sleeping" according to Oral Health and Well-Being questionnaire item from the American Dental Association | | | | | | | | | | | | | |
|  | Clinical insomnia as per validated scale cut-offs | Al-Smadi, A.M. (2019): Defined as ISI>14 | | | | | | | | | | | | | |
|  |  | Boiko, D.I. (2024): Defined as ISI>14 | | | | | | | | | | | | | |
|  |  | Bruck, D. (2021): Defined as ISI>14 | | | | | | | | | | | | | |
|  |  | Gammoh, O.S. (2024a): Defined as "cutoff score of >14 indicates severe insomnia symptoms" | | | | | | | | | | | | | |
|  |  | Gammoh, O.S. (2024b): Defined as "cutoff score of >14 indicates severe insomnia symptoms" | | | | | | | | | | | | | |
|  |  | Gammoh, O.S. (2024c): Defined as "cutoff score of >14 indicates severe insomnia symptoms" | | | | | | | | | | | | | |
|  |  | Knappe, F. (2023): Defined as "cutoff score of >14 indicates severe insomnia symptoms" | | | | | | | | | | | | | |
|  |  | Lies, J. (2021): Defined as ISI>14; estimated assuming normal distribution as raw data wasn't available through contact | | | | | | | | | | | | | |
|  |  | Meurling, J. (2023): Defined as ISI>14 | | | | | | | | | | | | | |
|  |  | Park, J. (2019): Probably as ISI>14; the study claimed that 14 subjects surpassed the "cut-off" score, which commonly is 14 points | | | | | | | | | | | | | |
|  |  | Spanhel, K. (2022): At baseline, defined as ISI>14 | | | | | | | | | | | | | |
|  | Disorders of initiating or maintaining sleep | Aldukhail, S. (2023): Answered "yes" to difficulty staying asleep | | | | | | | | | | | | | |
|  |  | Carlsson, J.M. (2006): Baseline; rating to insomnia-related item of HTQ based on DSM-III-R | | | | | | | | | | | | | |
|  |  | Genton, P.C. (2019): Diagnosed accordingly to ICD-10 | | | | | | | | | | | | | |
|  |  | Hjern, A. (1991): Answered "yes" to having “nightmares at least twice a week, night terrors at least once a month, often waking up and not being able to fall asleep without parental assistance and/or often having difficulties of going to sleep at night." | | | | | | | | | | | | | |
|  |  | Honkala, E. (1992): Answered "yes" to "waking up during the night without any reason" | | | | | | | | | | | | | |
|  |  | Lies, J. (2019): Clinical interview according to National Minimum Data Set Guidelines, in which sleep disturbance is defined as difficulty falling/staying asleep | | | | | | | | | | | | | |
|  |  | Lies, J. (2020): ISI≥10 and/or sleep diary calculated SE≤85% | | | | | | | | | | | | | |
|  |  | Loutan, L. (1999): Rating to insomnia-related item of HTQ based on DSM-III-R | | | | | | | | | | | | | |
|  |  | Montgomery, E. (2001): Answered "frequent", "sometimes" or "rare" to question regarding difficulty staying asleep | | | | | | | | | | | | | |
|  |  | Schnyder, U. (2015): Clinical interview according to DSM-IV or 5, in which sleep disturbance is defined as difficulty falling/staying asleep | | | | | | | | | | | | | |
|  |  | Weaver, T.L. (2008): Answered "yes" to difficulty falling/staying asleep | | | | | | | | | | | | | |
|  | Worries or behaviors that impair sleep homeostasis | Hinton, D.E. (2015): Defined as "insomnia caused by thinking a lot" | | | | | | | | | | | | | |
|  |  | Itani, T. (2017): Answered "yes" to "were so worrie | | | | | | | | | | | | | |
|  |  | Mangrio, E. (2020): Answered "more than usually" to "do you have difficulty sleeping due to anxiety?" | | | | | | | | | | | | | |
| Nightmares | Nightmares as broad symptom complaint | Husni, M. (2001): Answered "1 to 5 times", "6 to 10 times" or "almost daily" to having "nightmares about escape circumstances from Kurdistan or oppression in Kurdistan in the last six months" | | | | | | | | | | | | | |
|  |  | Cernovsky, Z. (1988): Answered "yes" to having nightmares | | | | | | | | | | | | | |
|  |  | Bronstein, I. (2013): Answered "everyday", "3 to 6 times", or "1 to 2 times" to having "nightmares in the last 2 weeks" | | | | | | | | | | | | | |
|  |  | Montgomery, E. (2001): Answered "frequent", "sometimes" or "rare" to frequency of nightmares | | | | | | | | | | | | | |
|  |  | Kinzie, J.D. (1986): Reported having "nightmares" during clinical interview | | | | | | | | | | | | | |

|  |  | Berkson, S.Y. (2014): Answered "daily" when asked about the frequency of nightmares | | | | | | | | | | | | | |
| --- | --- | --- | --- | --- | --- | --- | --- | --- | --- | --- | --- | --- | --- | --- | --- |
|  |  | Trohl, U. (2021): Noted as a symptom complaint; "in the course of the assessment interview, the psychologist specifically asked for ‘disorder-relevant psychopathological symptoms'" | | | | | | | | | | | | | |
|  | Clinical or diagnostic assessment of nightmares | Hinton, D.E. (2009): Clinical interview according to the DSM-IV | | | | | | | | | | | | | |
|  |  | Abuali, M. (2024): Clinical interview according to the CDC domestic refugee guidance | | | | | | | | | | | | | |
|  |  | Carlsson, J.M. (2006): Baseline; rating to nightmares-related item of HTQ based on DSM-III-R | | | | | | | | | | | | | |
| Sleep quality | General sleep quality/disturbance measurement | Rizzi, D. (2022): Rated "sleep disturbances" on a Likert scale of 0 ("none") to 4 ("very severe") adapted from the DSM-5 Level 1 Cross-Cutting Symptom Measure | | | | | | | | | | | | | |
|  |  | Pfeiffer, E. (2019): Rated "sleep disturbance" on a 4-point response scale ranging from 0 = ‘never’ to 3 = ‘almost always’ as an item from Child and Adolescent Trauma Screen | | | | | | | | | | | | | |
|  |  | Müller, L.R.F. (2021): Rated "sleep quality" using a single item on a visual analogue scale ranging from 0 (“very well”) to 100 (“very badly”) | | | | | | | | | | | | | |
|  | Specific sleep difficulties | Schlechter, P. (2021): Rated trouble falling, staying asleep and nightmares recurrence on a Likert-Type scale item from 0 (“not at all”) to 4 (“extremely”) in the Impact of Event Scale-Revised | | | | | | | | | | | | | |
|  |  | Zaheer, K. (2022): Rated "difficulty sleeping" on a 5-point Likert scale of “very often,” “occasionally,” “rarely,” “never,” or “I don't know” as an item of Oral Health and Well-Being questionnaire | | | | | | | | | | | | | |
|  |  | Mölsä, M. (2014): Rated two questions concerning the difficulty to fall asleep and maintain sleep on a Likert scale of 1 ("no problem") to 4 ("nearly always") | | | | | | | | | | | | | |
|  |  | Vinson, G.A. (2012): Rated "sleep difficulties" on a 4-point Likert scale from 1 ("not at all") to 4 ("often") as an item from Posttraumatic Stress Diagnostic Scale | | | | | | | | | | | | | |
|  |  | Lindheimer, N. (2020): Rated trouble falling or staying asleep, or sleeping too much on a 4 point Likert scale, ranging from “not at all” (0) to “nearly every day” (3) as items from PHQ-9 | | | | | | | | | | | | | |
|  |  | Giesebrecht, J. (2022): Rated “feeling tired or having low energy” and “trouble sleeping” on a 3-point Likert scale ranging from “0” (not at all) to “2” (bothered a lot) as items from PHQ-15 | | | | | | | | | | | | | |
|  |  | Mootoo, C. (2019): Rated distress from recurrent nightmares and difficulty falling asleep on a scale of 0 ("not at all distressing") to 4 ("extremely distressing") based on DSM-5 constructs | | | | | | | | | | | | | |
