# Supplementary material for: Insomnia and poor sleep quality in refugee and asylum-seeking populations: A systematic review and meta-analysis
Source: PLoS One. 2026 Jul 2;21(7):e0352964. doi: 10.1371/journal.pone.0352964 (PMC13327149; doi:10.1371/journal.pone.0352964)
Supplement: S6 Table — (DOCX) [file pone.0352964.s007.docx]

#

| **Table 6. Meta-regression models.**  \| **Outcome** \| **Model** \| **k** \| **I² (%)** \| **R² (%)** \| **AIC** \| **p-value (QM)** \| \| --- \| --- \| --- \| --- \| --- \| --- \| --- \| \| **Sleep adversities prevalence – adults** \| Instrument type \| 30 \| 98.3 \| 0.9 \| 97.42 \| 0.6669 \| \|  \| Nature of exposure \| 28 \| 98.2 \| 12.5 \| 91.20 \| 0.1749 \| \|  \| Healthcare availability \| 30 \| 98.3 \| 4.8 \| 97.91 \| 0.4660 \| \|  \| Origin region \| 30 \| 97.3 \| 39.1 \| 100.48 \| 0.3446 \| \|  \| Host region \| 30 \| 97.5 \| 26.7 \| 104.97 \| 0.6747 \| \|  \| Percentage of females \| 29 \| 98.5 \| 0.1 \| 93.83 \| 0.8779 \| \|  \| Mean age \| 27 \| 98.3 \| 6.0 \| 87.45 \| 0.2338 \| \|  \| Years since resettlement \| 20 \| 98.3 \| 11.4 \| 70.91 \| 0.5887 \| \|  \| Study quality \| 30 \| 98.4 \| 0.3 \| 99.51 \| 0.9500 \| \|  \| Origin region + Host region + Years since resettlement \| 20 \| 0.0 \| 100.0 \| 7.97 \| 0.0065 \| \| **Sleep adversities prevalence – children** \| Instrument type \| 15 \| 99.0 \| 4.4 \| 56.18 \| 0.4594 \| \|  \| Nature of exposure \| 14 \| 99.2 \| 5.1 \| 54.69 \| 0.7529 \| \|  \| Healthcare availability \| 15 \| 98.9 \| 8.0 \| 57.64 \| 0.6203 \| \|  \| Origin region \| 15 \| 98.2 \| 23.7 \| 61.06 \| 0.7594 \| \|  \| Host region \| 15 \| 98.4 \| 27.1 \| 58.56 \| 0.5386 \| \|  \| Percentage of females \| 15 \| 99.5 \| 0.8 \| 56.70 \| 0.7362 \| \|  \| Mean age \| 15 \| 99.1 \| 0.4 \| 56.78 \| 0.8276 \| \|  \| Years since resettlement \| 8 \| 95.4 \| 65.0 \| 27.02 \| 0.0956 \| \|  \| Study quality \| 15 \| 99.0 \| 5.9 \| 57.98 \| 0.7100 \| |
| --- | --- | --- | --- | --- | --- | --- | --- | --- | --- | --- | --- | --- | --- | --- | --- | --- | --- | --- | --- | --- | --- | --- | --- | --- | --- | --- | --- | --- | --- | --- | --- | --- | --- | --- | --- | --- | --- | --- | --- | --- | --- | --- | --- | --- | --- | --- | --- | --- | --- | --- | --- | --- | --- | --- | --- | --- | --- | --- | --- | --- | --- | --- | --- | --- | --- | --- | --- | --- | --- | --- | --- | --- | --- | --- | --- | --- | --- | --- | --- | --- | --- | --- | --- | --- | --- | --- | --- | --- | --- | --- | --- | --- | --- | --- | --- | --- | --- | --- | --- | --- | --- | --- | --- | --- | --- | --- | --- | --- | --- | --- | --- | --- | --- | --- | --- | --- | --- | --- | --- | --- | --- | --- | --- | --- | --- | --- | --- | --- | --- | --- | --- | --- | --- | --- | --- | --- | --- | --- | --- | --- |

Note: All missing data in the data frames were addressed using Multiple Imputation by Chained Equations (MICE) with predictive mean matching (PMM). Twenty imputed datasets were generated with a fixed random seed (42) to ensure reproducibility. For model testing purposes, the first imputed dataset was selected from the pool of twenty as a representative test case.
